# The plasmid-mediated evolution of the mycobacterial ESX (Type VII) secretion systems

**DOI:** 10.1186/s12862-016-0631-2

**Published:** 2016-03-15

**Authors:** Mae Newton-Foot, Robin Mark Warren, Samantha Leigh Sampson, Paul David van Helden, Nicolaas Claudius Gey van Pittius

**Affiliations:** Division of Molecular Biology and Human Genetics, Department of Biomedical Sciences, DST/NRF Centre of Excellence for Biomedical Tuberculosis Research/SAMRC Centre for Molecular and Cellular Biology, Faculty of Medicine and Health Sciences, Stellenbosch University, Stellenbosch, South Africa

**Keywords:** ESX, ESAT-6, Evolution, Mycobacterium, Plasmid, Type VII secretion system

## Abstract

**Background:**

The genome of *Mycobacterium tuberculosis* contains five copies of the ESX gene cluster, each encoding a dedicated protein secretion system. These ESX secretion systems have been defined as a novel Type VII secretion machinery, responsible for the secretion of proteins across the characteristic outer mycomembrane of the mycobacteria. Some of these secretion systems are involved in virulence and survival in *M. tuberculosis*; however they are also present in other non-pathogenic mycobacteria, and have been identified in some non-mycobacterial actinomycetes. Three components of the ESX gene cluster have also been found clustered in some gram positive monoderm organisms and are predicted to have preceded the ESX gene cluster.

**Results:**

This study used *in silico* and phylogenetic analyses to describe the evolution of the ESX gene cluster from the WXG-FtsK cluster of monoderm bacteria to the five ESX clusters present in *M. tuberculosis* and other slow-growing mycobacteria. The ancestral gene cluster, ESX-4, was identified in several nonmycomembrane producing actinobacteria as well as the mycomembrane-containing *Corynebacteriales* in which the ESX cluster began to evolve and diversify. A novel ESX gene cluster, ESX-4_EVOL_, was identified in some non-mycobacterial actinomycetes and *M. abscessus* subsp. *bolletii*. ESX-4_EVOL_ contains all of the conserved components of the ESX gene cluster and appears to be a precursor of the mycobacterial ESX duplications. Between two and seven ESX gene clusters were identified in each mycobacterial species, with ESX-2 and ESX-5 specifically associated with the slow growers. The order of ESX duplication in the mycobacteria is redefined as ESX-4, ESX-3, ESX-1 and then ESX-2 and ESX-5. Plasmid-encoded precursor ESX gene clusters were identified for each of the genomic ESX-3, -1, -2 and -5 gene clusters, suggesting a novel plasmid-mediated mechanism of ESX duplication and evolution.

**Conclusions:**

The influence of the various ESX gene clusters on vital biological and virulence-related functions has clearly influenced the diversification and success of the various mycobacterial species, and their evolution from the non-pathogenic fast-growing saprophytic to the slow-growing pathogenic organisms.

**Electronic supplementary material:**

The online version of this article (doi:10.1186/s12862-016-0631-2) contains supplementary material, which is available to authorized users.

## Background

The genome of *Mycobacterium tuberculosis* contains five ESX (or ESAT-6) gene clusters, named ESX-1, -2, -3, -4 and -5, which encode the Esx and PE/PPE proteins, various ATPases, membrane proteins, the mycosin proteases and other ESX-associated proteins [1, 2]. The ESX gene clusters have been the topic of extensive research following the discovery that the primary attenuating deletion of *M. bovis* BCG, region of difference 1 (RD1), includes part of ESX-1 [3–5]. The proteins encoded in each of the ESX gene clusters have been predicted to form dedicated protein secretion systems, the ESX secretion systems, which have since been defined as a Type VII secretion machinery responsible for the secretion of, amongst others, the Esx, PE and PPE proteins encoded in them, across the outer mycomembrane [6, 7].

The functions of the five *M. tuberculosis* ESX secretion systems appear to be distinct. ESX-1 is associated with virulence in *M. tuberculosis* [8–10], where it is involved in the inhibition of T-cell responses and phagosome maturation [11, 12], and assists in the escape of mycobacteria from the macrophage vacuole by ESAT-6-mediated perforation of the vacuolar membrane [13–16]. ESX-5 has also been linked to *M. tuberculosis* pathogenicity and is involved in modulating the host immune responses to maintain a persistent infection [15, 17, 18]. ESX-5 has furthermore been linked to the uptake of nutrients by increasing outer-membrane permeability in the slow-growing mycobacteria [19]. ESX-3 is essential for the *in vitro* growth of *M. tuberculosis* [20, 21], and is involved in divalent cation (iron and zinc) homeostasis [22, 23], and specifically iron uptake via the mycobactin iron acquisition pathway [21, 24]. The functions of ESX-2 and ESX-4 remain unknown.

The ESX gene clusters occur throughout the genus *Mycobacterium.* A previous study has proposed the order of duplication of the ESX gene clusters to be ESX-4, -1, -3, -2 and then -5*,* with ESX-5 exclusively associated with the slow-growing mycobacteria [2]. The non-pathogenic, fast-growing mycobacterium, *M. smegmatis,* contains three of the five *M. tuberculosis* ESX gene clusters, ESX-1, -3 and -4 [2]. In *M. smegmatis,* ESX-1 has been shown to be involved in conjugal DNA transfer [25, 26]. ESX-3 is also involved in iron homeostasis*,* however it has not been directly linked to zinc homeostasis, and is not essential in this organism [27]. Although there are distinct contrasts in the functions of these secretion systems in *M. smegmatis* and *M. tuberculosis*, the orthologous systems have been shown to share certain characteristics and to secrete both sets of substrates [25, 28, 29]. This suggests that the ESX secretion systems have retained conserved mechanisms, and that virulence-associated functions may have evolved subsequently, or be associated with specific substrates.

ESX gene clusters have also been identified in the genomes of closely related actinomycetes outside of the genus *Mycobacterium*, including *Nocardia, Streptomyces* and *Corynebacteria* [2, 6]. Furthermore, genes encoding two components of the ESX secretion system, the WXG (or Esx-like) and FtsK/SpoIIIE proteins, have been found clustered in some gram-positive monoderm genera such as *Bacillus*, *Listeria* and *Saccharomyces* [30]*.* Indeed, it has been suggested that ESX secretion systems occur outside of the *Mycolata* (species containing a mycomembrane-like outer membrane containing mycolic acids, including *Corynebacteria*, *Rhodococci*, *Nocardia* and *Mycobacteria*) and are therefore not typically involved in trans-mycomembrane secretion [31]. This, together with the absence of an identifiable component responsible for mycomembrane translocation, or an elucidated Type VII secretory mechanism, has generated some controversy, as some suggest that these are requirements for the designation of the ESX secretion systems as distinct Type VII secretion machineries [32].

Here we investigated the presence and absence of the ESX gene clusters in the genomes of the sequenced mycobacteria and other representative species from the class *Actinobacteria.* The phylogenetic relationship between these and the identified WXG-FtsK clusters of certain monoderm bacteria was determined in order to define the evolutionary history of the Type VII ESX secretion systems. In addition to the five ESX gene clusters which were previously identified, ESX gene clusters were identified on plasmids within several species of mycobacterium, and shown to precede the genomic ESX duplications. A model is proposed for the plasmid-mediated duplication and evolution of the ESX gene clusters.

## Results and discussion

**Fig. 1:**
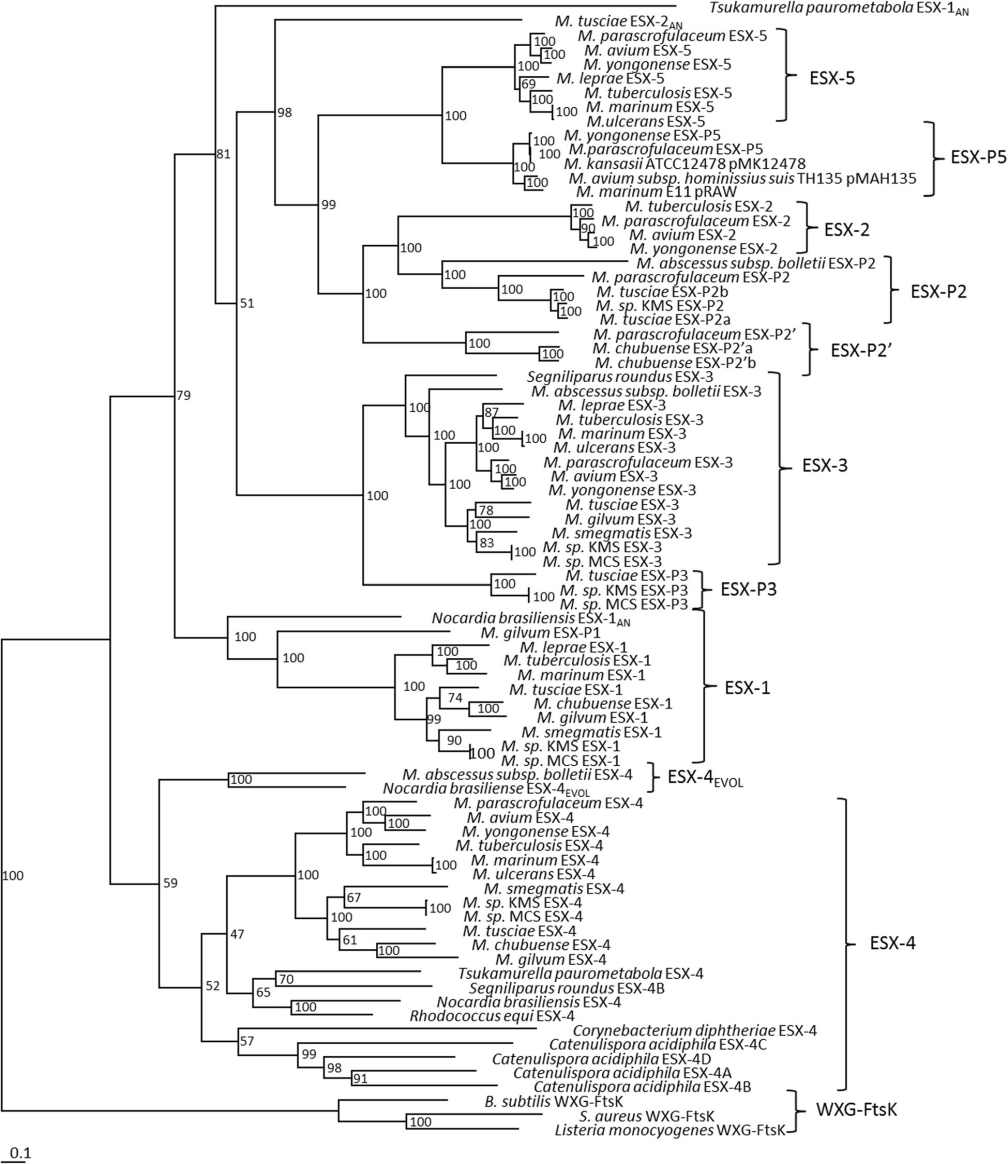
The phylogeny of the ESX gene cluster. Maximum likelihood phylogeny of representative ESX gene clusters describing the evolution of the ESX gene cluster from its WXG-FtsK cluster progenitor. The ESX gene clusters form five groups, ESX-4, ESX-3, ESX-1, ESX-2 and ESX-5. The plasmid located and ancestral ESX gene clusters form subgroups of each genomic ESX gene cluster. The ESX gene clusters have evolved divergently from a single duplication of ESX-4 to ESX-1 and ESX-3 and then ESX-2 and ESX-5. One hundred subsets were generated for bootstrapping resampling of the data

ESX gene clusters were identified from the publicly available genome sequences of 60 actinobacterial species, including 40 mycobacterial species, 11 other species from the order *Corynebacteriales* and 9 species selected from the orders *Pseudonocardiales, Glycomycetales, Micromonosporales, Frankiales, Streptosporangiales, Catenulisporales, Streptomycetales, Propionibacteriales* and *Kineosporiales* (Table 1). Each genome contains between one and seven ESX gene clusters. The components and arrangement of each ESX gene cluster were determined and are represented in Additional file 1 with three WXG-FtsK clusters from *Staphylococcus aureus*, *Listeria monocytogenes* and *Bacillus subtilis*, identified in the literature as precursors of the ESX gene cluster [30, 31]. The concatenated protein sequences of each ESX gene cluster were aligned and used to generate a phylogeny of the ESX gene clusters using maximum likelihood (ML) and distance methods (Fig. 1 and Additional file 2) using the WXG-FtsK clusters of *S. aureus*, *L. monocytogenes* and *B. subtilis* as the outgroup. The topology of the trees generated by ML and distance methods was conserved, depicting 5 distinct clades, each incorporating one of the *M. tuberculosis* H37Rv ESX gene cluster regions 1 to 5.

**Table 1:** WXG-FtsK and ESX gene clusters identified in sequenced mycobacterial and selected actinobacterial species

Species	WXG_FtsK	Genomic ESX						Plasmid ESX				
		4	4ev	3	1	2	5	P1	P2	P3	P2′	P5
*Bacillus subtilis* subsp. *subtilis* str. 168	x											
*Catenulispora acidiphila* DSM43021		xxxx										
*Corynebacterium diphtheriae* NCTC 13129		x										
*Corynebacterium pseudotuberculosis* FRC41		x										
*Frankia alni* ACN14a		x										
*Gordonia bronchialis* DSM 43247		x										
*Janibacter sp.* HTCC2649		x										
*Kribbella flavida* DSM17836		xx										
*Listeria monocytogenes* L312	x											
*M. abscessus*		x		x								
*M. abscessus* subsp. *bolletii* 50594			x	x					x			
*M. africanum* GM041182		x		x	x	x	x					
*M. avium* 104		x		x		x	x					
*M. avium paratuberculosis* K-10		x		x		x	x					
*M. bovis* AF2122/97		x		x	x	x	x					
*M. bovis* BCG Pasteur 1173P2		x		x	x^b^	x	x					
*M. canettii* CIPT140010059		x		x	x	x	x					
*M. chubuense*		x			x						xx	
*M. colombiense* CECT3035^a^		x		x		x	x					
*M. fortuitum* subsp. fortuitum DSM 46621^a^		x	x	x	x							
*M. gilvum* PYR-GCK		x		x	x			x				
*M. indicus pranii* MTCC9506		x		x		x	x					
*M. intracellulare* ATCC13950		x		x		x	x					
*M. kansasii* ATCC12478^a^		x		x	x	x	x					
*M. leprae* TN				x	x		x					
*M. ulcerans* subsp*. liflandii* 128FXT		x		x	x		x					
*M. marinum* M		x		x	x		x					
*M. massiliense* CCUG48898^a^		x		x								
*M. microti* 19422^a^		x		x	x^b^	x	x					
*M. neoaurum* VKM Ac-1815D		x		x	x							
*M. orygis* 112400015		x		x	x	x	x					
*M. parascrofulaceum* BAA-614^a^		x		x		x	x		x		x	x
*M. phlei* RIVM601170^a^		x		x	x							
*M. rhodesiae* NBB3^a^		x		x	x							
*M. smegmatis* mc^2^155		x		x	x							
*M. sp.* JDM601		x		x		x	x					
*M. sp.* JLS		x		x	x							
*M. sp.* KMS		x		x	x				x	x		
*M. sp.* MCS		x		x	x					x		
*M. sp.* MOTT36Y		x		x		x	x					
*M. sp.* Spyr 1		x		x	x							
*M. thermoresistibile* 19527		x		x	x							
*M. tuberculosis* H37Rv		x		x	x	x	x					
*M. tusciae* JS617^a^		x		x	x	x^c^			xx	x		
*M. ulcerans* Agy99		x		x			x					
*M. vaccae* ATCC 25954		x	x	x	x							
*M. vanbaalenii* PYR-1		x		x	x							
*M. xenopi* RIVM700367^a^		x		x		x	x					
*M. yongonense* 05-1390		x		x		x	x					x
*Nocardia brasiliensis* ATCC700358		x	x		x^c^							
*Nocardia cyriacigeorgica* GUH-2		x	x		x^c^							
*Nocardia farcinica* IFM 10152		x	x									
*Rhodococcus equi* 103S		x										
*Rhodococcus erythropolis* PR4		x										
*Rhodococcus opacus* B4		x										
*Staphylococcus aureus* USA300	x											
*Streptomyces coelicolor* A3 (2)		x										
*Saccharopolyspora erythraea* NRRL 2338		xx										
*Stackebrandtia nassauensis* DSM 44728		xxxx										
*Streptosporangium roseum* DSM 43021		x										
*Segniliparus rotundus* DSM 44985			x	x								
*Salinispora tropica* CNB-440		xx										
*Tsukamurella paurometabola* DSM 20162			x		x^c^							

^a^Sequencing projects are incomplete (as of 07/2015)

^b^RD1 deletion within cluster

^c^Ancestral region

ESX gene clusters were identified on plasmids in several mycobacterial species (pMFLV01 in *M. gilvum,* pMKMS01 and pMKMS02 in *M. sp.* KMS, Plasmid01 in *M. sp.* MCS, pMYCCH.01 and pMYCCH.02 in *M. chubuense*, pMYCSM01, pMYCSM02 and pMYCSM03 in *M. smegmatis* JS623, Plasmid 2 in *M. abscessus* sp. *bolletii* and pMyong1 in *M. yongonense*). Four additional mycobacterial plasmid-encoded ESX gene clusters were previously identified by Ummels et al., (2014) [33]. The sequences of three of these, on pRAW from *M. marinum* E11, pMAH135 from *M. avium* subsp. *hominis suis* T135 and pMK12478 from *M. kansasii* ATCC12478, are publicly available and were included in the phylogenetic analyses. The plasmid-encoded ESX clusters group phylogenetically with some of the ESX gene clusters identified on contigs from the incomplete genome sequences of *M. tusciae* and *M. parascrofulaceum* and together form a subclade of each genomic ESX duplication subsequent to ESX-4 (Fig. 1). The *M. parascrofulaceum* and *M. tusciae* sequencing projects are incomplete, therefore it was not possible to conclusively determine whether the ESX gene clusters identified in these species are plasmid or chromosomally located. However, based on synteny and the phylogenetic clustering of these *M. tusciae* and *M. parascrofulaceum* ESX with the plasmid-encoded ESX clusters, these ESX are predicted to be encoded on plasmids, or to originate directly from plasmid DNA. Sequence alignments indicate that each contig containing a predicted plasmid-located ESX cluster shares several conserved segments, or locally collinear blocks (LCBs), with the ESX-containing plasmids from the same subclade (Additional file 3). This is particularly apparent for sequences containing the subclade of ESX-3, which consist almost entirely of four LCBs, and the subclade of ESX-5. This supports the definition of these *M. tusciae* and *M. parascrofulaceum* ESX gene clusters as plasmid ESX gene clusters. The ESX gene clusters on the plasmids and *M. tusciae* and *M. parascrofulaceum* contigs, which form outgroups to ESX-1, -2, -3 and -5, were named ESX-P1, -P2, -P2’, -P3 and -P5, where “P” indicates the plasmid localisation of the ESX (Table 2). ESX-P1, ESX-P2, ESX-P3 and ESX-P5 form outgroups to the genomic ESX with the same numbers and ESX-P2’ branches off prior to ESX-P2. ESX-P1 to -P5 contain all of the core ESX components, including *esp*G and *esp*I and ESX-P1 also incorporates EspH, while EccA is absent from ESX-P2.

**Table 2:** The plasmid-encoded ESX clusters

ESX	Species	Plasmid/contig	Accession number	Size (bp)
P1	*M. gilvum* PYR-GCK	pMFLV01	NC_009339.1	321,253
P2	*M. abscessus* subsp. *bolletii* 50594	plasmid 2	NC_021279.1	97,240
	*M. parascrofulaceum* BAA-614	contig00115	ADNV01000102.1	21,921
	*M. sp* KMS	pMKMS01	NC_008703.1	302,089
	*M. tusciae* JS617	contig 196	NZ_AGJJ01000027.1	108,484
	*M. tusciae* JS617	contig 209	NZ_AGJJ01000007.1	249,244
P2′	*M. chubuense*	pMYCCH.01	NC_018022	615,278
	*M. chubuense*	pMYCCH.02	NC_018023	143,623
	*M. parascrofulaceum* BAA-614	contig00017	ADNV01000015.1	70,331
P3	*M. sp* KMS	pMKMS02	NC_008704.1	216,763
	*M. sp*. MCS	Plasmid1	NC_008147.1	215,075
	*M. tusciae* JS617	contig 224	NZ_AGJJ01000010.1	218,303
P5	*M. avium subsp. hominissuis suis* TH135	pMAH135	AP012556	194,711
	*M. kansasii* ATCC12478	pMK12478	CP006836	144,951
	*M. marinum* E11	pRAW	HG917973	114,229
	*M. parascrofulaceum* BAA-614	contig00109	ADNV01000096.1	47,725
	*M. yongonense* 05-1390	pMyong1	JQ657805	122,976

### ESX-4

Orthologs of the ESX-4 gene cluster were identified in all of the mycolic acid producing species from the genera *Mycobacterium, Gordonia, Nocardia, Rhodococcus* and *Corynebacterium.* ESX-4 gene clusters were also identified in the 9 species from the orders *Pseudonocardiales, Glycomycetales, Micromonosporales, Frankiales, Streptosporangiales, Catenulisporales, Streptomycetales, Propionibacteriales* and *Kineosporiales* which do not have mycolic acids in their cell envelope. These organisms each contain between one and four copies of the ESX-4 gene cluster. Although the arrangement and components of this gene cluster are well conserved amongst the mycobacterial species; insertions, deletions and rearrangements are common amongst the non-Mycolata. The ESX-4 gene cluster contains genes encoding the FtsK/SpoIIIE protein EccC, and two WXG proteins, EsxU and EsxT, which are present in the FtsK-WXG clusters of *S. aureus, L. monocytogenes* and *B. subtilis*. In addition to the WXG-FtsK cluster components, ESX-4 encodes EccD, EccB and MycP, which have been suggested to be involved in a more intricate secretion mechanism to transport proteins into and across the unique and complex outer mycomembrane [34]. However, the presence of the ESX-4 cluster in various non-mycomembrane containing actinobacteria suggests that the secretion system encoded by these gene clusters is not directly involved in mycomembrane translocation. Although the function(s) of ESX-4 have yet to be determined, the presence and maintenance of this gene cluster throughout the mycobacteria and other actinobacteria suggests that it plays an important role in bacterial metabolism. Homologs of the ESX-4 gene cluster components occur in all 5 ESX gene clusters and could represent the proteins required for translocation across the inner membrane. The additional components present in the subsequent ESX duplications may be involved in mycomembrane translocation, be additional substrates, assist in the translocation of additional substrates or facilitate specific mechanisms of those secretion systems.

Phylogenetically associated with the ESX-4 gene cluster is a subgroup of ESX gene clusters which include homologs of the *ecc*A, *ecc*E, *esp*G, *esp*I, *pe* and *ppe* genes, in addition to the ESX-4 components. This cluster was identified in the mycolic acid producing species *N. farcinica, N. brasiliense, N. cyriacigeorgica, T. paurometabola, S. rotundus, M. vaccae, M. fortuitum* and *M. abscessus* subsp. *bolletii.* The arrangement of the genes in this cluster varies between species, but does not resemble any of the *M. tuberculosis* ESX gene clusters. This cluster contains all of the conserved ESX gene cluster components and appears to be an evolutionary intermediate between ESX-4 and the subsequent duplications, and is therefore named ESX-4_EVOL_ (ESX-4 evolved).

### ESX-3

ESX-3 present in all of the studied mycobacteria, with the exception of *M. chubuense*, suggesting that ESX-3 is the first ESX duplication in the mycobacterial genome. ESX-3 contains all of the ESX conserved components *ecc*A to E, *myc*P, *esx* and *pe/ppe* pairs as well as *esp*G. Although essential for in vitro growth of *M. tuberculosis,* ESX-3 is not essential in the fast-growing *M. smegmatis* [20]. ESX-3 is involved in iron homeostasis and uptake via the mycobactin pathway [24] and genetic reduction during evolution of the slow-growers may have eliminated the redundancy of ESX-3. Outside of the mycobacteria, ESX-3 was only identified in *S. rotundus* suggesting that ESX-3 was inserted prior to the divergence of *Segniliparus* and *Mycobacterium* from a common ancestor. The presence of three mycobactin genes in the *S. rotundus* ESX-3 furthermore suggests that the association between ESX-3 and iron homeostasis may be conserved. The ancestral mycobacteria *M. abscessus*, *M. abscessus* subsp. *bolletii* and *M. massiliense* contain only ESX-4 (or ESX-4evol) and ESX-3.

### ESX-1

ESX-1 is present in all of the other fast-growing mycobacteria; *M. thermoresistibile*, *M. smegmatis* mc^2^155*, M. neoaurum, M. fortuitum, M. vanbaalenii, M. gilvum, M. sp.* Spyr1, M. vaccae *M. rhodesiae, M. phlei*, *M. sp*. JLS*, M. sp.* KMS and *M. sp.* MCS; but has been deleted from the genomes of various slow-growing mycobacteria (*M. avium, M. avium paratuberculosis, M. colombiense, M. intracellulare, M. parascrofulaceum, M. ulcerans, M. xenopi, M. indicus pranii, M. sp.* MOTT36Y and *M. sp.* JDM601), with partial deletions (Region of Deletion 1, RD1) in *M. bovis* BCG and *M. microti*. ESX-1 contains both *esp*G and *esp*I, and in most cases *ecc*E and *myc*P have been inverted along with the insertion of several additional genes. ESX-1 has been implicated in virulence, and its deletion in attenuation of the pathogenic mycobacteria [8, 9]. However, its presence throughout most of the mycobacteria, including non-pathogenic and saprophytic fast-growing organisms, suggests that the primary function of this gene cluster is not virulence, and that the virulence-associated function has evolved more recently in pathogenic organisms.

An additional gene cluster, identified in the non-mycobacterial actinomycetes *N. brasiliense and N. cyriacigeorgica* contains all of the components of ESX-4_EVOL_, but has an operonic arrangement similar to the *M. tuberculosis* ESX gene clusters. This cluster forms a subgroup just outside of the mycobacterial ESX-1 clade and is therefore named ESX-1_AN_ (ancestral ESX-1). An ESX gene cluster with similar arrangements was identified in *T. paurometabola*, but has undergone a transposition event which has resulted in the disruption of *ecc*C and deletion of *ecc*B. Phylogenetic clustering of this region is not consistent between algorithms and this region is also predicted to be an ESX-1_AN_ cluster, based on synteny.

### ESX-2 and ESX-5

ESX-2 and ESX-5 occur only in the slow-growing mycobacteria. ESX-2 contains all of the conserved ESX components including *esp*G and *esp*I in an operonic structure, while ESX-5 contains only *esp*G, but has multiple copies of *pe* and *ppe*, and the insertion of a ferredoxin and a *cyp*143 gene. The function(s) of ESX-2 have not been elucidated, and although its duplication correlates evolutionarily with both the slow-growing and pathogenic phenotypes, it has been lost from some of these species (*M. leprae, M. marinum, M. ulcerans* subsp. *liflandii* and *M. ulcerans*). ESX-5 is the only ESX gene cluster present in all of the slow-growers but absent in all of the fast-growers, and may be the ESX gene cluster most involved in pathogenicity and the slow-growing phenotype [35]. Deletion of this region, however, does not directly increase the growth rate of *M. marinum* or *M. tuberculosis* [18, 36]*.* ESX-5 has been implicated in immune evasion and in the secretion of the PE and PPE proteins [36, 37]. Only ESX-5 contains multiple copies of the *pe* and *ppe* genes, the numbers of which vary between species, and its evolution is predicted to have preceded the expansion of these gene families [37].

*M. tusciae* contains an ESX cluster, ESX-2_AN_ (ancestral ESX-2), which contains all of the ESX-2 components and precedes both the ESX-2 and ESX-5 clades, as well as ESX-P2’, -P2 and -P5 gene clusters. *M. tusciae* is a slow-growing mycobacterium which, based on 16S rDNA sequencing, clusters with the fast-growing mycobacteria and is most closely related to the fast-growing mycobacteria *M. farcinogenes, M. komossense* and *M. aichiense* [38]*.* The correlation between the presence of an ESX-2/5-like cluster and a slow growth-rate might imply that *M. tusciae* is an evolutionary intermediate between the fast- and slow-growing mycobacteria. The mycolic acid composition of the cell membrane of *M. tusciae* most closely resembles that of the *M. avium* complex and *M. parascofulaceum* [38] suggesting that the different ESX secretion systems may have evolved with changes in the mycomembrane structure; as reflected in the role of ESX-5 in maintaining selective mycomembrane permeability in the slow growing pathogenic *M. tuberculosis* and *M. marinum* species [19]. Investigation of the potential association between these two ESX clusters, mycomembrane structure and growth rate may provide important information regarding the evolution of the often pathogenic, slow-growing mycobacteria.

### Plasmid-mediated ESX evolution

**Fig. 2:**
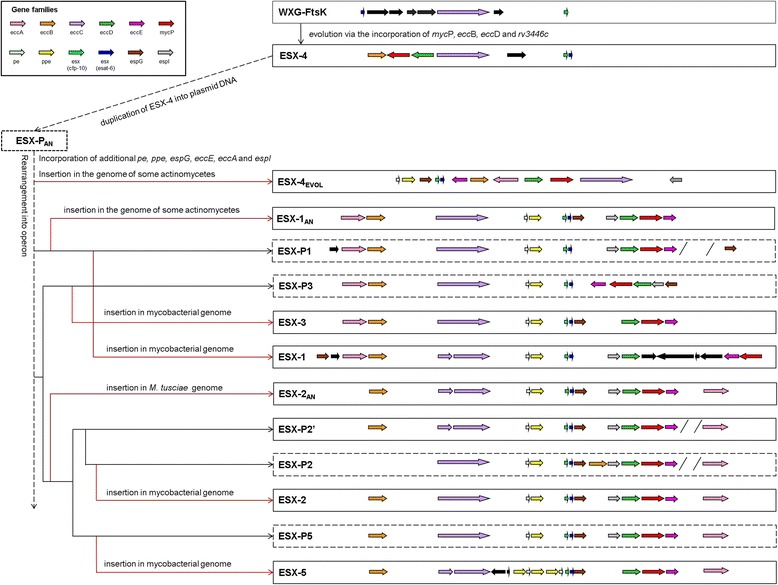
Model of ESX evolution based on plasmid-mediated duplication and evolution. The ancestral ESX-4 gene cluster evolved from the WXG-FtsK cluster via the incorporation of additional genes, *eccB, eccD, mycP* and *rv3446c*. ESX-4 was duplicated into plasmid DNA, into which additional ESX genes, *eccA, eccE, espI, espG, pe* and *ppe*, were incorporated. The plasmid ancestor (ESX-P_AN_) was reinserted into the genomes of various Mycolata, generating ESX-4_EVOL_. Continuous evolution generated the operonic structure of the plasmid ESX gene cluster. Divergent evolution of the plasmid ESX generated several plasmid ESX (ESX-P1, -P3, -P2’, -P2 and -P5) which were inserted into the mycobacterial genome to generate ESX-3, ESX-1, ESX-2 and ESX-5. An earlier version of ESX-P1 was inserted into the genomes of some actinomycetes as ESX-1_AN_ and a precursor of ESX-P2’ was inserted into the *M. tusciae* genome as ESX-2_AN._ Red arrows represent genome insertions

The duplication and evolution of the ESX gene clusters and their secretion systems have clearly impacted on the evolution, diversity and success of the mycobacteria. The identification of ESX gene clusters on several plasmids within the mycobacteria, and their phylogenetic association with each of the genomic ESX gene clusters, provides novel insight into the mechanism of ESX evolution suggesting that the duplication and diversification of these clusters was plasmid-mediated. The presence of multiple plasmid copies within a single organism facilitates diversification by allowing the coevolution of various ESX clusters simultaneously. The plasmid localisation furthermore facilitates the loss of deleterious effects, while the incorporation of beneficial plasmid DNA into the genome allows permanent retention and might be selected for. We propose a model for the plasmid-mediated duplication and evolution of the ESX gene clusters (Fig. 2).

Based on this model, the FtsK-WXG cluster present in the *Firmicutes* evolved to form the ESX-4 gene cluster, through the incorporation of *ecc*B, *ecc*D and *myc*P, during the evolution of the actinobacteria; resulting in the presence of ESX-4 in the genomes of various actinobacterial species. A copy of ESX-4 has been incorporated into plasmid DNA after the divergence of the genera *Corynebacterium* and *Rhodococcus*. The additional ESX components, *eccA, eccE, espG, espI, pe* and *ppe*, were incorporated into this plasmid-located cluster (ESX-P_AN_), which was subsequently incorporated into the genomes of some species, including *Nocardia* ssp., *T. paurometabola*, *S. rotundus* and *M. abscessus* subsp. *bolletii*, as ESX-4_EVOL._ The variation in the arrangement and sequences of the genes in these clusters may represent independent insertions at different evolutionary time points. The presence of both ESX-4 and ESX-4_EVOL_ in some species implies that ESX-4_EVOL_ is a duplication of the ESX-4 cluster, and has not evolved directly from it. ESX-1, -2, -3, and 5 have evolved from a single duplication of ESX-4. The presence of all of the conserved ESX components in ESX-4_EVOL_ suggests that it evolved from the same progenitor and that ESX-4_EVOL_ is an intermediate between ESX-4 and ESX-1, -2, -3 and -5. Continual evolution of this plasmid ESX gene cluster generated the operonic structure characteristic of the mycobacterial ESX gene cluster duplications. Plasmid precursors of the four duplications, ESX-P1, -P3, -P2’, -P2 and -P5, have evolved simultaneously by divergence of the common plasmid ancestor, after which genome insertions generated the genomic ESX-1, -2, -3 and -5 clusters.

It appears furthermore, that these plasmids may be able to transfer between mycobacterial species. The pRAW, pMyong1, pMK12478 and pMAH135 plasmids, which contain ESX-P5, were also shown to contain components of a Type-IV secretions system and a *tra*A/relaxase gene; which are required for conjugation of the plasmid between some slow-growing mycobacterial species [33].

### ESX-associated evolution of the mycobacteria

**Fig. 3:**
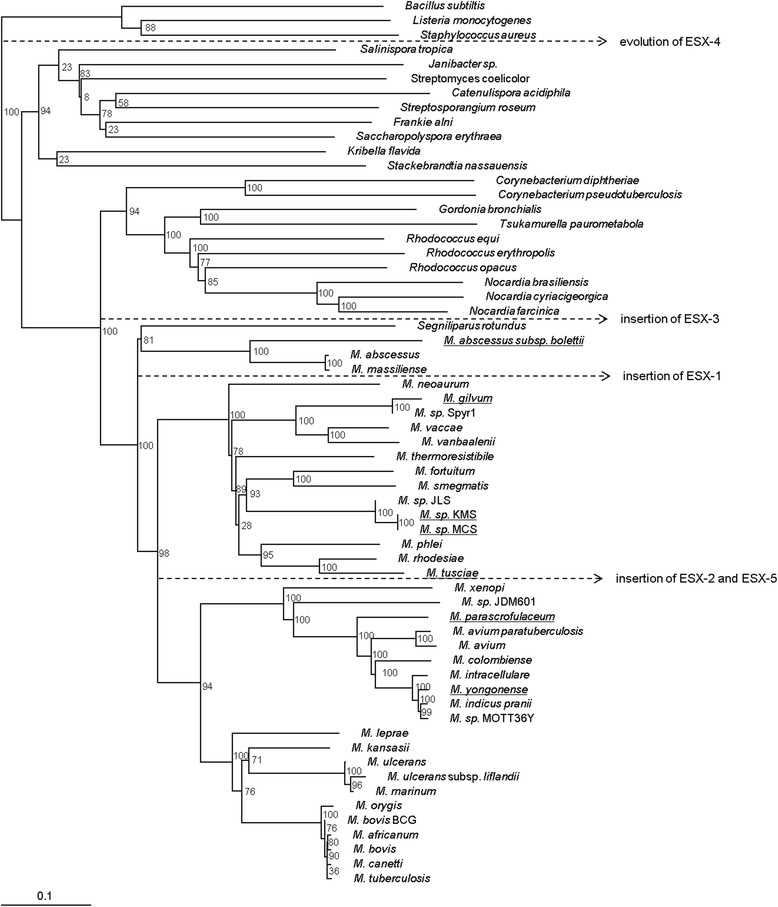
The phylogeny of the mycobacteria based on ESX duplication and evolution. Maximum likelihood phylogeny describing the evolution of the mycobacteria based on the concatenated ESX gene cluster amino acid sequences from each species. ESX duplication and deletion events influenced the evolution and diversification of the mycobacteria as described in the text. Species which contain plasmid ESX gene clusters are underlined. One thousand subsets were generated for bootstrapping resampling of the data

A phylogenetic analysis of the mycobacteria and related actinomycetes based on their ESX gene clusters was done using the concatenated protein sequences of all of the ESX gene clusters of each species (Fig. 3). The *Mycolata* have evolved from a single gram-positive monoderm ancestor into two groups, those which contain only ESX-4, ESX-4_EVOL_ and ESX-1_AN_, the non-mycobacterial actinomycetes; and those which also contain an ESX-3 gene cluster, which with the exception of *S. rotundus*, consist of the mycobacteria. *S. rotundus* contains ESX-4_EVOL_ and ESX-3, while all of the mycobacteria contain at least ESX-4 and ESX-3, with the exception of *M. leprae* which has lost ESX-4. ESX-1 was incorporated in the mycobacterial genome after the divergence of *M. abscessus* and *M. massiliense*, and is present in all of the other fast-growing mycobacteria. However, an ESX-1-like cluster (ESX-1_AN_) is also present in some *Nocardia* ssp.. ESX-1_AN_ predates ESX-P1 and was likely incorporated into the genome from an earlier form of ESX-P1, after the divergence of the mycobacteria. The presence of ESX-1_AN_ in the absence of ESX-3 in some *Nocardia* species, and the presence of ESX-3 in the absence of ESX-1 in *M. abscessus ssp., M. massiliense* and *S. rotundus* suggests that these plasmid clusters evolved simultaneously in an ancestral species, and were inserted into the genomes of the different organisms at different times. The role of ESX-1 in conjugal DNA transfer in *M. smegmatis* [25, 26] may be linked to its origin in plasmid DNA, where it may have facilitated the transfer of the plasmid during cell division.

The presence of ESX-2 and ESX-5 marks the emergence of the slow-growing mycobacteria. ESX-2 and ESX-5 evolved from a common ancestral plasmid-ESX, which diverged to produce ESX-P2’ and ESX-P2; and -P5. ESX-2 and ESX-5 were integrated into the mycobacterial genome with the divergence of the slow-growing mycobacteria, however the presence of ESX-2_AN_, ESX-P2’ and ESX-P2 in various fast-growing mycobacteria attests to the presence of these precursors earlier in mycobacterial evolution. The *M. avium* complex can be distinguished by the transposition of EccB_2_ and EccC_2_. ESX-2 was deleted from a precursor of *M. ulcerans, M. ulcerans* subsp*. liflandii* and *M. marinum.* ESX-1 was deleted from the genomes of slow-growing mycobacteria on numerous occasions. *M. kansasii* and the *M. tuberculosis* complex have retained all five ESX gene clusters, with the exception of *M. bovis* BCG and *M. microti* (not shown), which contain the previously described RD1 deletions in ESX-1 [3, 4, 39–41]. *M. leprae,* which has undergone extensive gene reduction, has retained only ESX-3, -1 and -5 and *M. ulcerans* has retained only ESX-4, -3 and -5.

## Conclusion

The distinctive cell envelope of mycobacteria, characterised by the highly impermeable outer mycomembrane peptidoglycan-arabinogalactan-mycolic acid matrix [6], provides a protective barrier against extracellular stresses, but also presents an obstacle to the export of proteins and acquisition of nutrients. Although mycobacteria possess both Sec and Tat secretion systems, which translocate proteins across the inner membrane, the ESX, or Type VII, secretion systems are the first mechanism proposed for the secretion of proteins into and across the mycomembrane. This study explored the evolution of the mycobacterial Type VII ESX gene clusters from the WXG-FtsK cluster in *S. aureus*, *L. monocytogenes* and *B. subtilis* to the 5 ESX gene clusters in *M. tuberculosis.* The ancestral ESX gene cluster (ESX-4) was identified in several non-mycomembrane producing actinobacteria as well as the non-mycobacterial *Corynebacteriales.* Between two and seven ESX gene clusters were identified in each mycobacterial species. A novel ESX gene cluster, ESX-4_EVOL_, was identified in some non-mycobacterial myco-membrane containing actinomycetes and *M. abscessus* subsp. *bolletii.* ESX-4_EVOL_ contains all of the conserved components of the ESX and appears to be a precursor of the mycobacterial ESX duplications. Plasmid-encoded precursor ESX were identified for each of the genomic ESX-3, -1, -2 and -5 gene clusters and a novel plasmid-mediated mechanism of ESX duplication and evolution proposed. The presence and absence of the ESX gene clusters in the mycobacteria redefines the order of duplication of the ESX gene clusters in the mycobacteria as ESX-4, ESX-3, ESX-1 and then ESX-2 and ESX-5. The influence of the various ESX gene clusters on vital biological and virulence-related functions has clearly influenced the diversification and success of the various mycobacteria, and their evolution from the non-pathogenic fast-growing saprophytic to the slow-growing pathogenic organisms.

## Methods

### Genome sequence data

All protein and DNA sequence information was obtained from publicly available finished and unfinished genome sequencing information. The genomes of 40 mycobacterial species, 11 other species from the order *Corynebacteriale,* nine species selected from the orders *Pseudonocardiales, Glycomycetales, Frankiales, Micromonosporales, Streptosporangiales, Catenulisporales, Streptomycetales, Propionibacteriales* and *Kineosporiales* and 3 gram-positive monoderm species containing WXG-FtsK clusters (Table 1), were analysed.

### Comparative genomic analyses

The *M. tuberculosis* H37Rv ESX protein sequences of interest were used as templates to identify orthologous ESX protein and gene sequences. Blast similarity searches, blastn, tblastn and blastp [42], were done using NCBI Blast and the genome sequence databases listed in Additional file 4. Adjacent genomic regions were searched for additional ESX genes to determine clustering and arrangement of genes; for unfinished genomes in contig format this was not always possible and gene cluster arrangement was assumed based on sequence identity and anticipated arrangement. Large intergenic regions were searched for gene insertions using blastx analyses [43].

### Phylogenetic analyses

Annotated protein sequences were obtained from the protein sequence databases. The protein sequences of conserved components of each ESX gene cluster (EccA, EccB, EccC, EccD, EccE, PE(s), PPE(s), Esx (CFP-10-like), Esx (ESAT-6-like), EspG, EspI, MycP, Rv3446c, EspH, EspJ, EspK, EspL, EspB, Cyp143 and Ferredoxin) were concatenated. Multiple sequence alignments of all concatenated ESX gene cluster protein sequences were done with Clustal W 2.0 [44, 45] using the Bioedit Sequence Alignment Editor version 7.1.3.0 [46]. Similarly, multiple sequence alignments of a single sequence composed of all of the combined ESX gene cluster protein sequences, from each species, were done. Phylogenetic trees were determined by distance and maximum likelihood analyses using SeaView Version 4.4.2 [47]. Distance analysis was done using the observed neighbour-joining method with 10000 bootstrap replicates. Maximum likelihood phylogenies were generated using PhyML [48] with the JTT (Jones Taylor Thornton) algorithm [49], using model-given amino acid equilibrium frequencies, specifying no invariable sites and no across site variation. Nearest-neighbor interchange tree searching operations were used with a BioNJ starting tree. The WXG-FtsK cluster sequences from *S. aureus, L. monocytogenes* and *B. subtilis* were defined as the outgroup. The *M. microti* ESX clusters were omitted from the phylogenetic analyses as protein annotations were not available.

### Plasmid and contig sequence alignments

Plasmid and contig sequences were obtained from the NCBI (Additional file 4) and alignments of the plasmid and contig sequences containing each subgroup of ESX gene cluster were done using the progressiveMauve algorithm of the Mauve 2.3.1 Genome Alignment Visualisation software [50].

## Availability of supporting data

All supporting data are included as Additional files 1, 2, 3 and 4.

## Additional files

Additional file 1: The ESX gene clusters of sequenced mycobacteria and selected actinobacterial species. (DOCX 3463 kb) Additional file 2: Neighbour joining phylogeny of the ESX gene clusters using 10000 bootstrap replicates. (PNG 33 kb) Additional file 3: Mauve sequence alignments of ESX-P containing plasmids and contigs which are predicted to be plasmids. Additional file 4: Analysed genomes. (XLSX 20 kb)
