# Supplementary material for: The plasmid-mediated evolution of the mycobacterial ESX (Type VII) secretion systems
Source: BMC Evol Biol. 2016 Mar 15;16:62. doi: 10.1186/s12862-016-0631-2 (PMC4791881; doi:10.1186/s12862-016-0631-2)
Supplement: Additional file 1: — The ESX gene clusters of sequenced mycobacteria and selected actinobacterial species. (DOCX 3463 kb) [file 12862_2016_631_MOESM1_ESM.docx]

**Additional file 1. The ESX gene clusters of sequenced mycobacteria and selected actinobacterial species.** The WXG-FtsK gene clusters of *S. aureus*, *L. monocytogenes* and *B. subtilis* are as described in the literature. Gene families are represented as coloured arrows as indicated in the legend. ESX gene clusters identified, or predicted to be, on plasmids are named ESX-P1 to -P5. Pseudogenes are indicated as striped arrows. Insertions are indicated. Large insertions or genome rearrangements resulting in genes occurring elsewhere in the genome from the rest of the gene cluster are indicated as transpositions. Genes which are not annotated but for which translated nucleotide results were obtained using tblastn are indicated. The genome sequence of *M. microti* has not been annotated; therefore no gene names are given.

**Gene families**

*
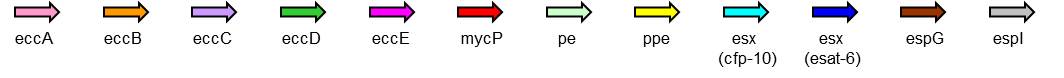
*

**
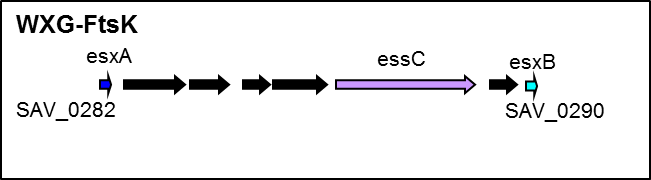
**

*Staphylococcus aureus*

**
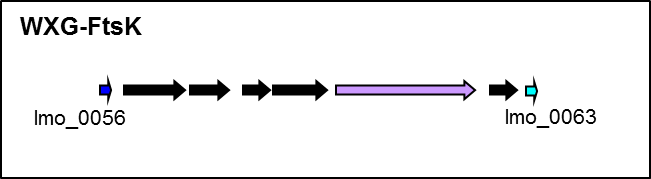
**

*Listeria monocytogenes*

*
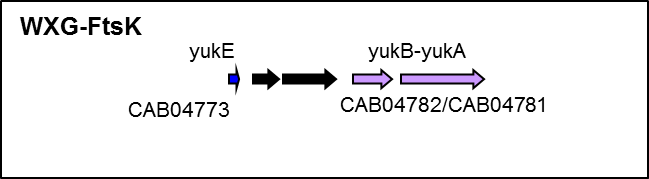
*

*Bacillus subtilis*

*
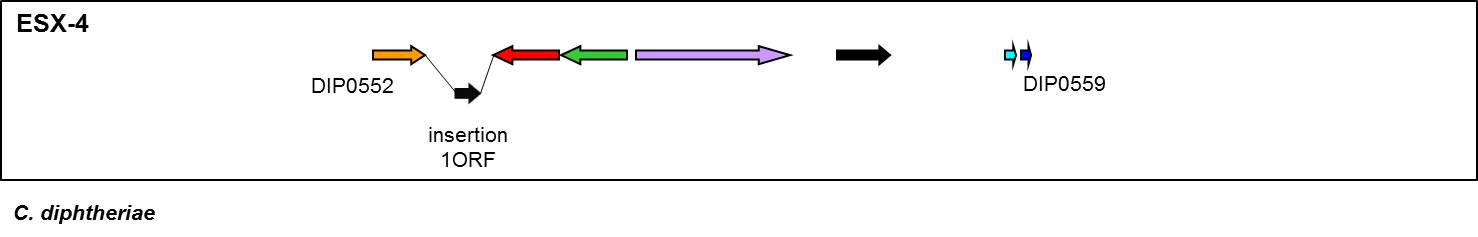
Corynebacterium diphtheriae*

*
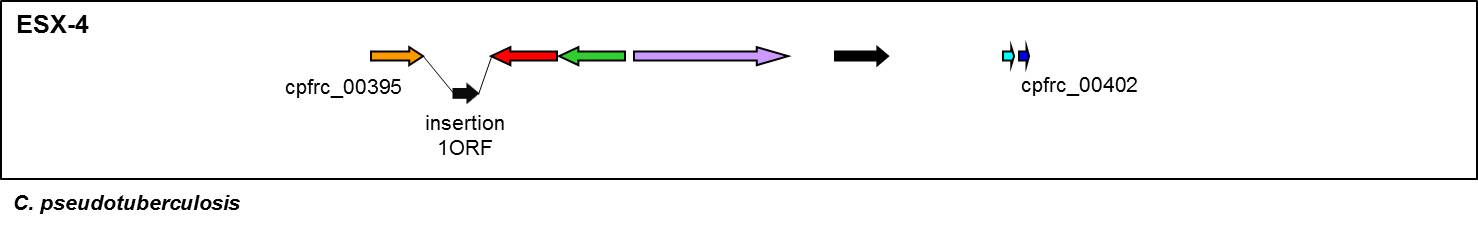
Corynebacterium pseudotuberculosis*

*
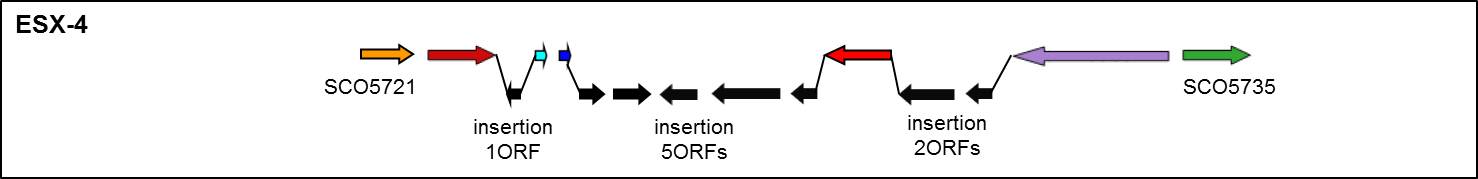
Streptomyces coelicolor*

*
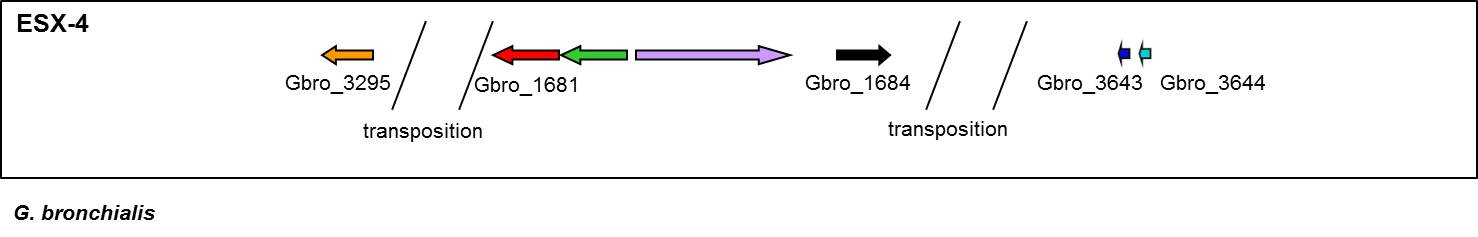
Gordonia bronchialis*

*
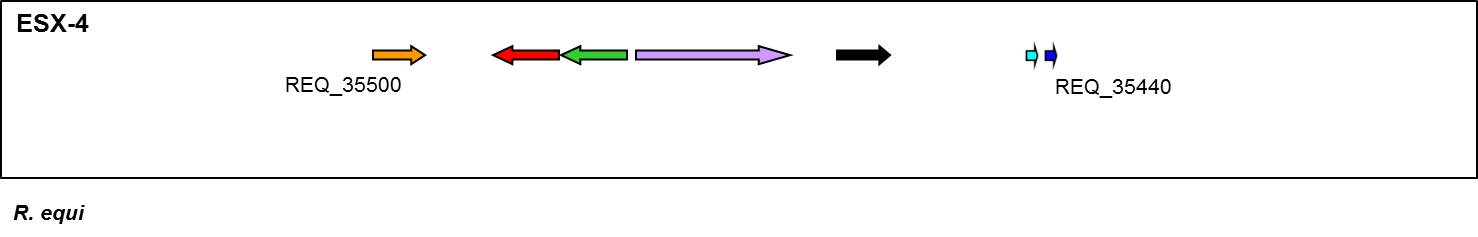
Rhodococcus equi*

*
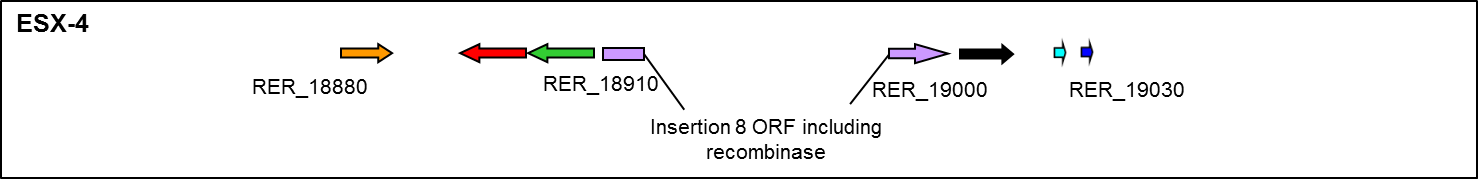
Rhodococcus erythropolis*

*
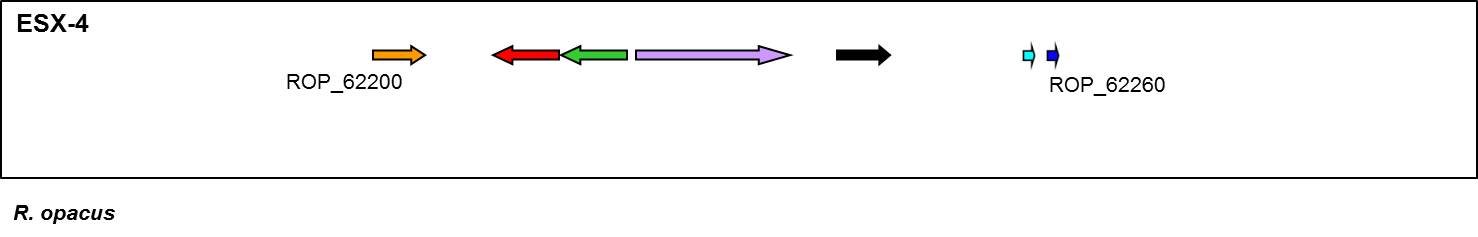
Rhodococcus opacus*

*
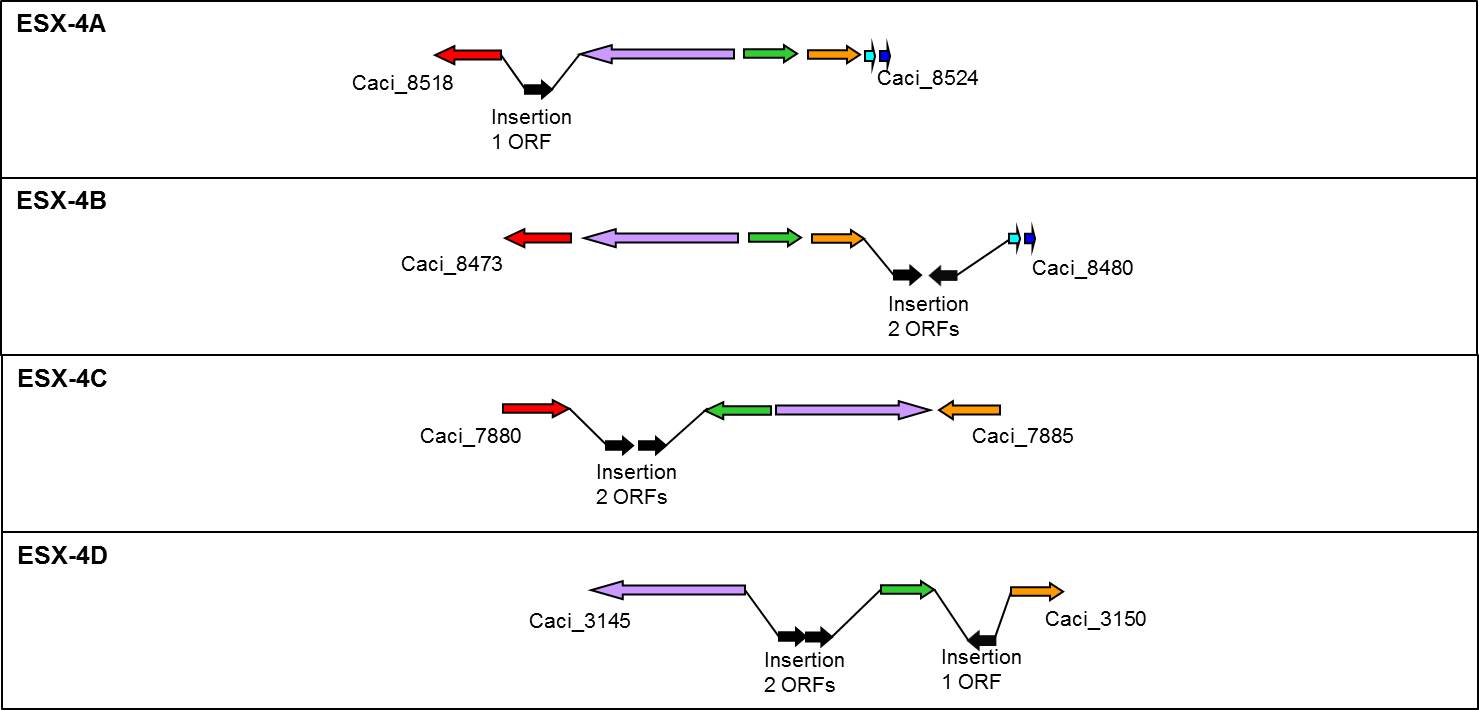
Catenulispora acidiphila*

*
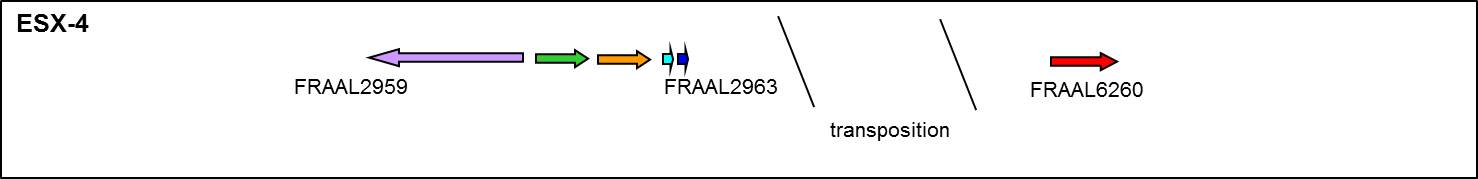
Frankia alni*

*
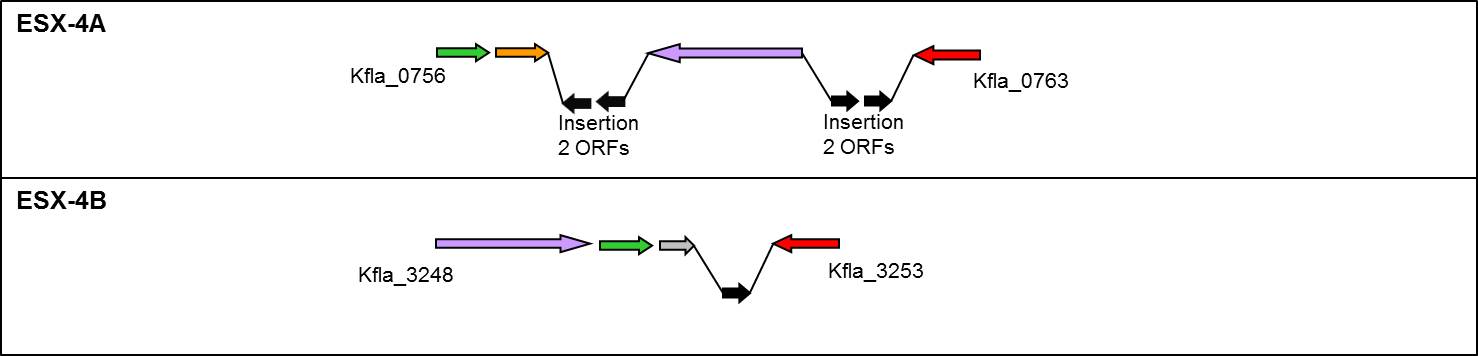
Kribbella flavida*

*
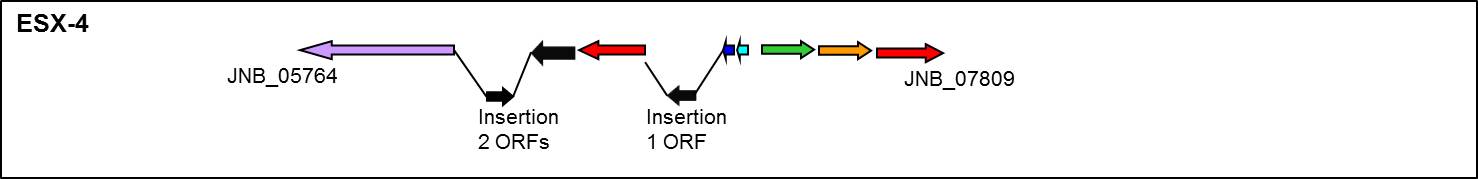
Janibacter sp.* HTCC2649

*
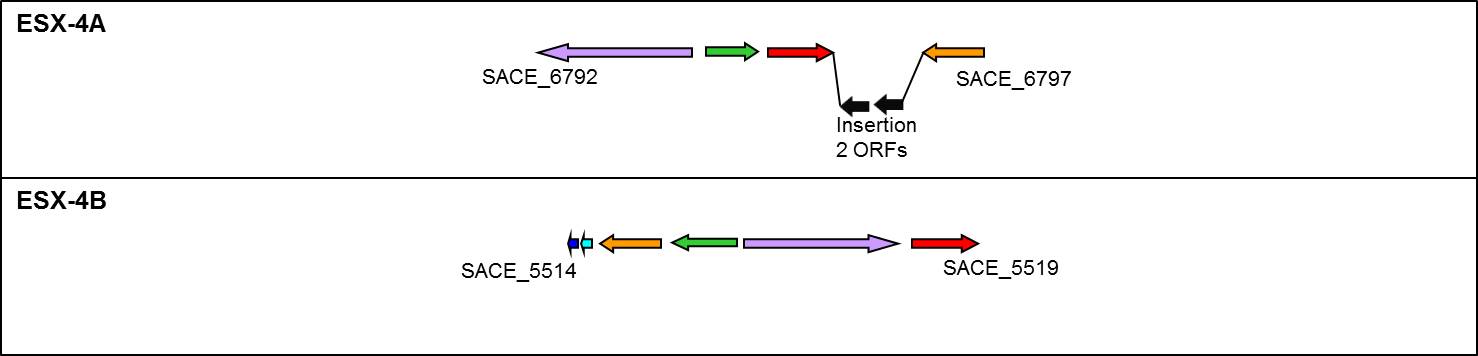
Saccharopolyspora erythraea*

*
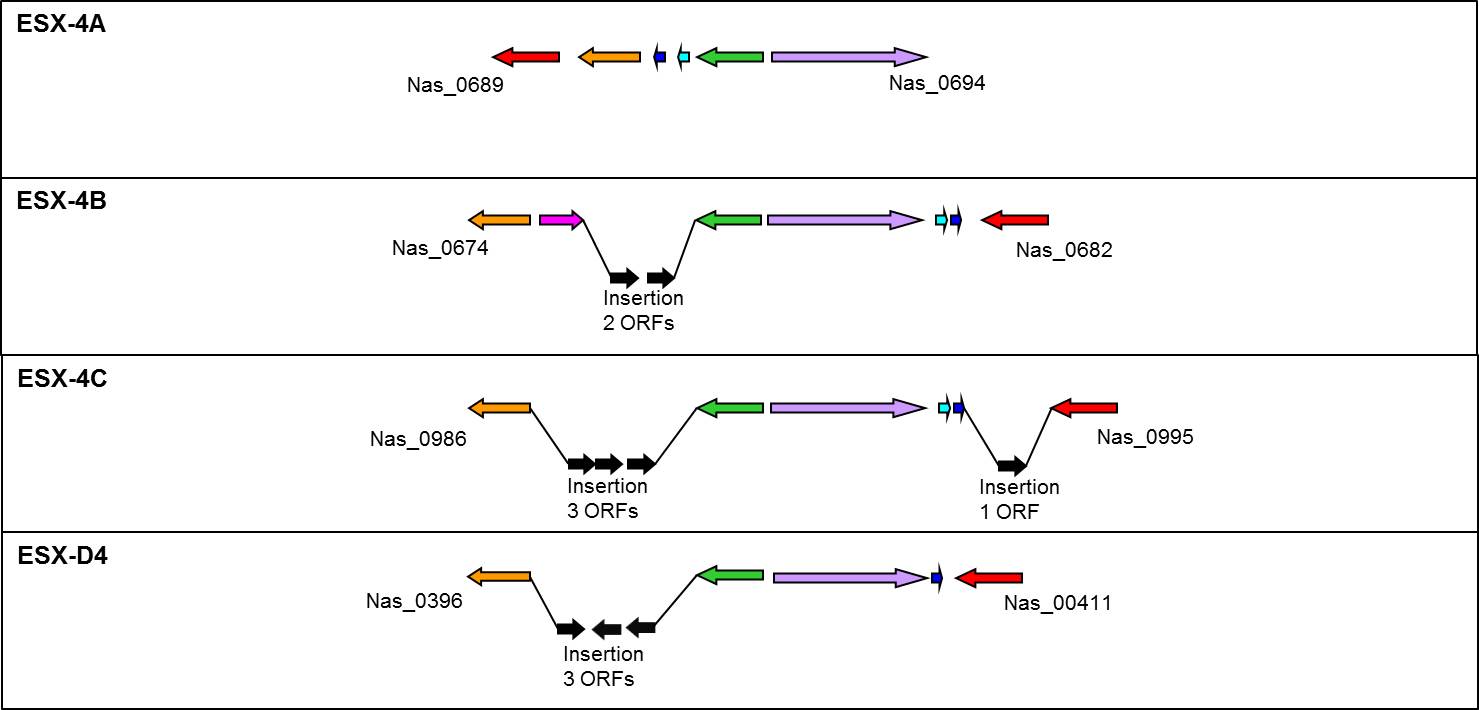
Stackebrandtia nassauensis*

*
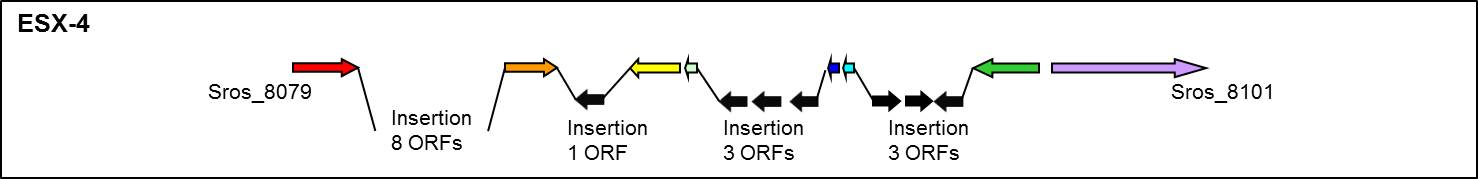
Streptosporangium roseum*

*
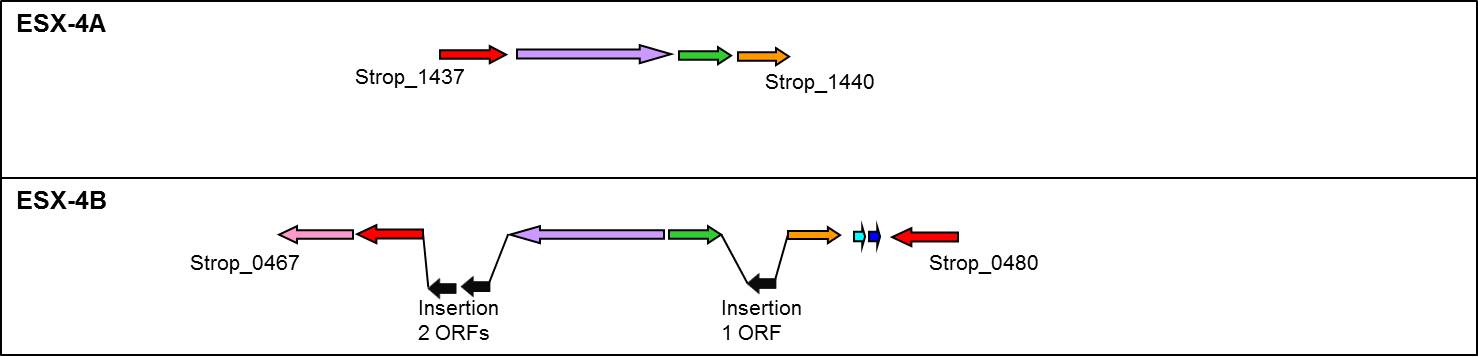
Salinispora tropica*

*
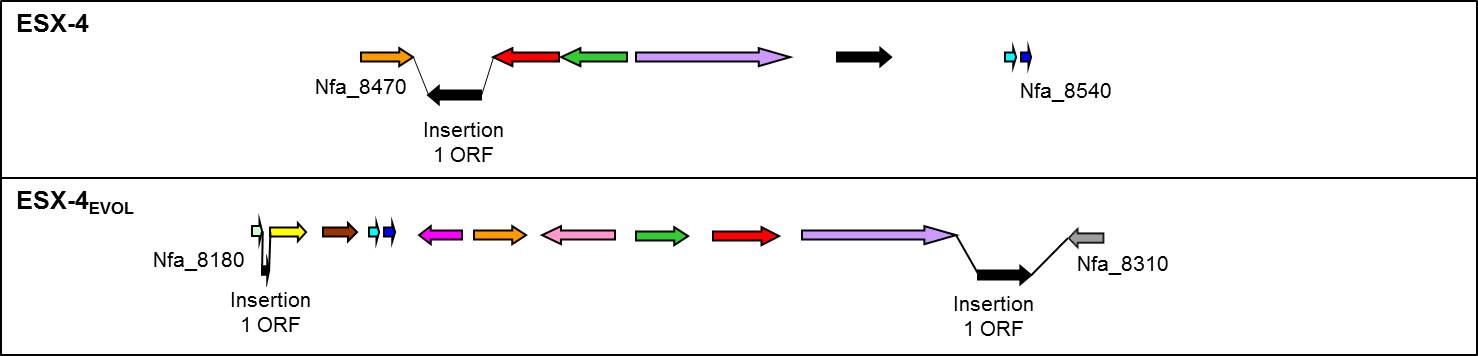
*

*Nocardia farcinica*

*
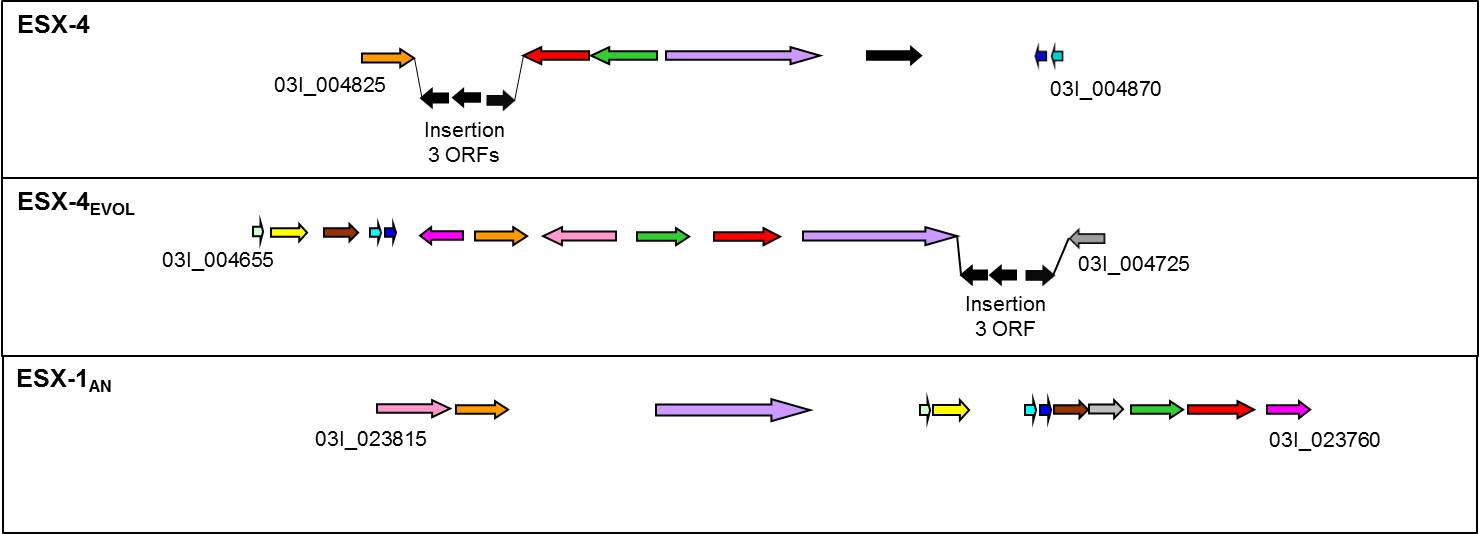
*

*Nocardia brasiliensis*

*
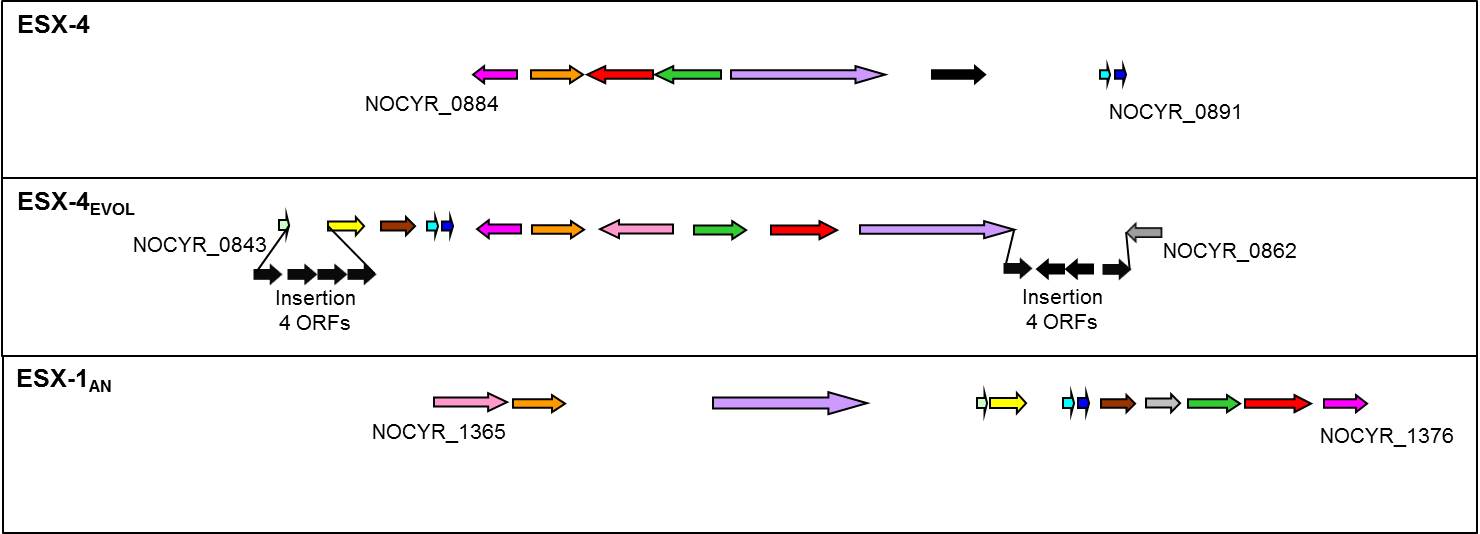
*

*Nocardia cyriacigeorgica*

*
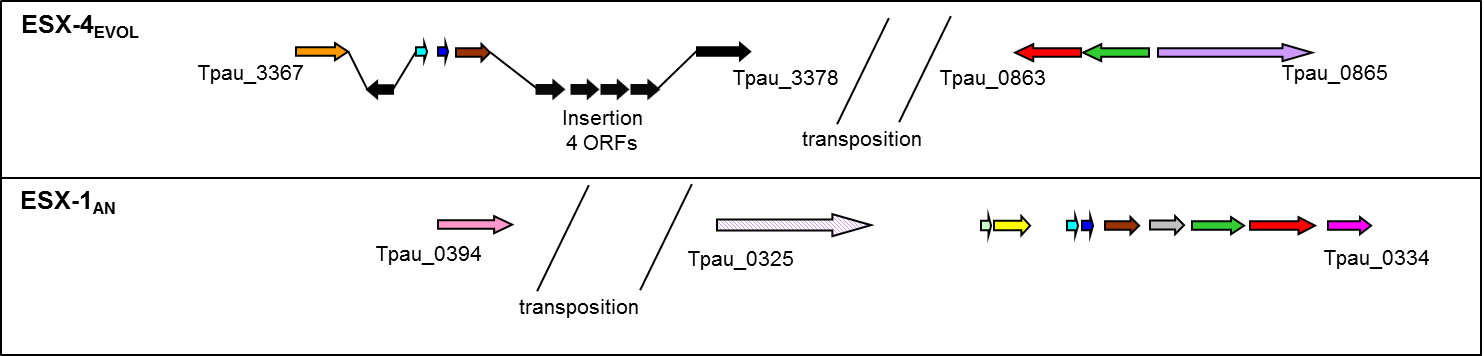
*

*Tsukamurella paurometabola*

*
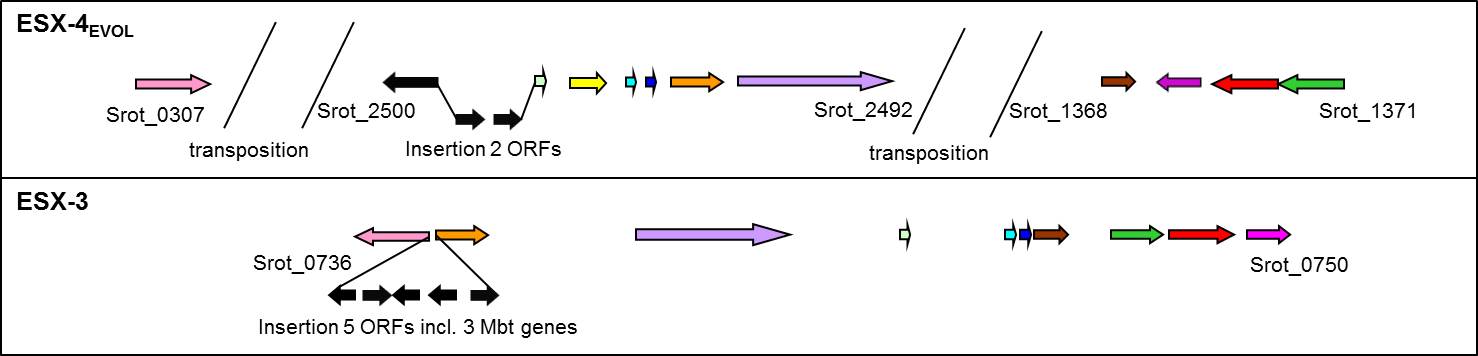
Segniliparus rotundus*

*
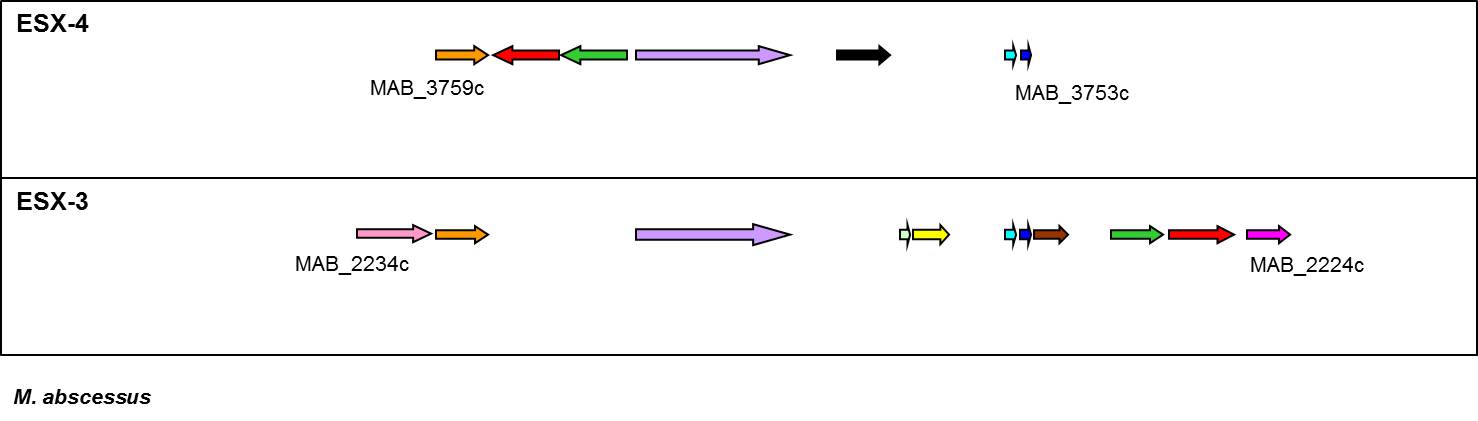
M. abscessus*

*
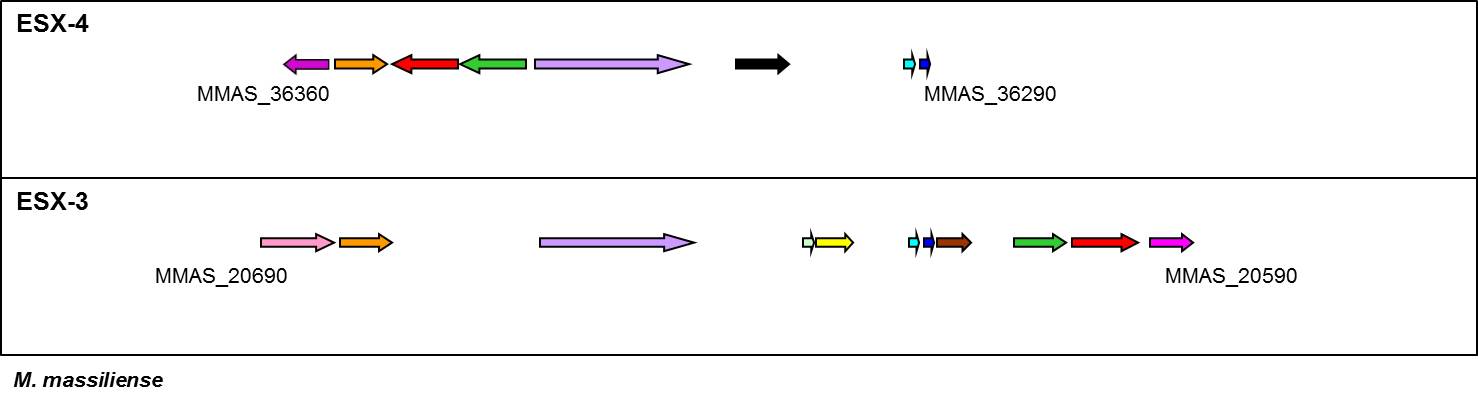
M. massiliense*

*
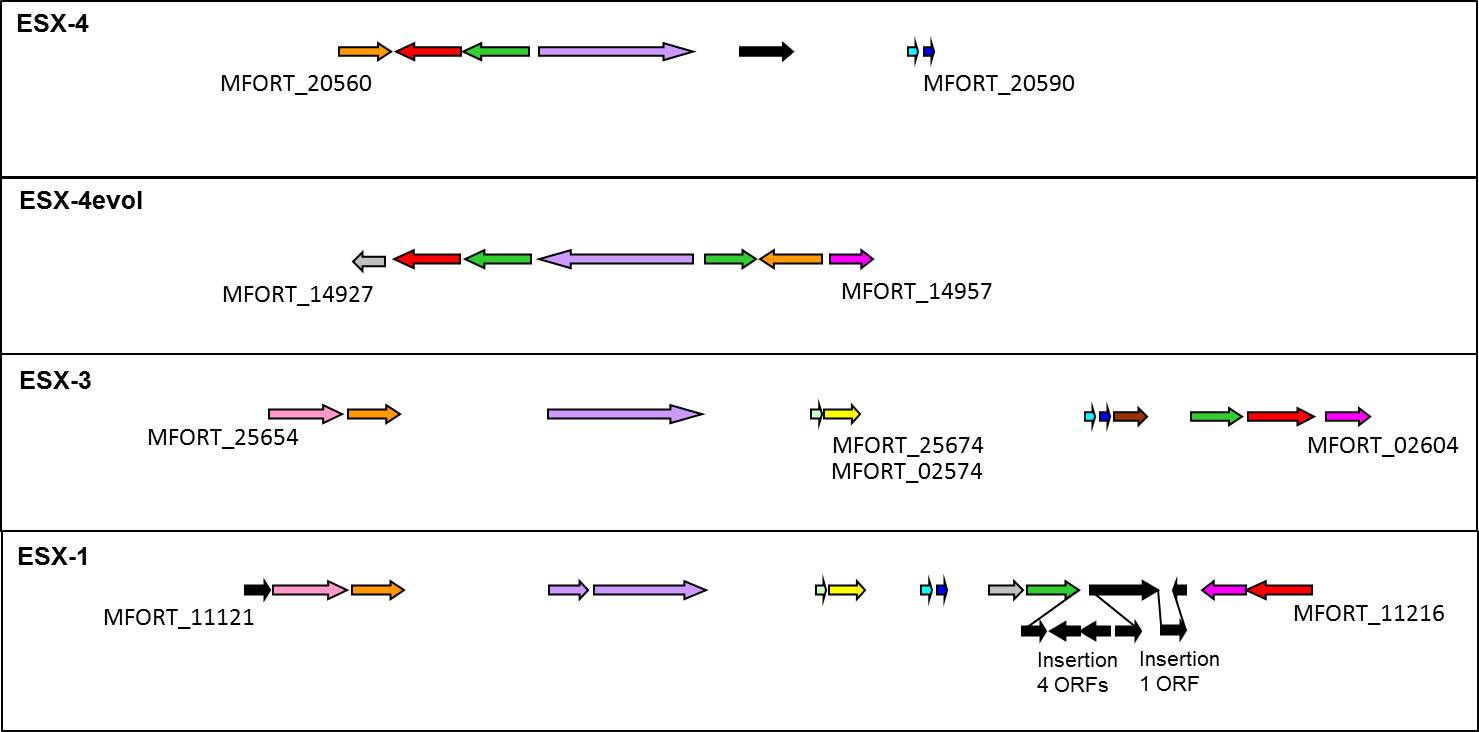
M. fortuitum*

*
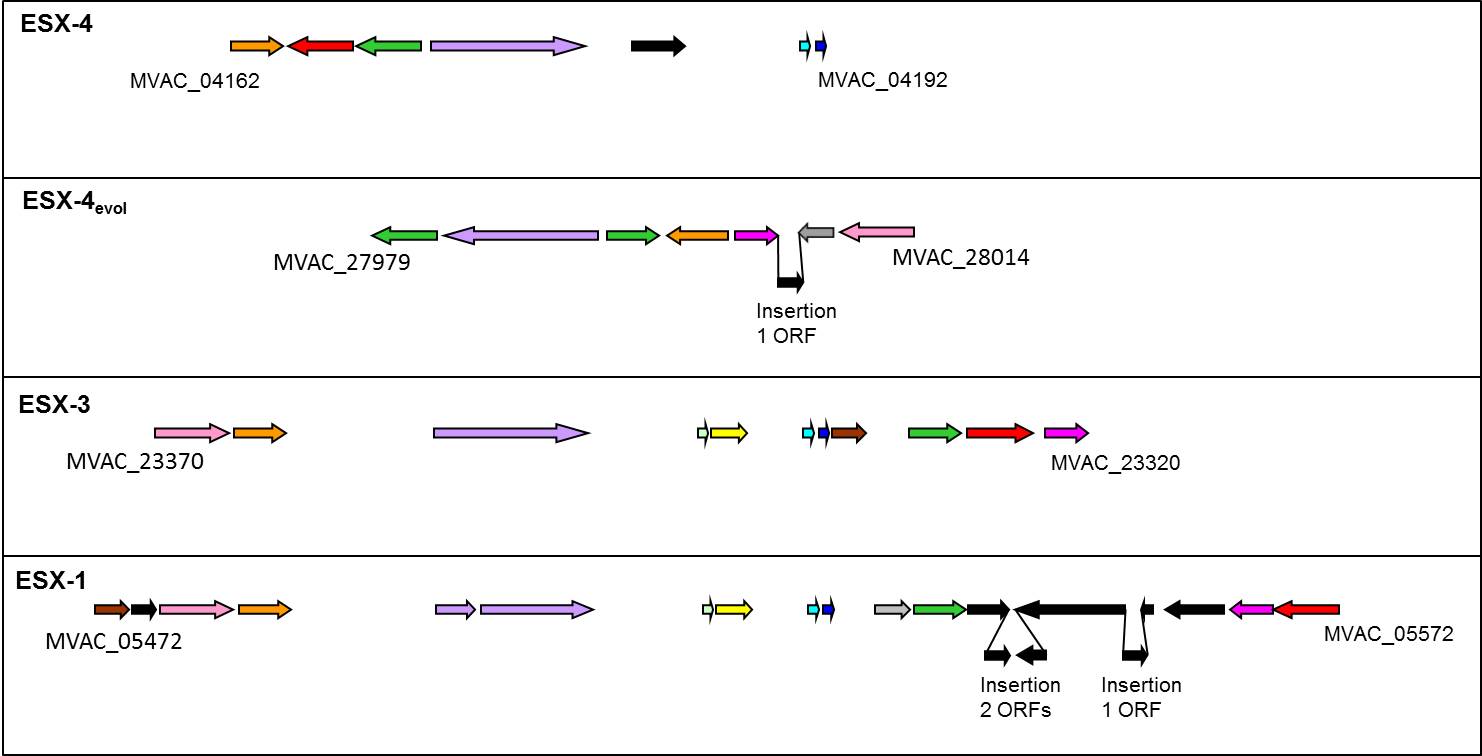
M. vaccae*

*
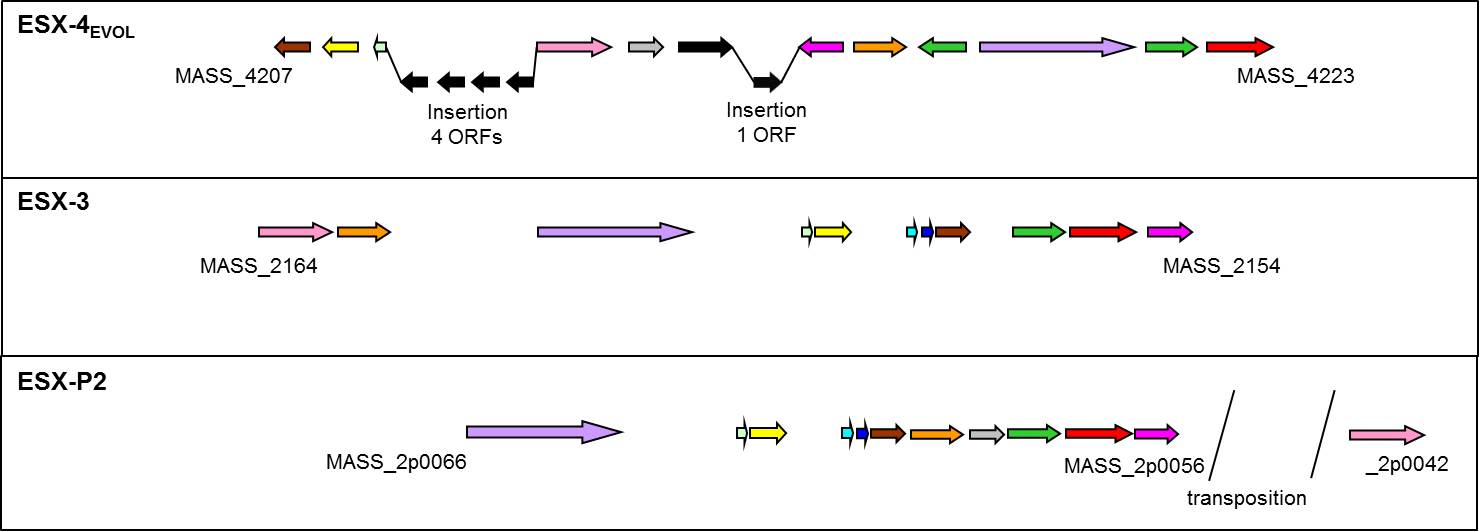
*

*M. abscessus* subsp. *bolletii*

*
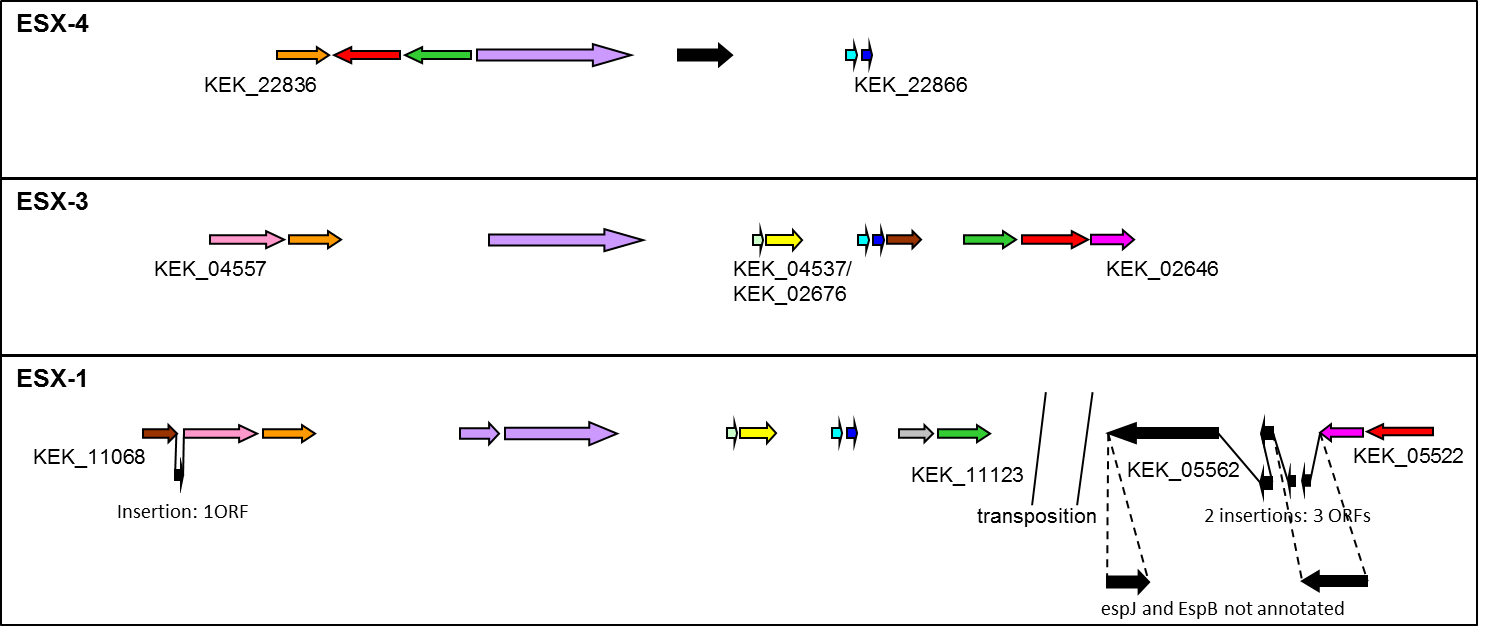
M. thermoresistibile*

*
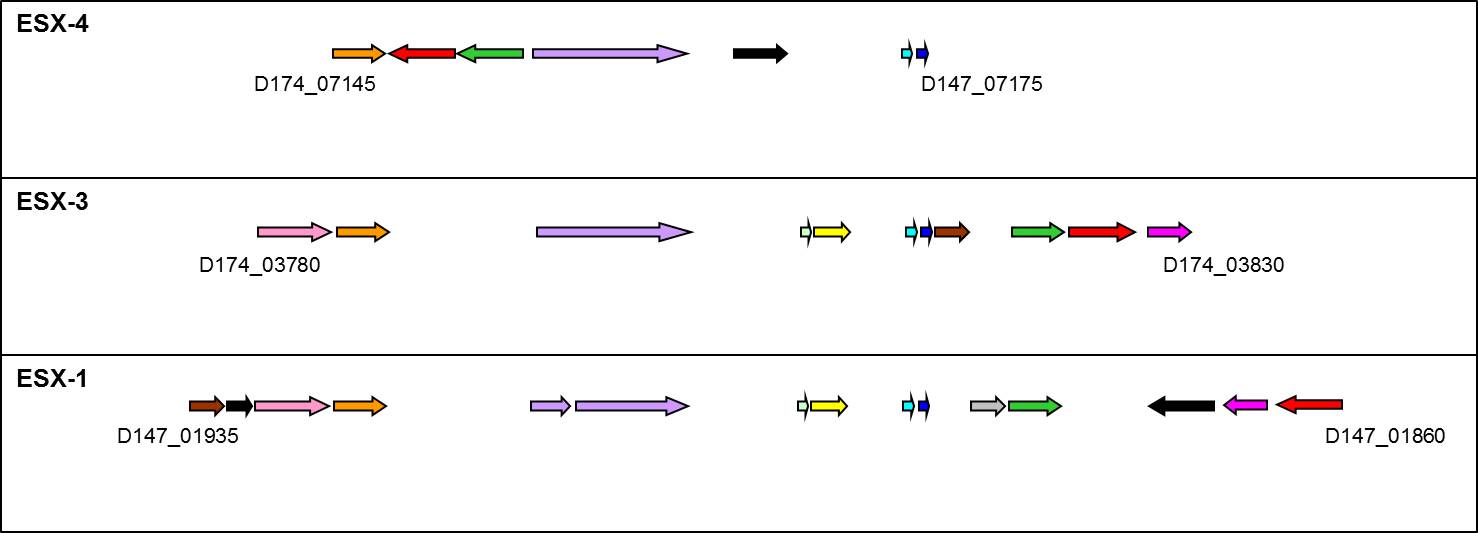
*

*M. neoaurum*

*
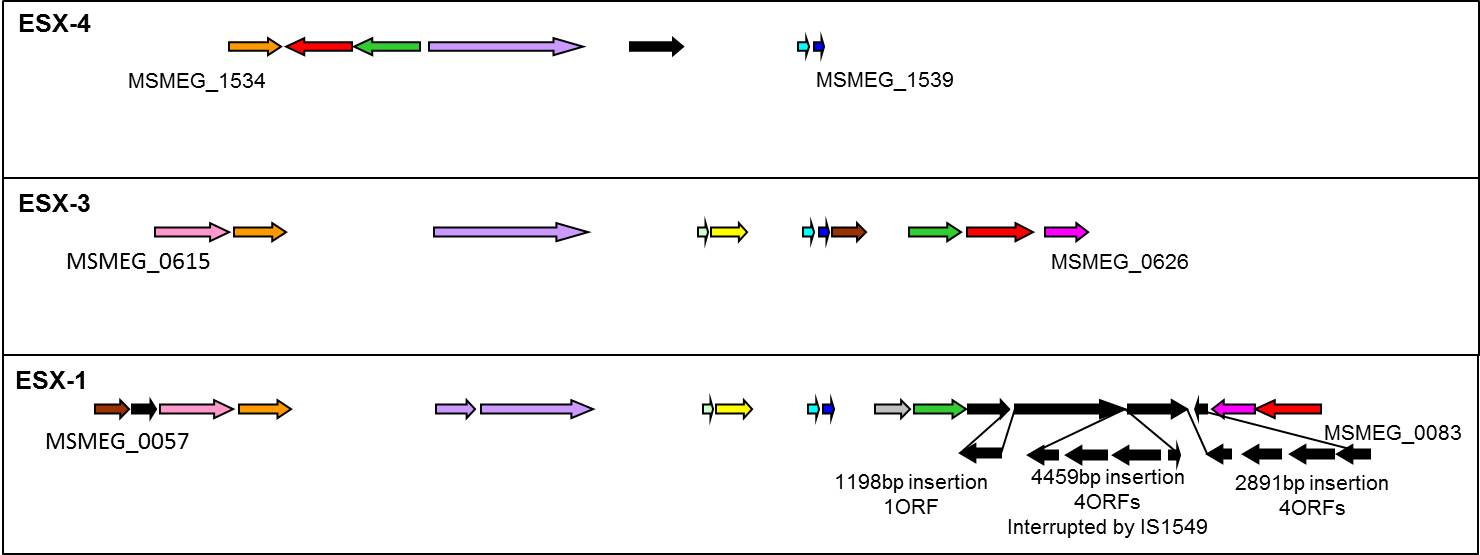
M. smegmatis* mc^2^155

*
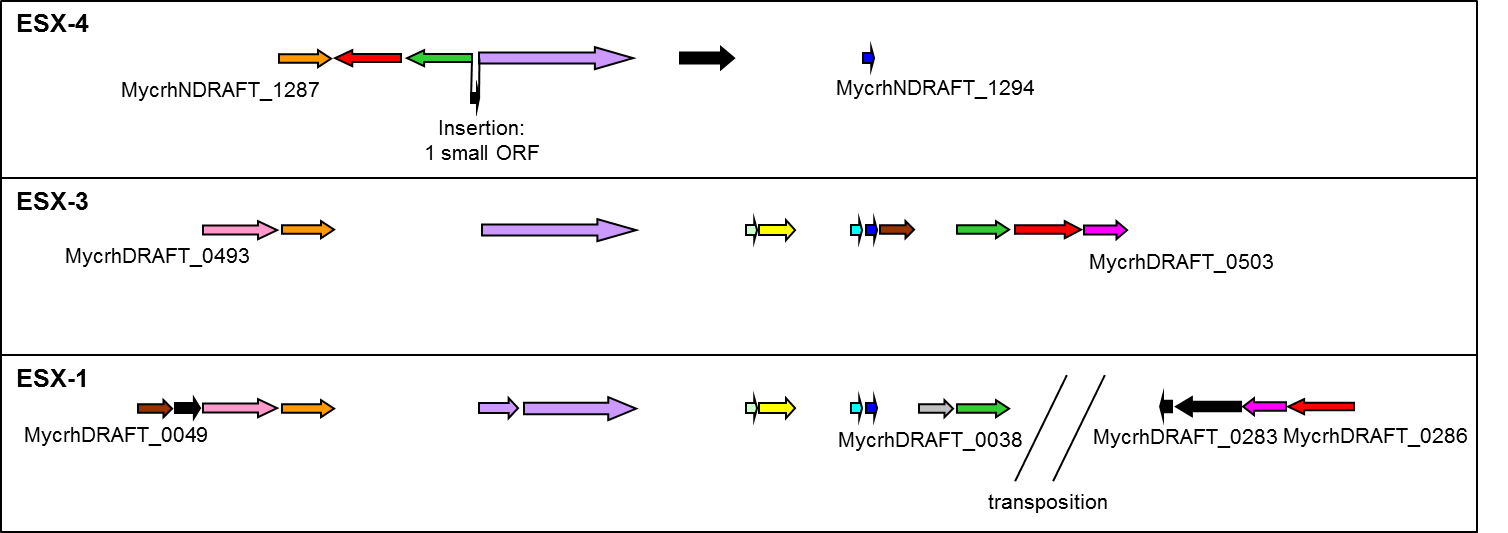
M. rhodesiae*

*
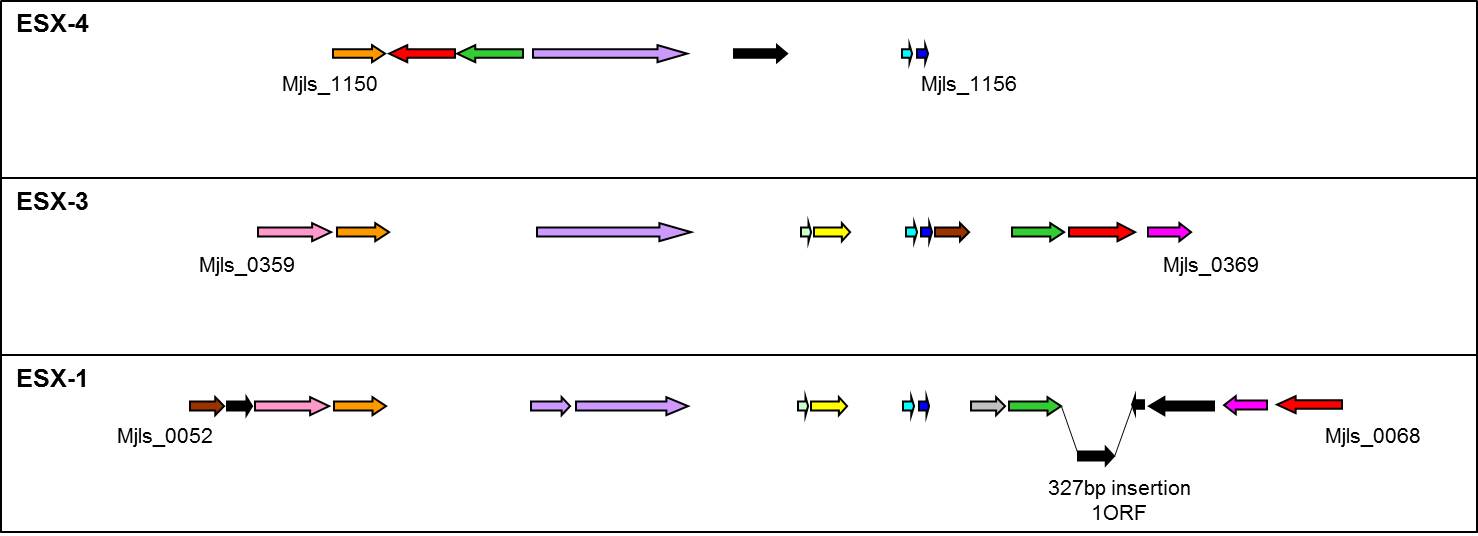
M. sp.* JLS

*
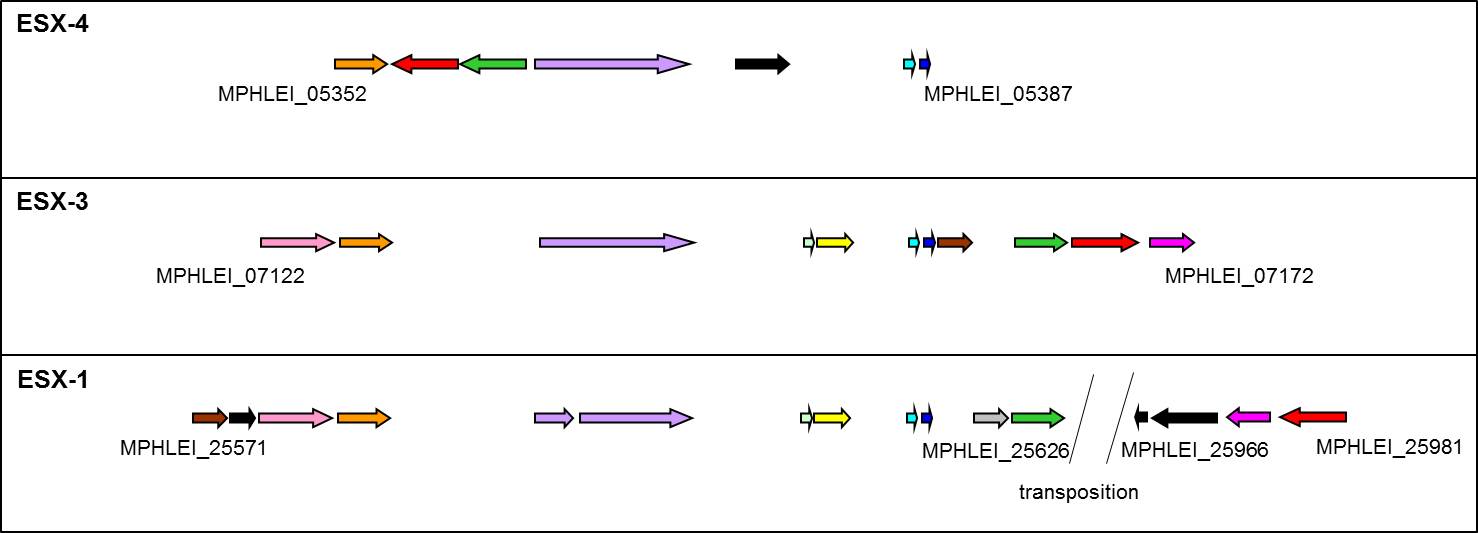
M. phlei*

*
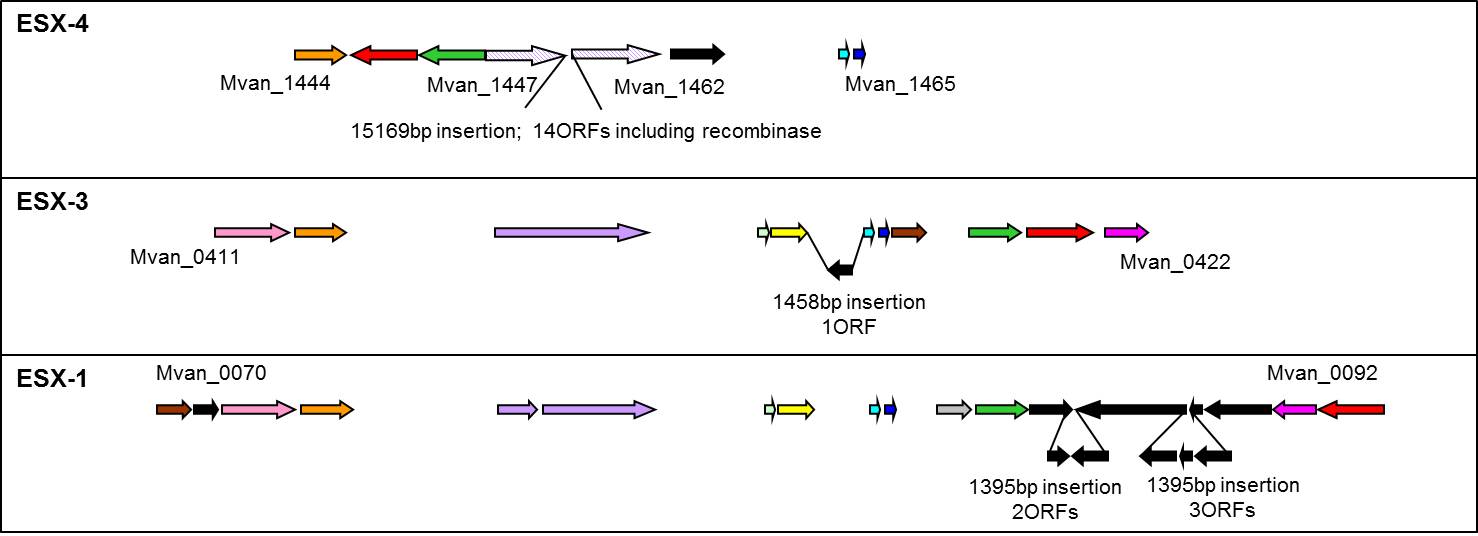
M. vanbaalenii*

*
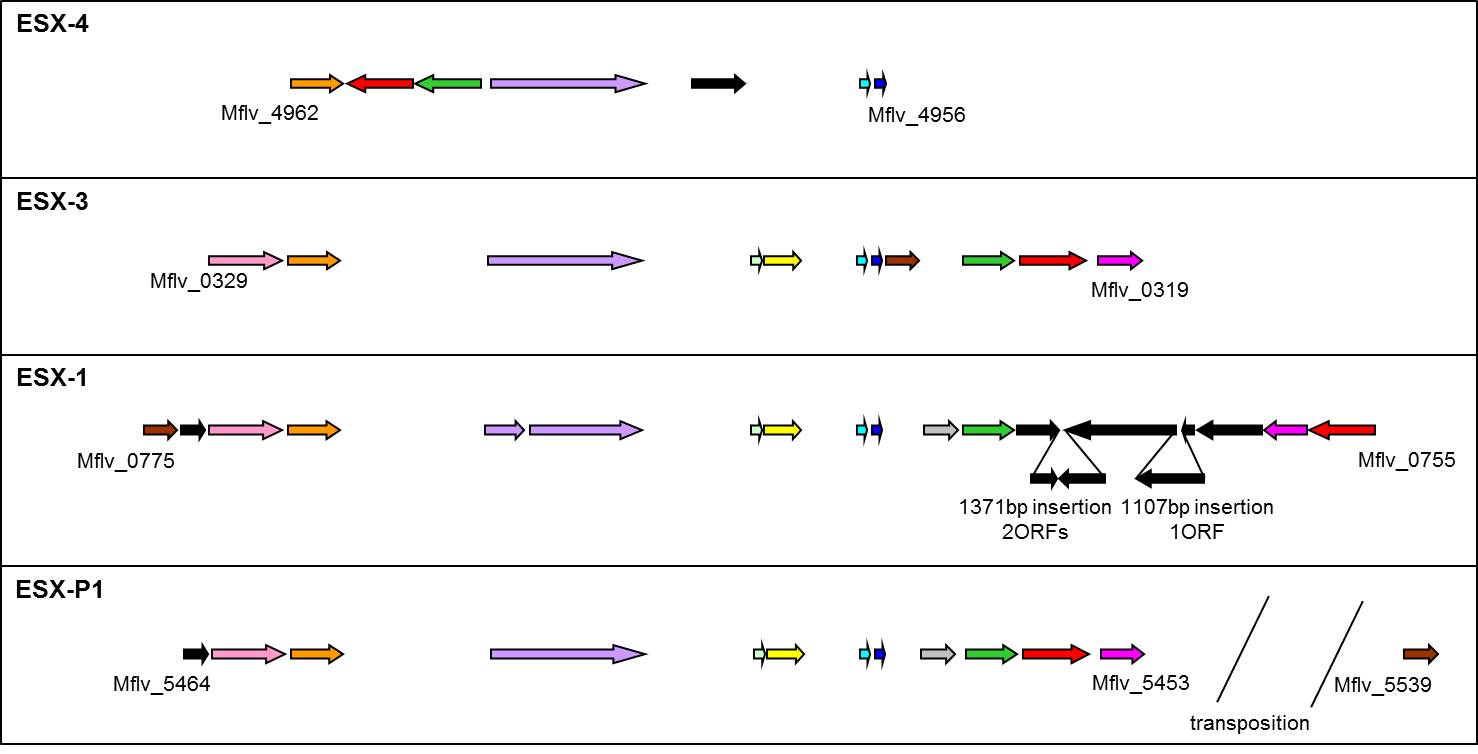
M. gilvum*

*
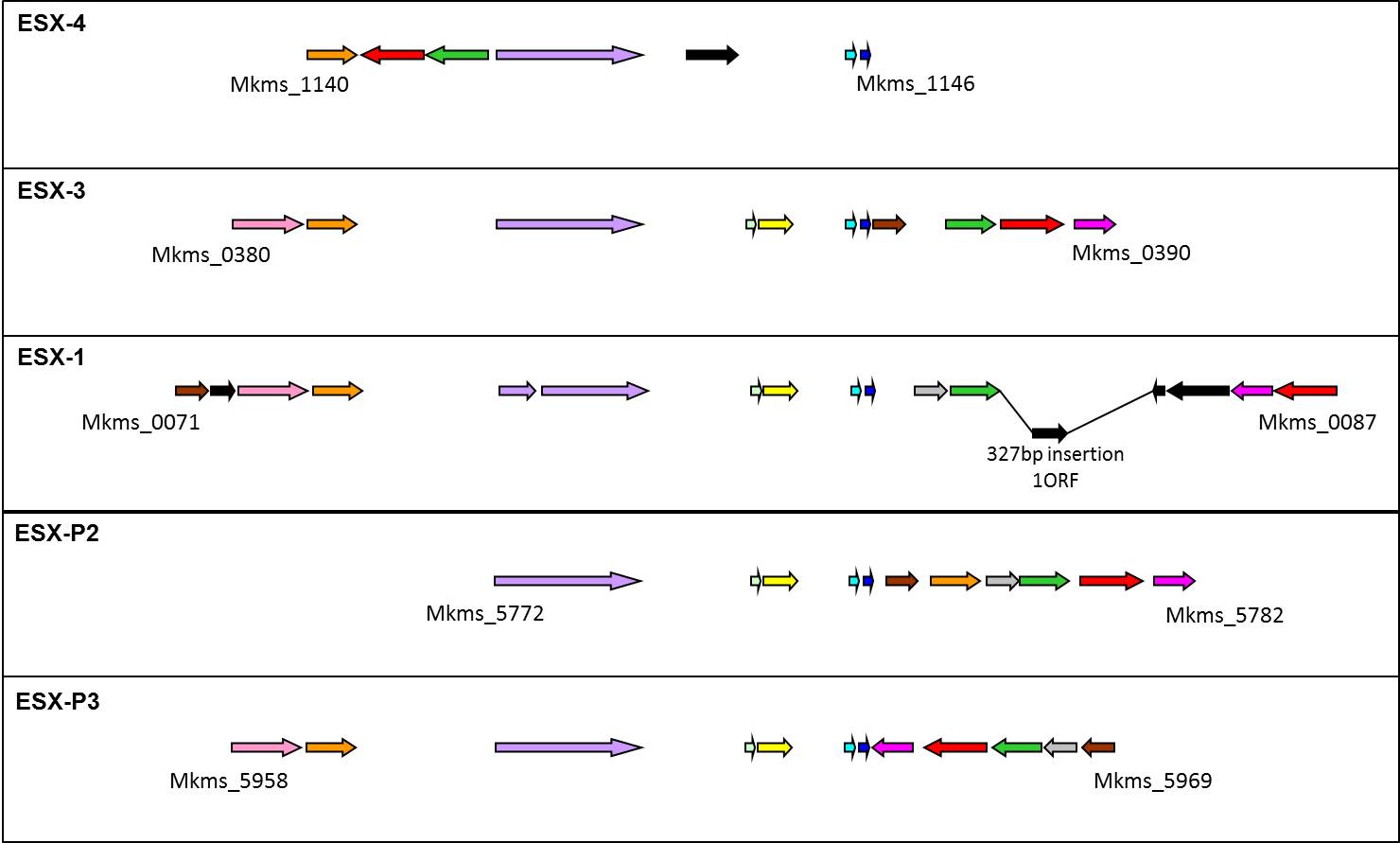
M. sp.* KMS

*
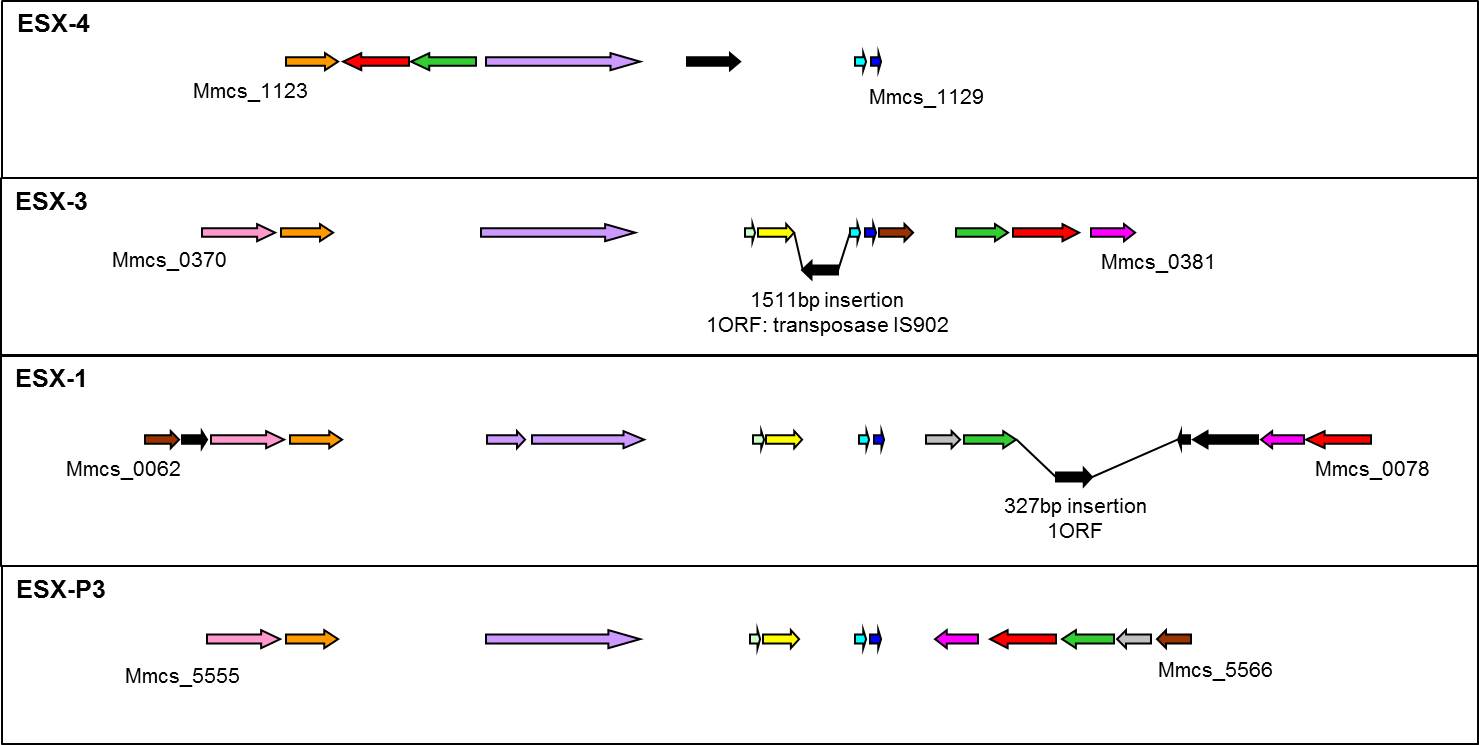
M. sp.* MCS*
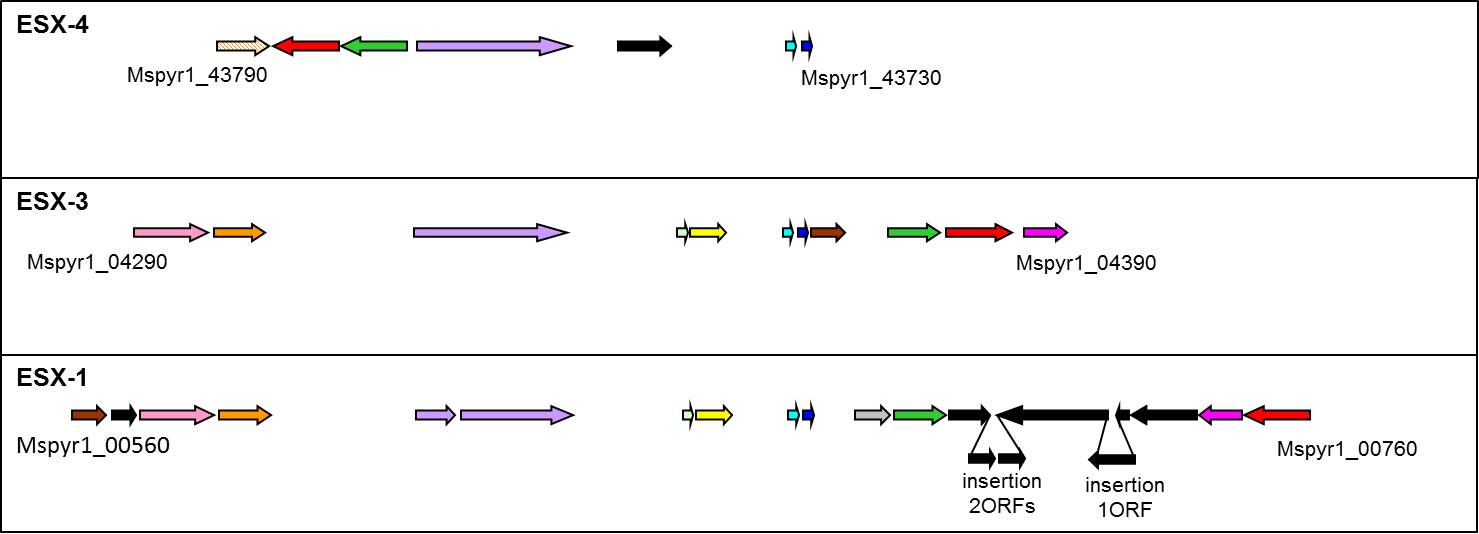
M. sp.* Spyr1

*
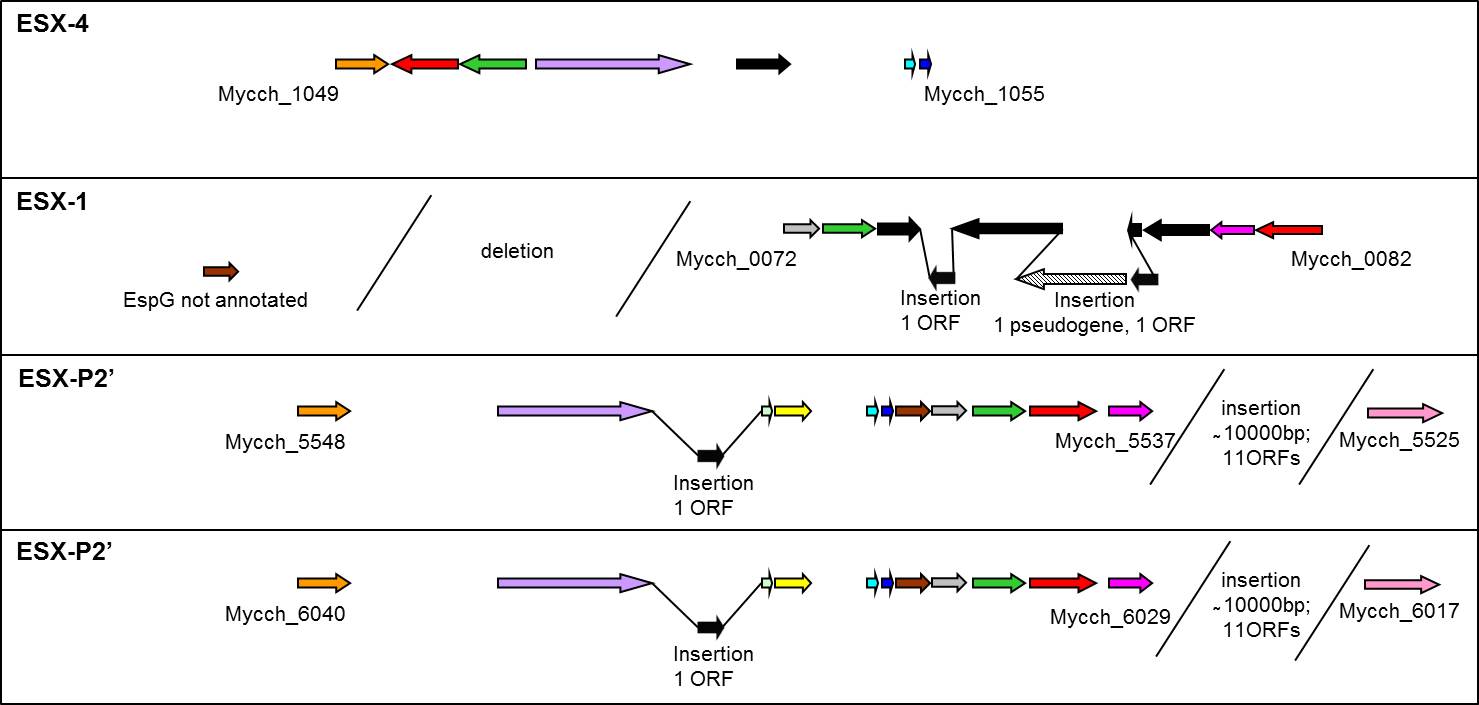
M. chubuense*

*
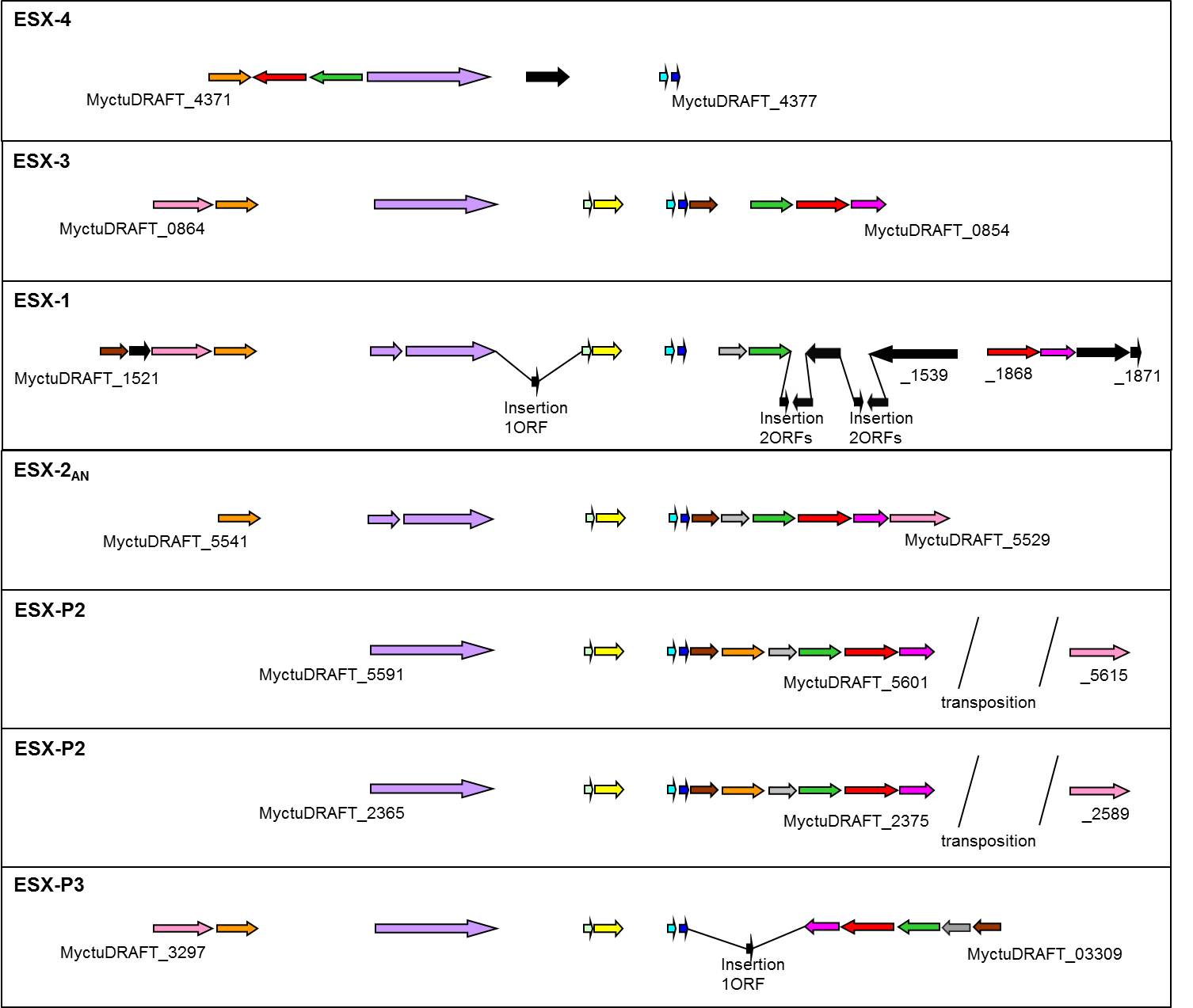
M. tusciae*

*
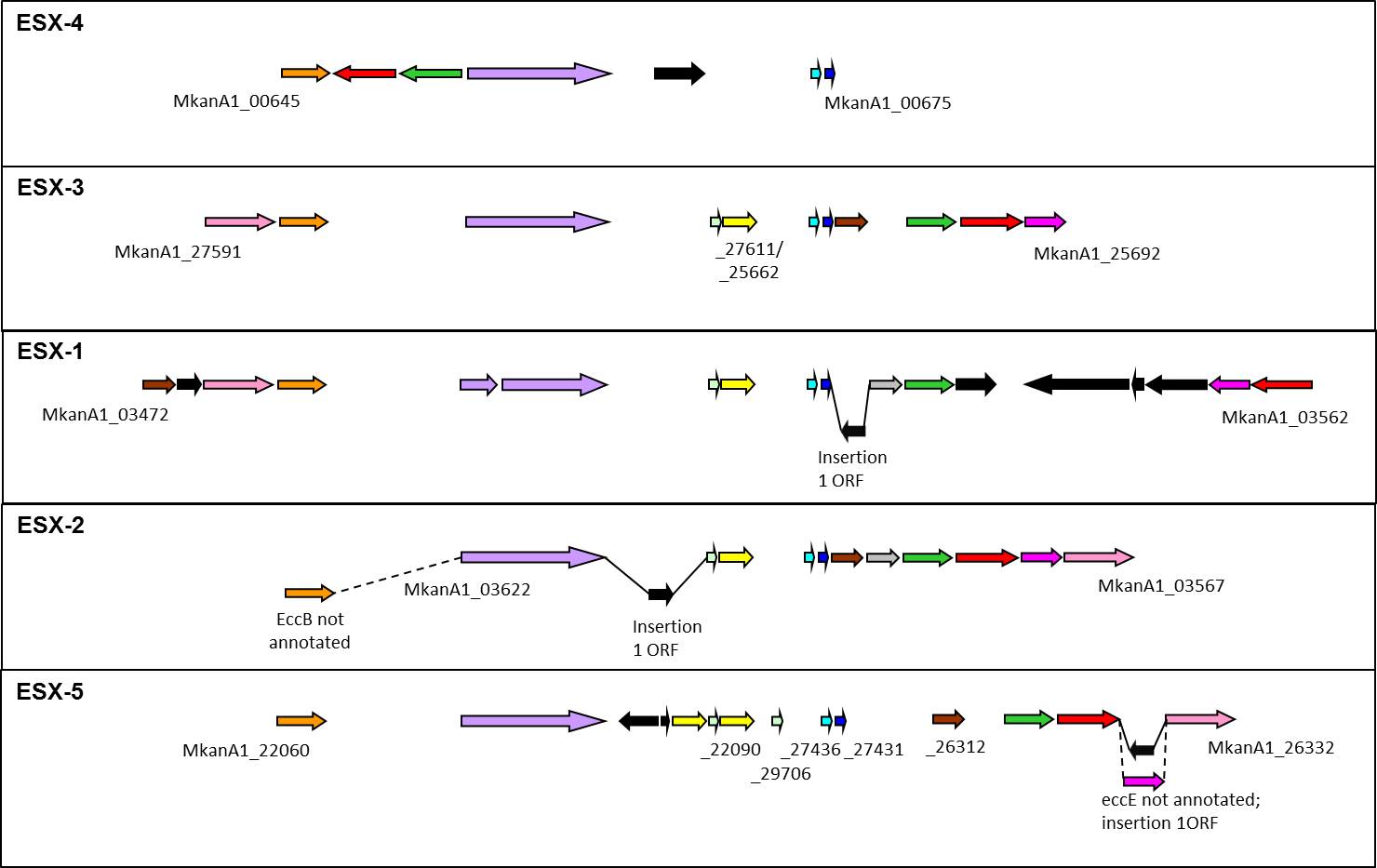
M. kansasii*

*
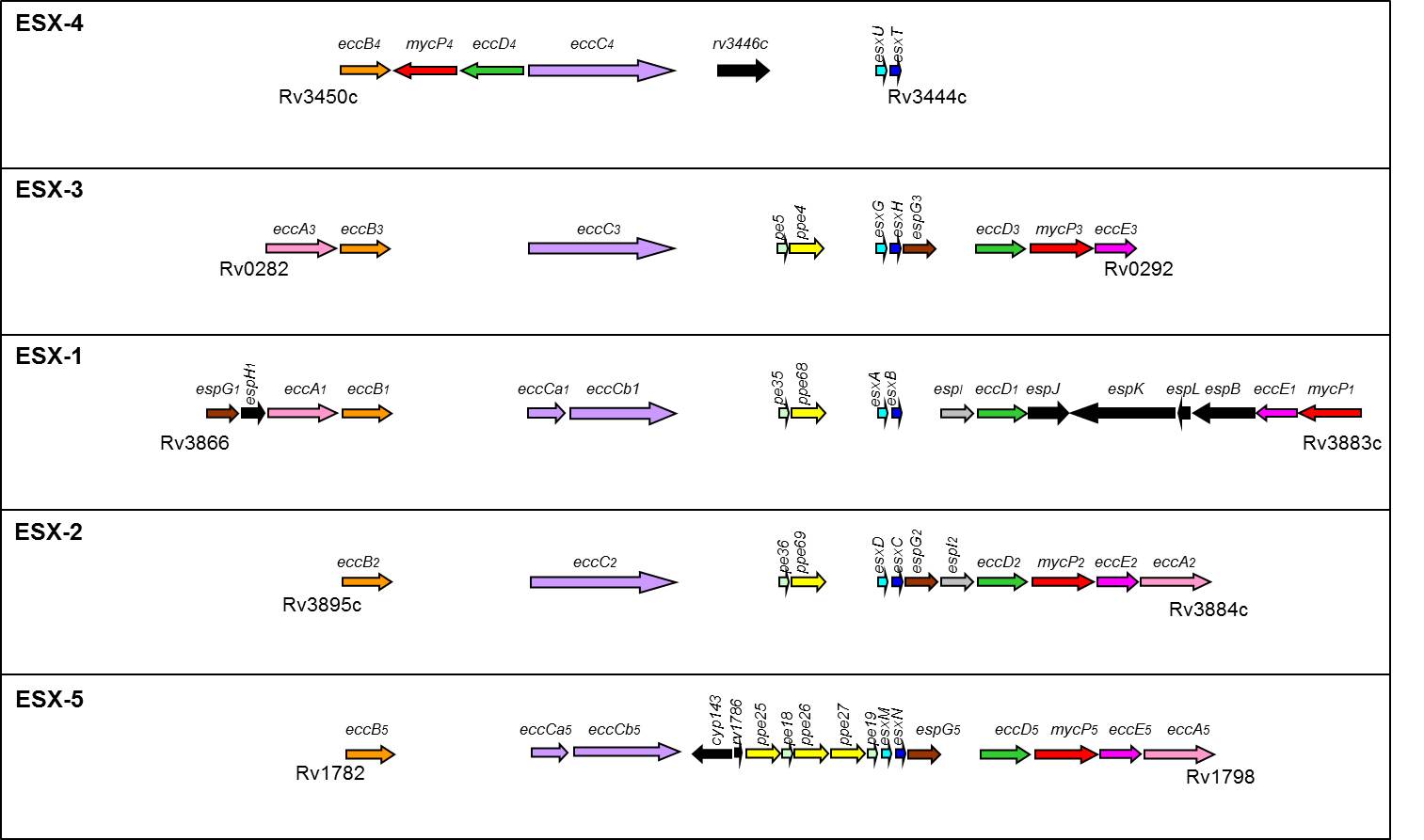
M. tuberculosis*

*
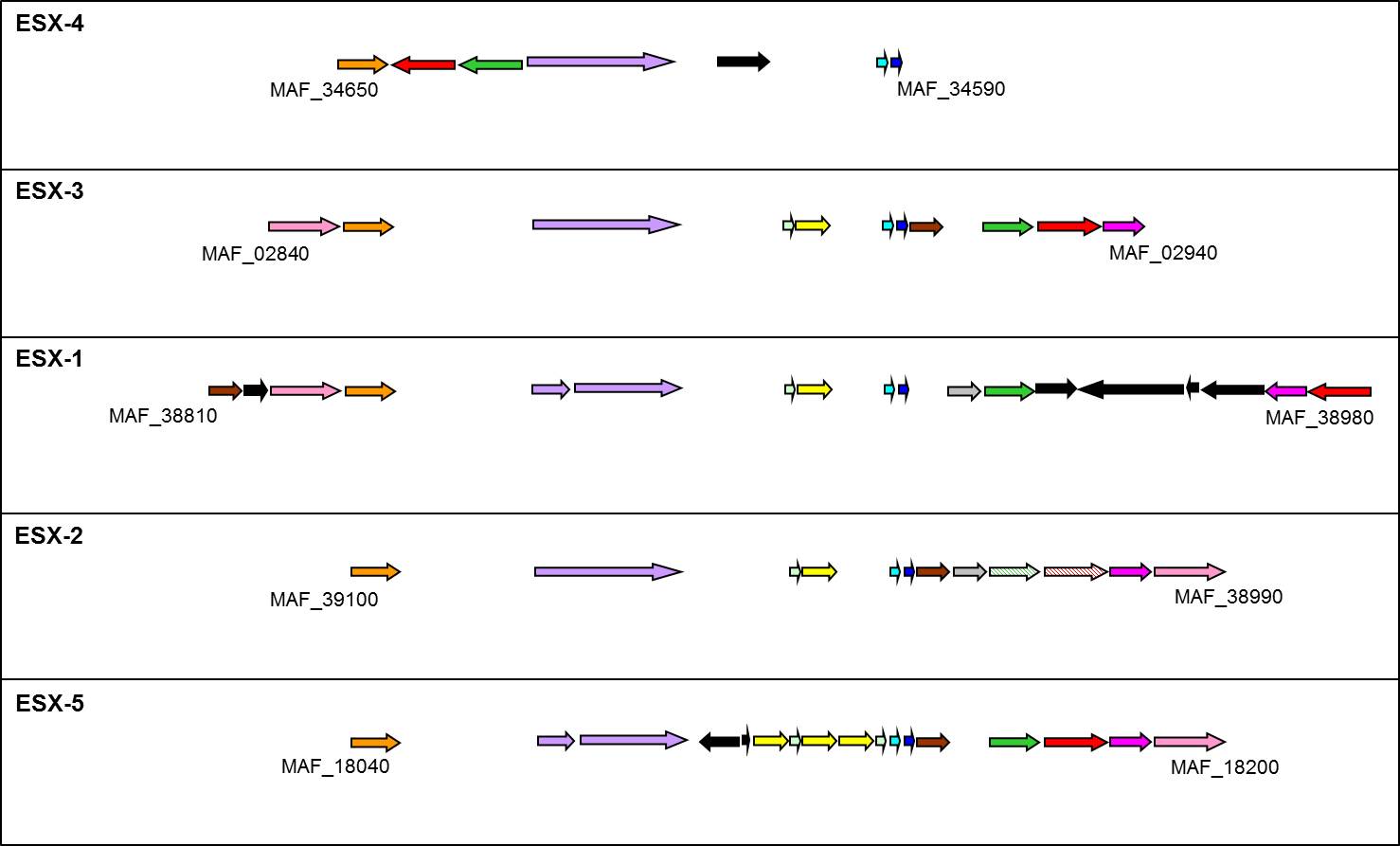
M. africanum*

*
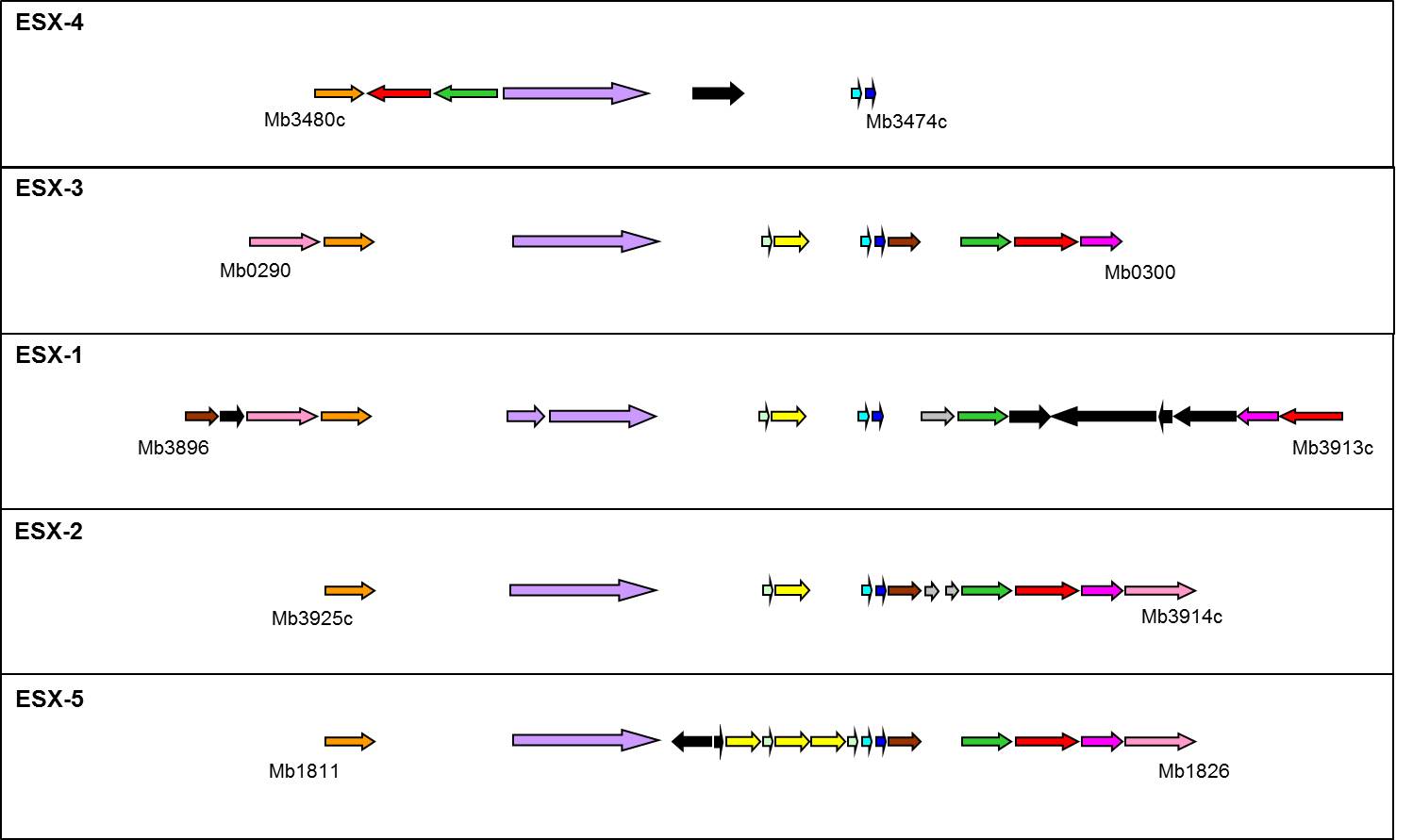
M. bovis*

*
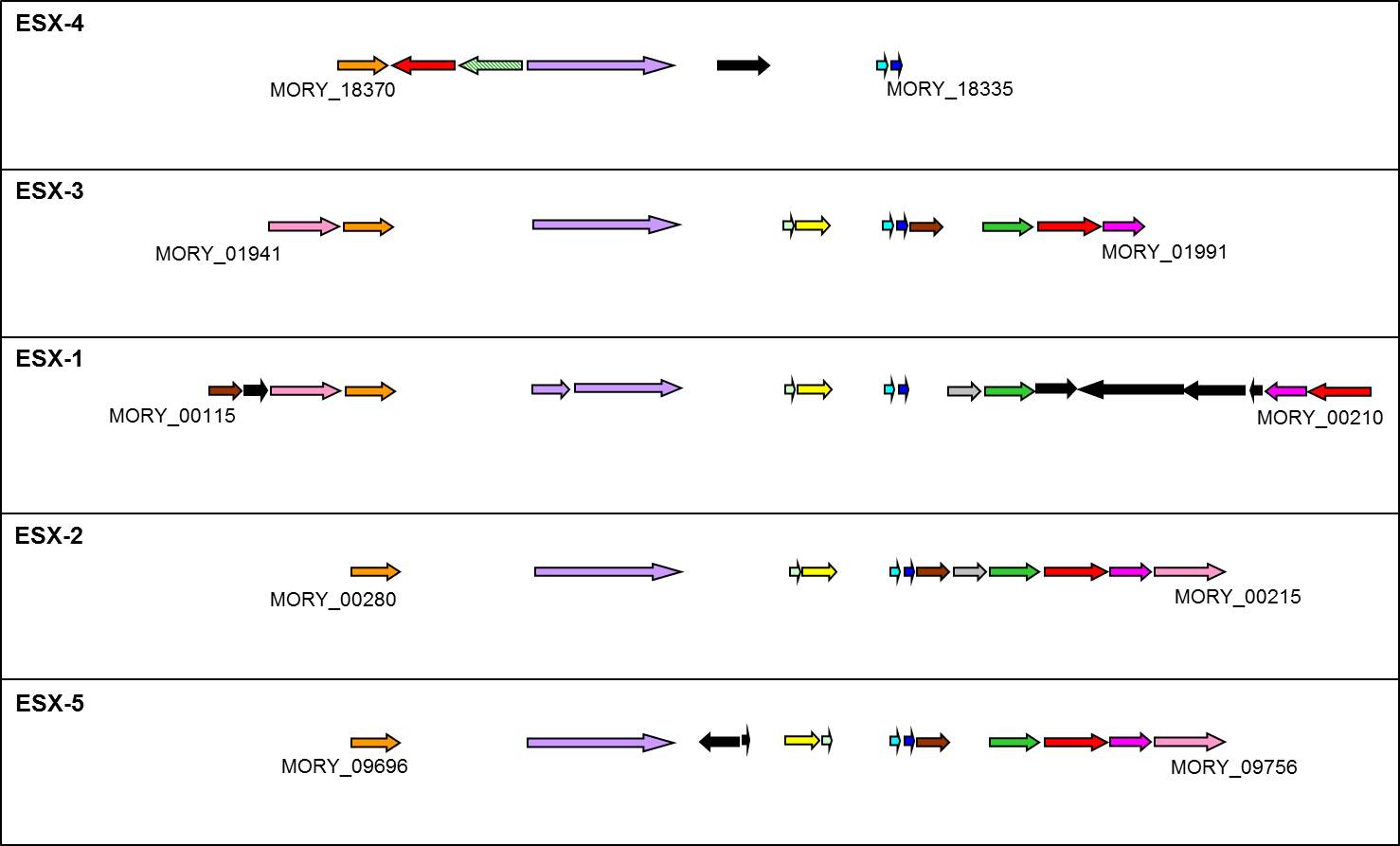
M. orygis*

*
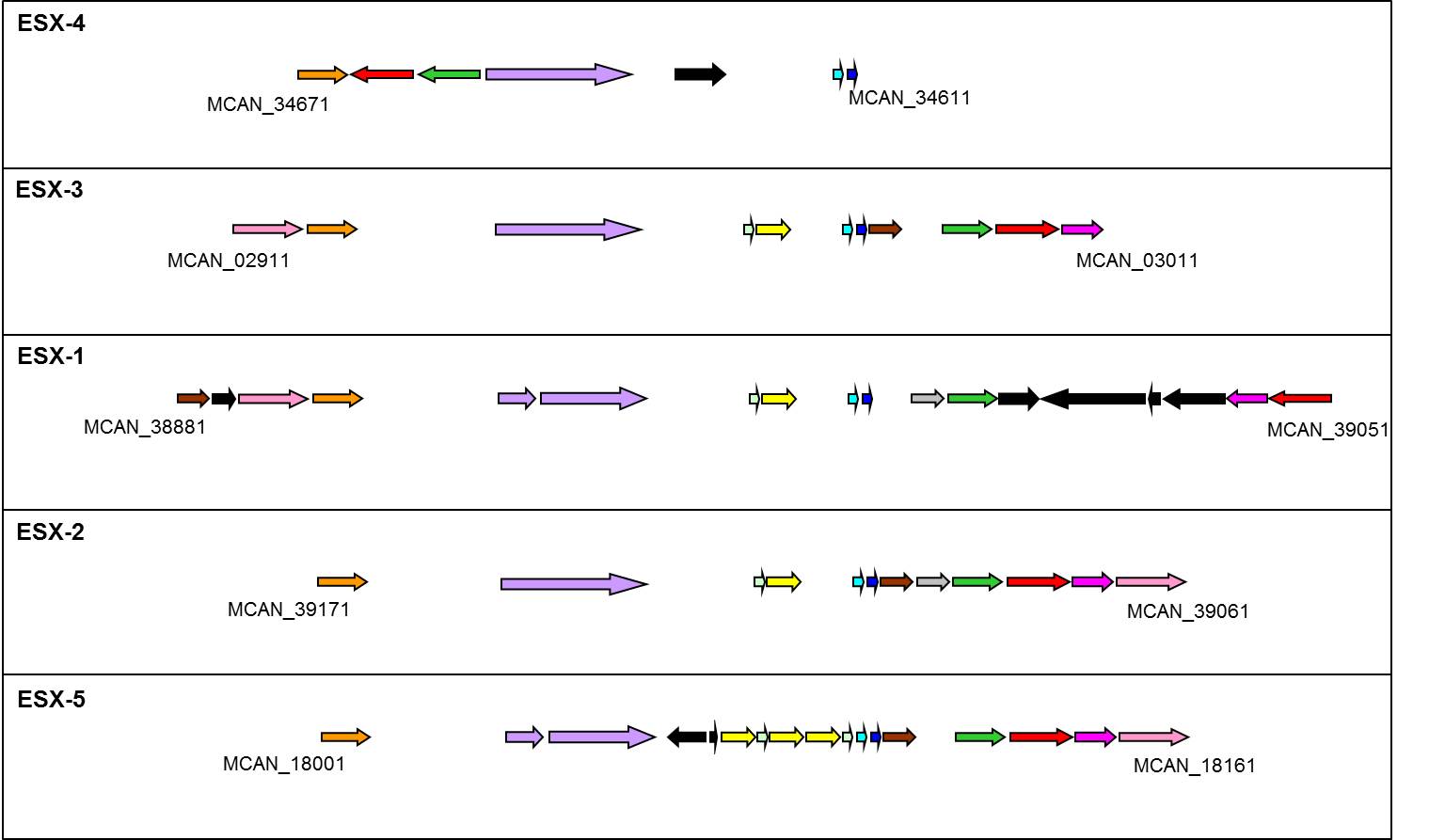
M. canetti*

*
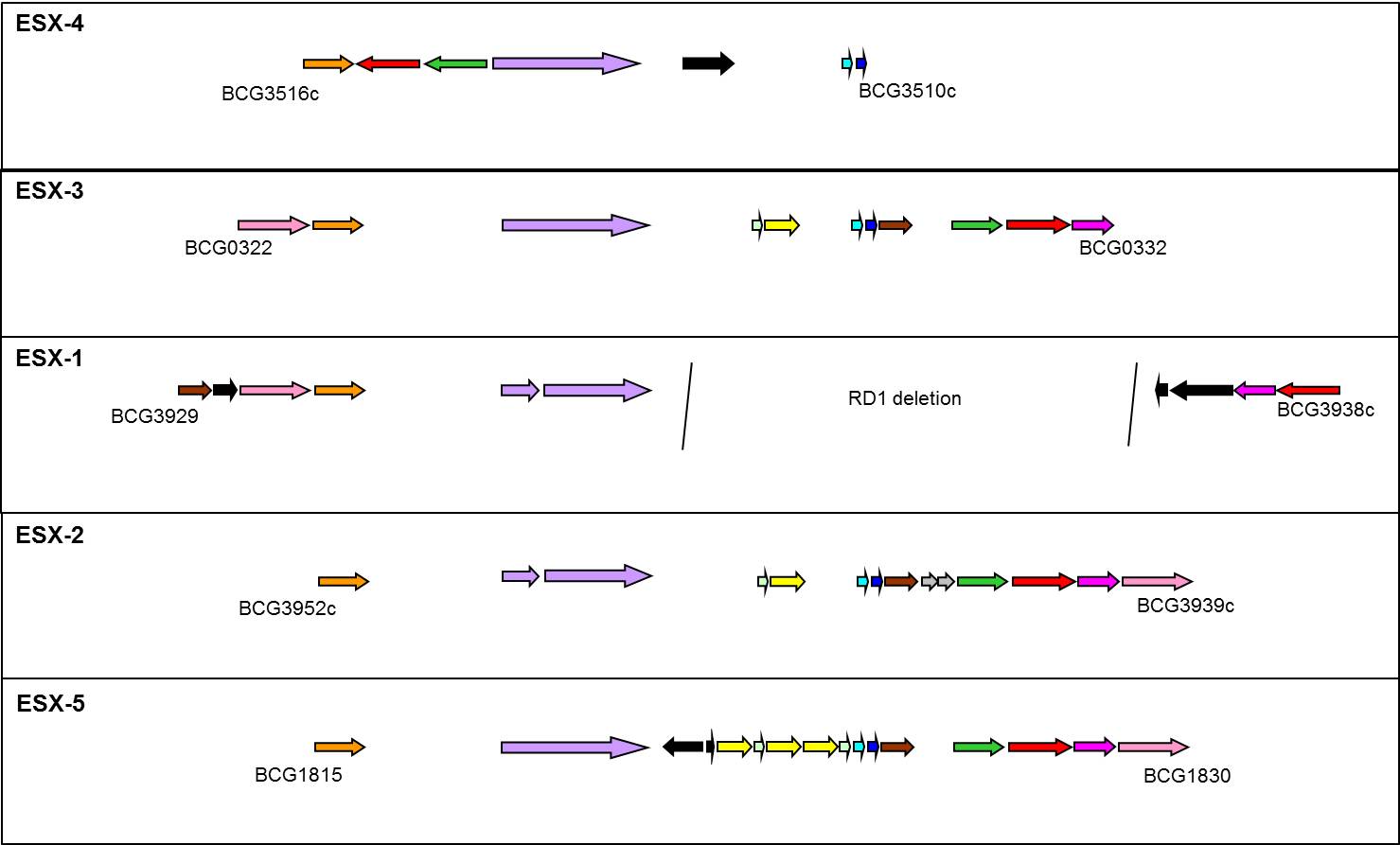
M. bovis BCG*

*
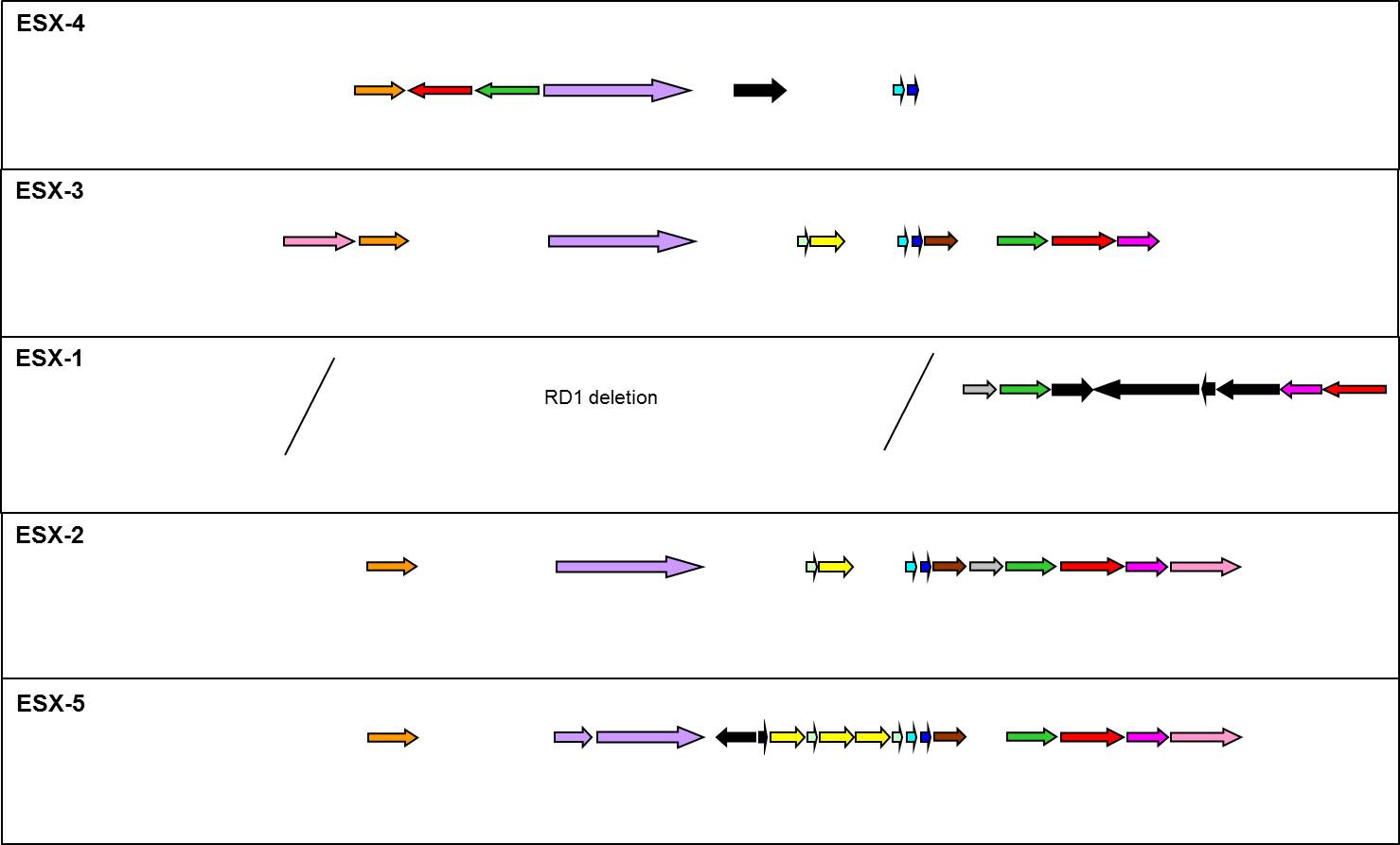
*

*M. microti*

*
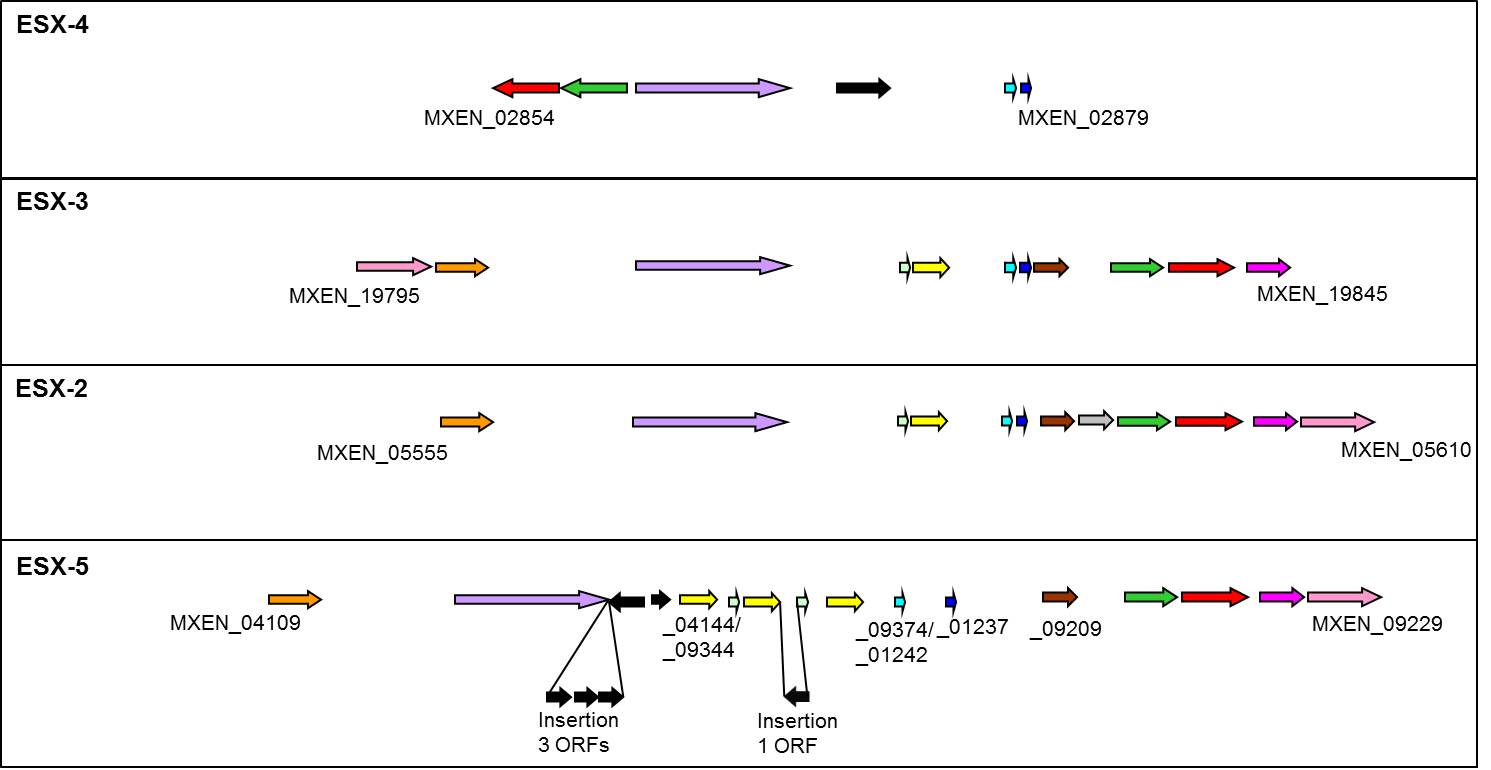
M. xenopi*

*
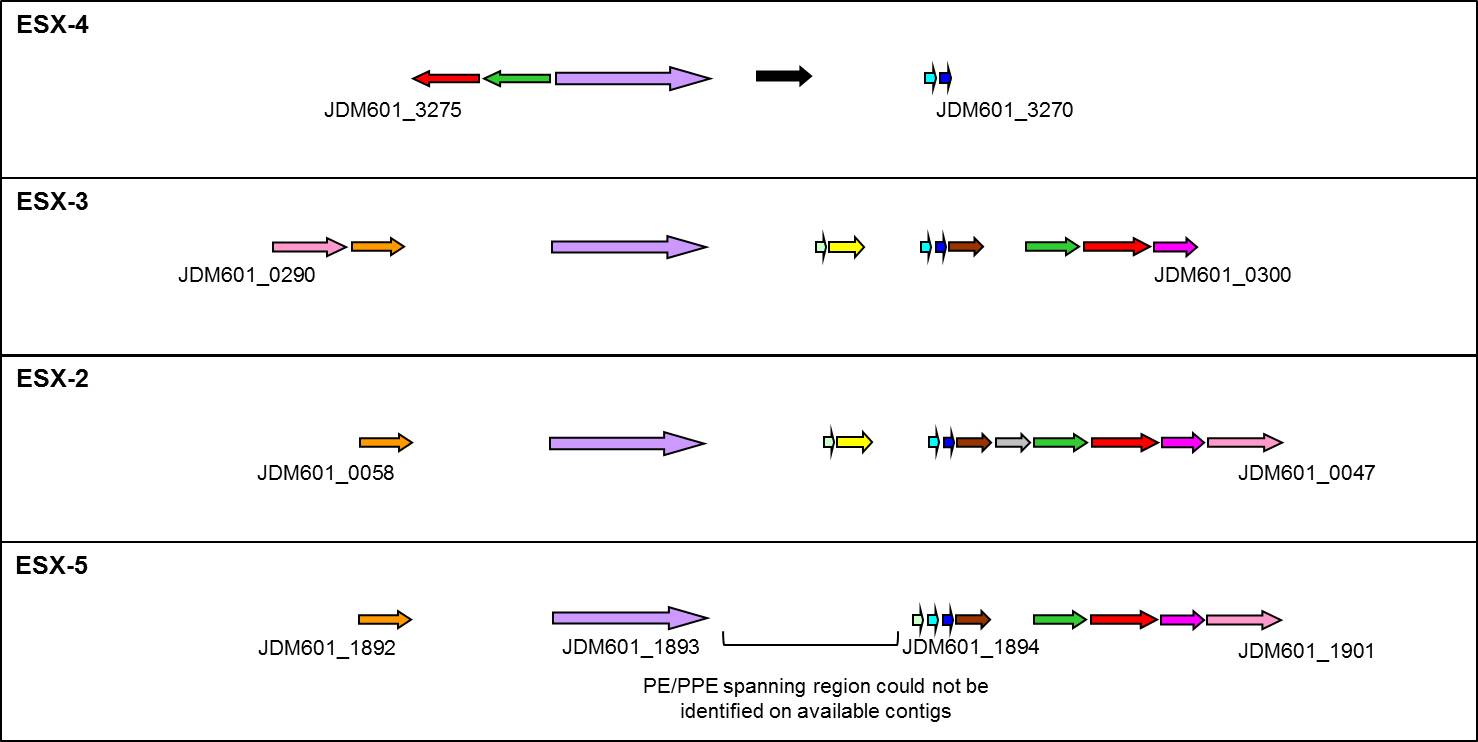
M. sp.* JDM601


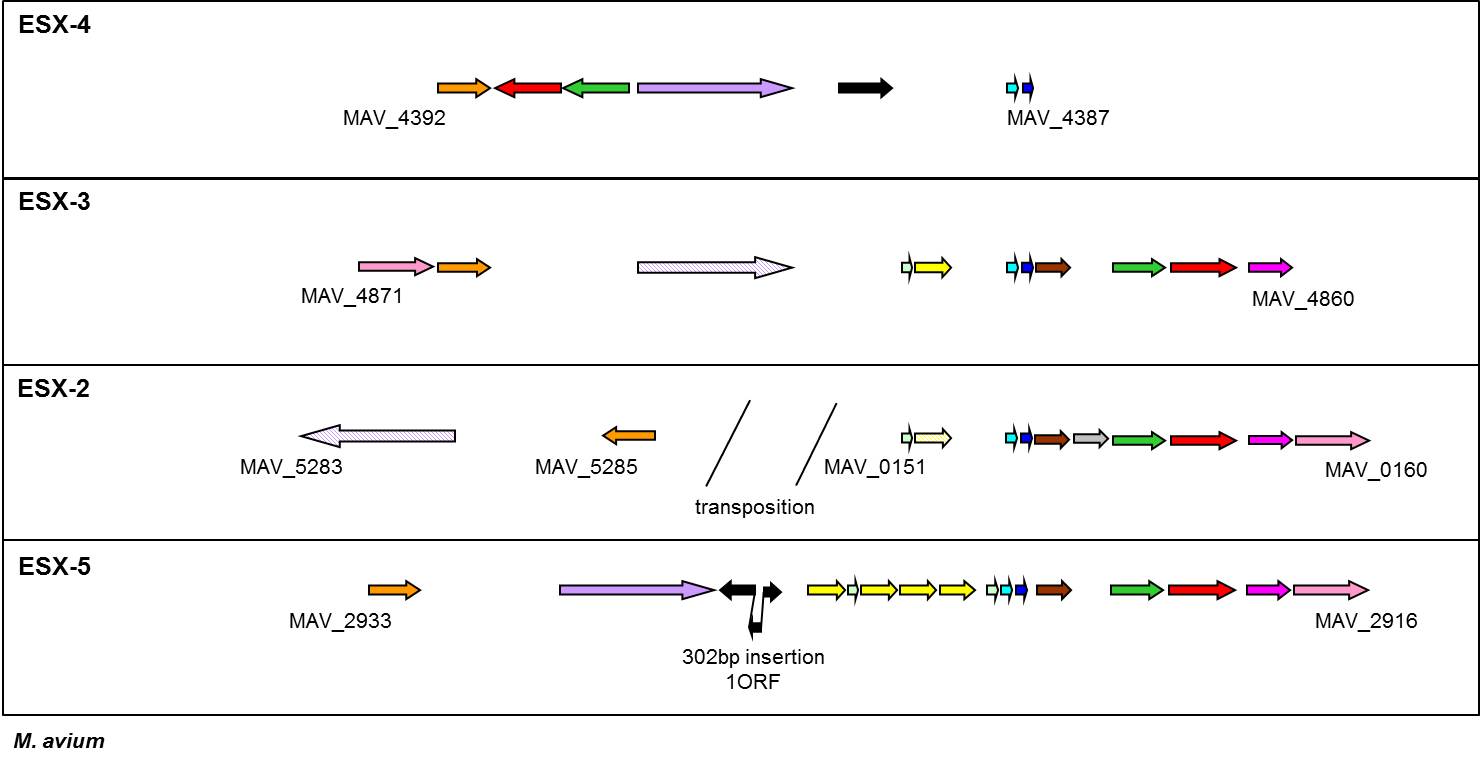
*M. avium*

*
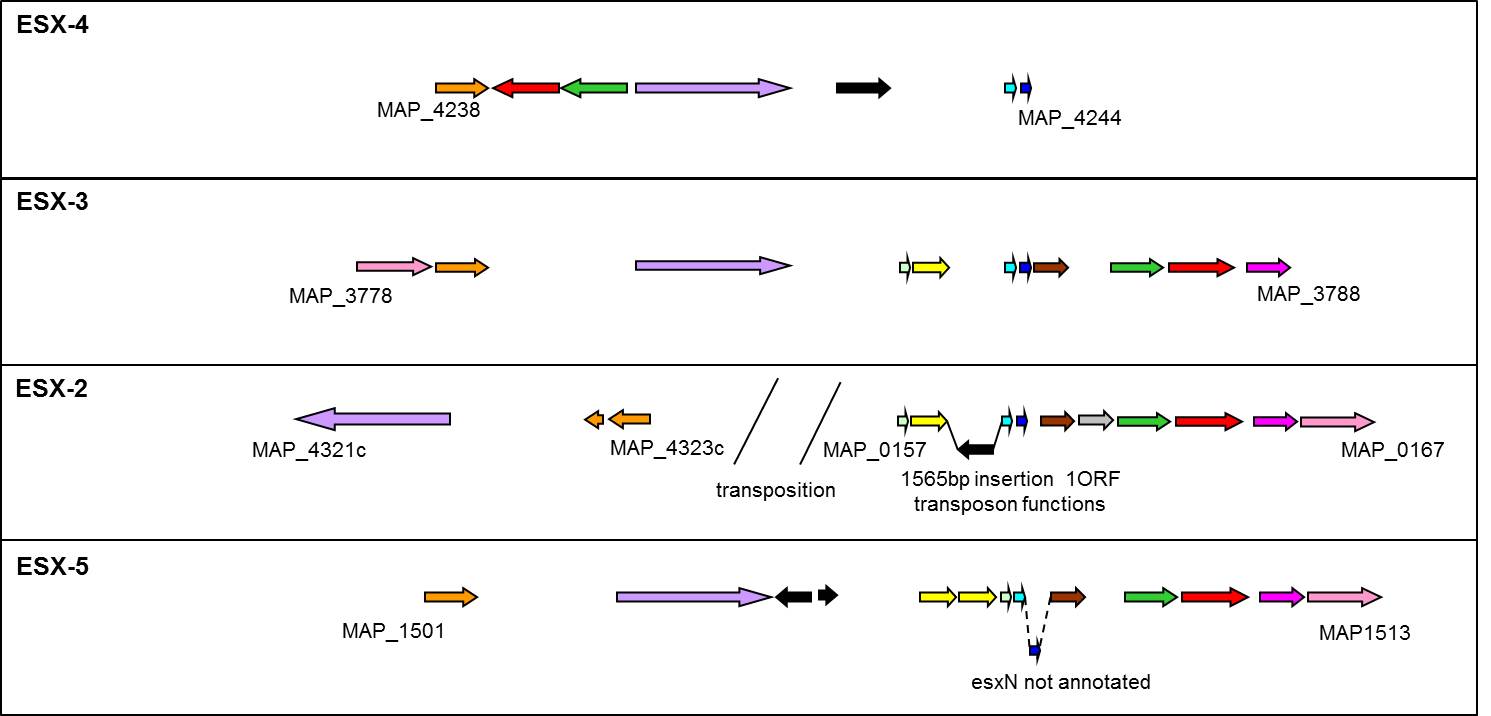
M. avium paratuberculosis*

*
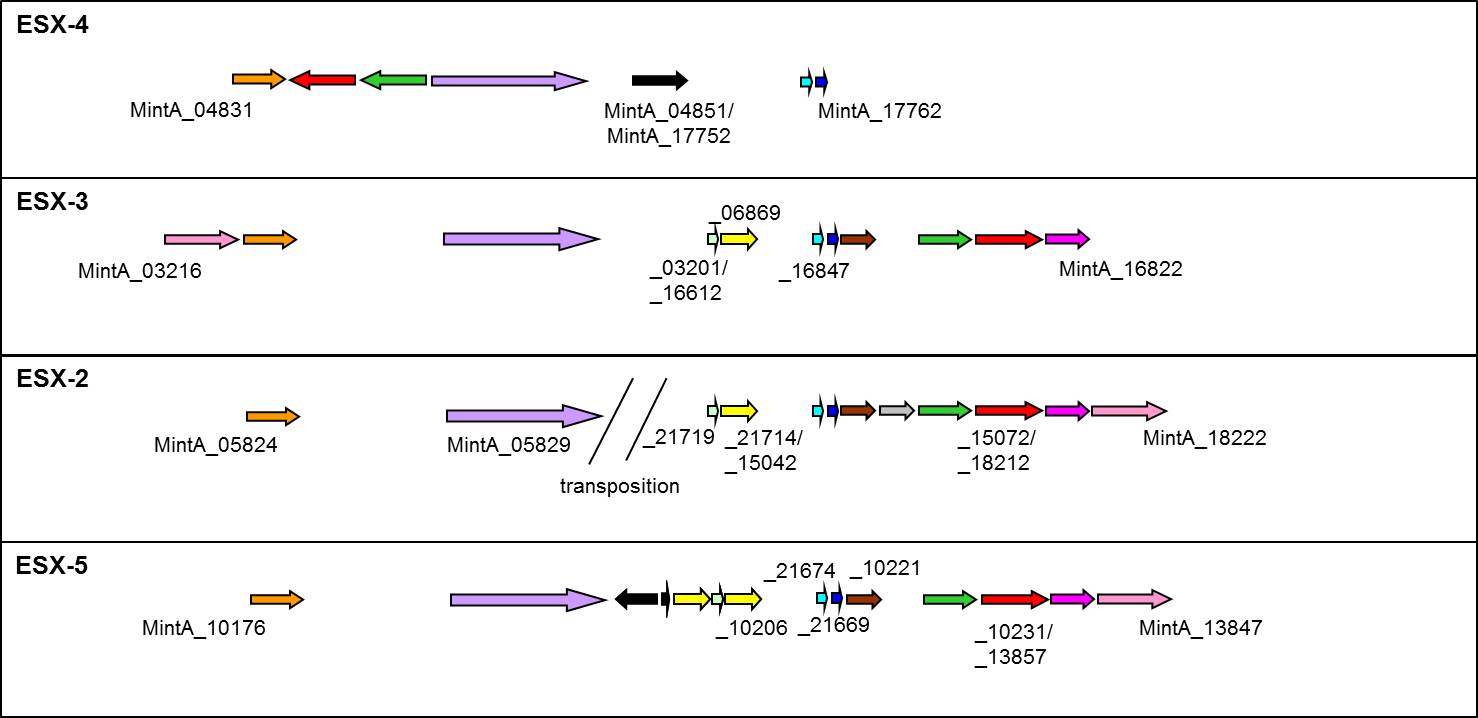
M. intracellulare*

*
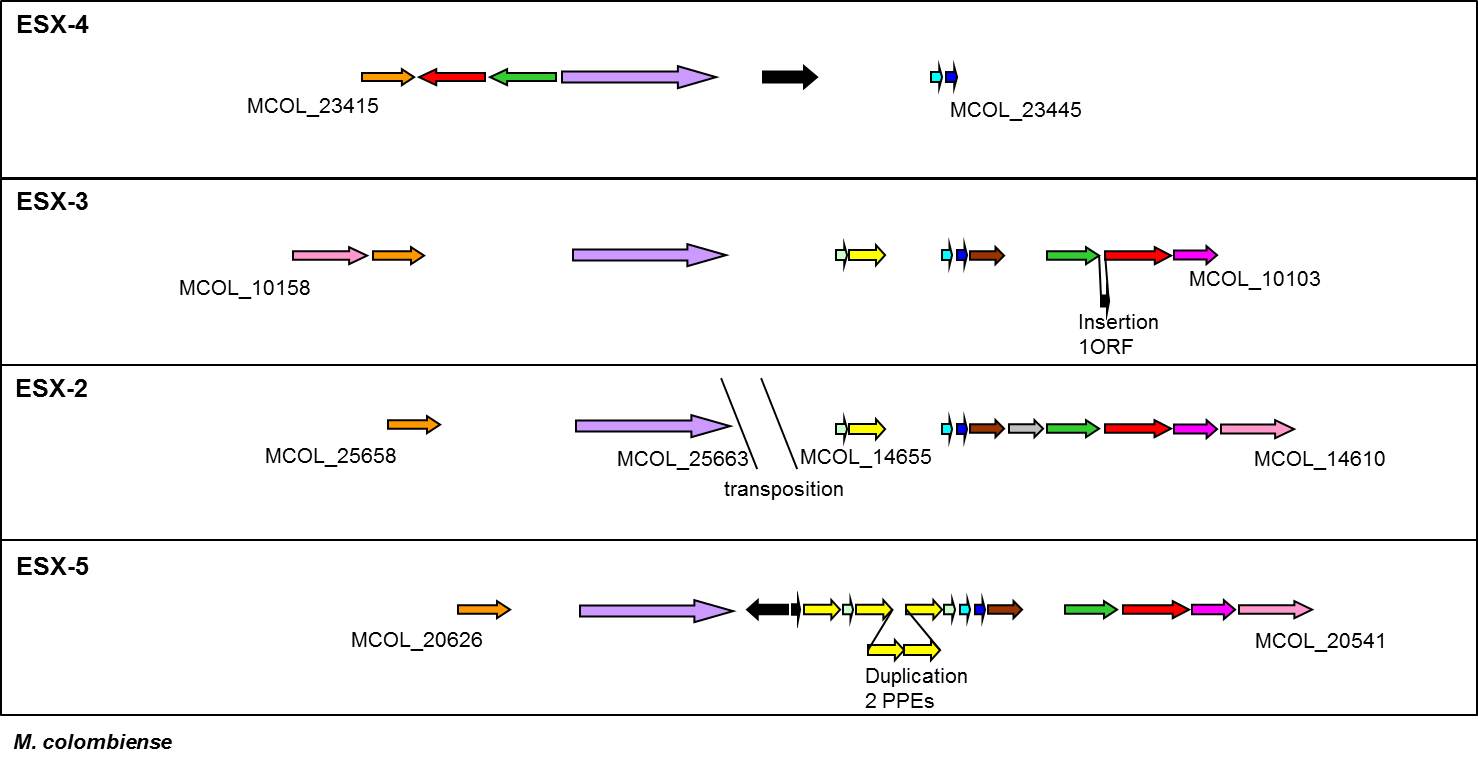
M. colombiense*

*
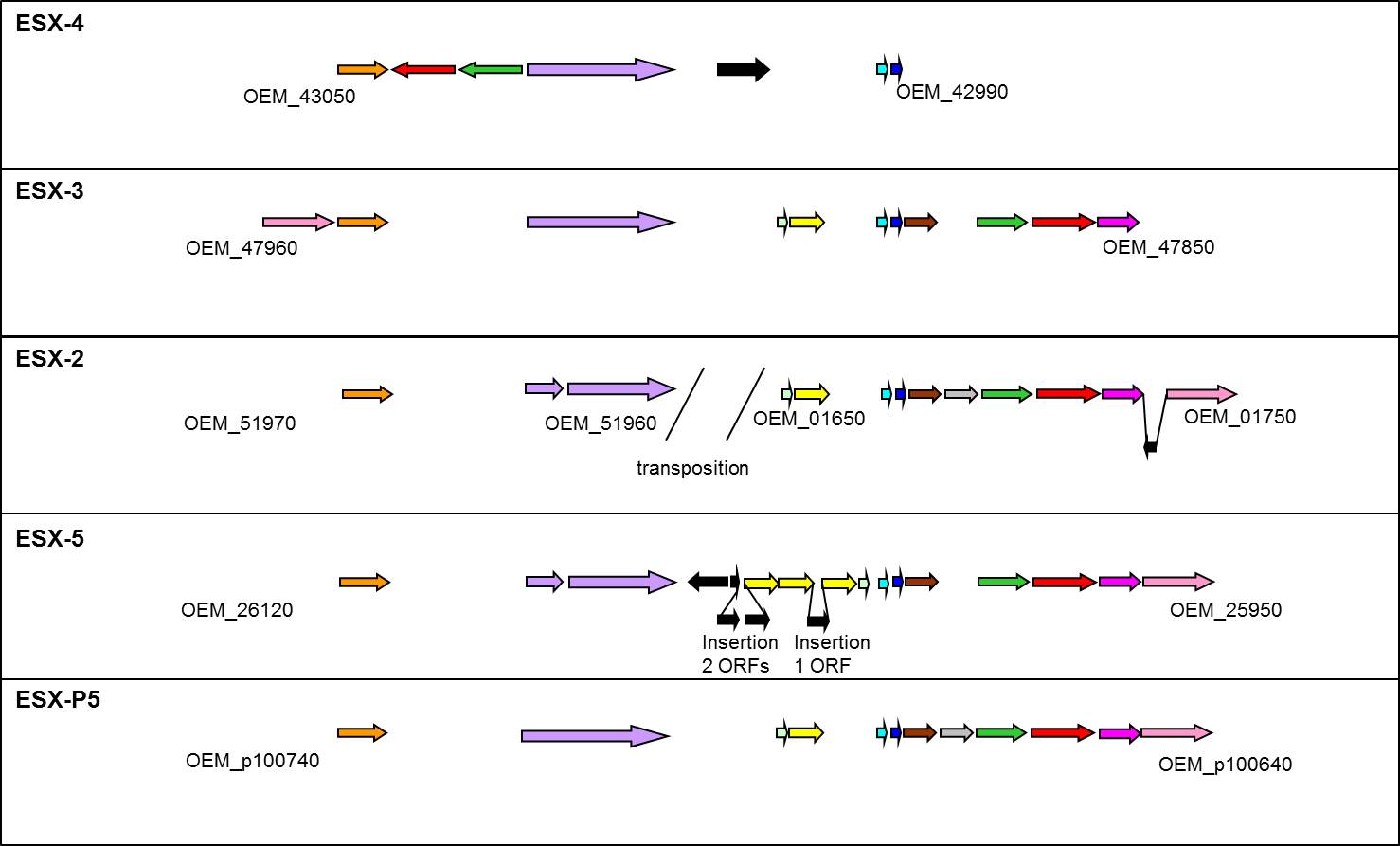
M. yongonense*

*
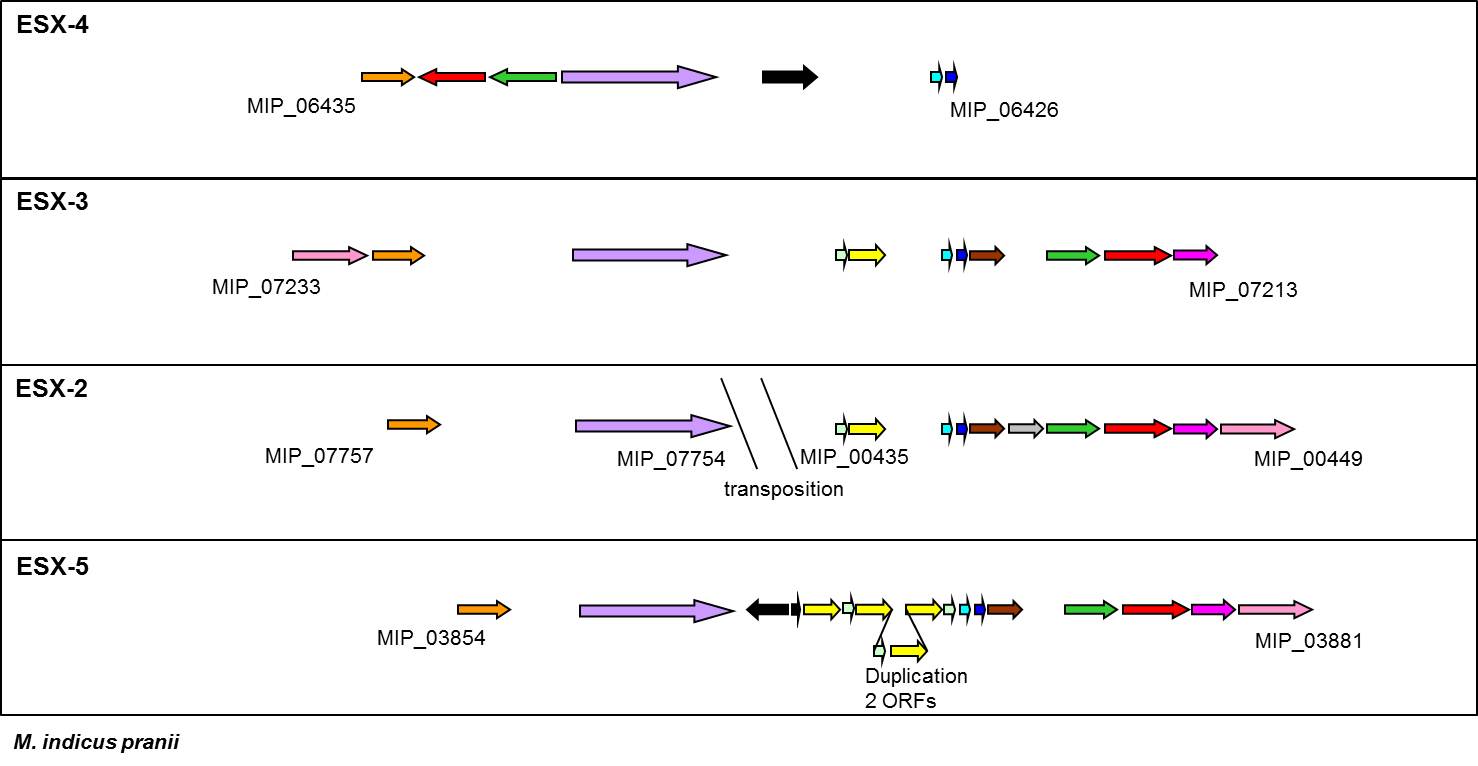
M. indicus pranii*

*
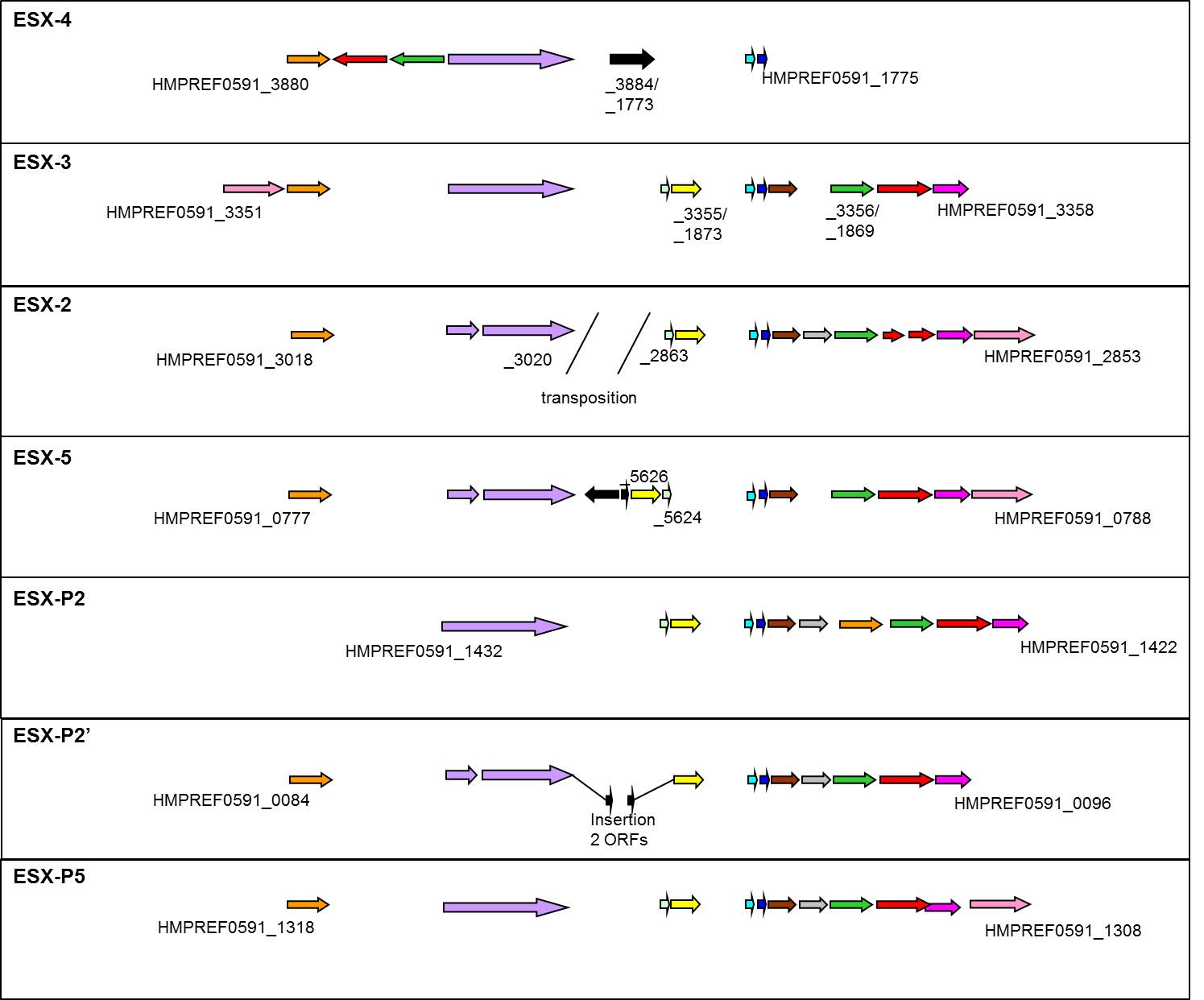
M. parascrofulaceum*

*
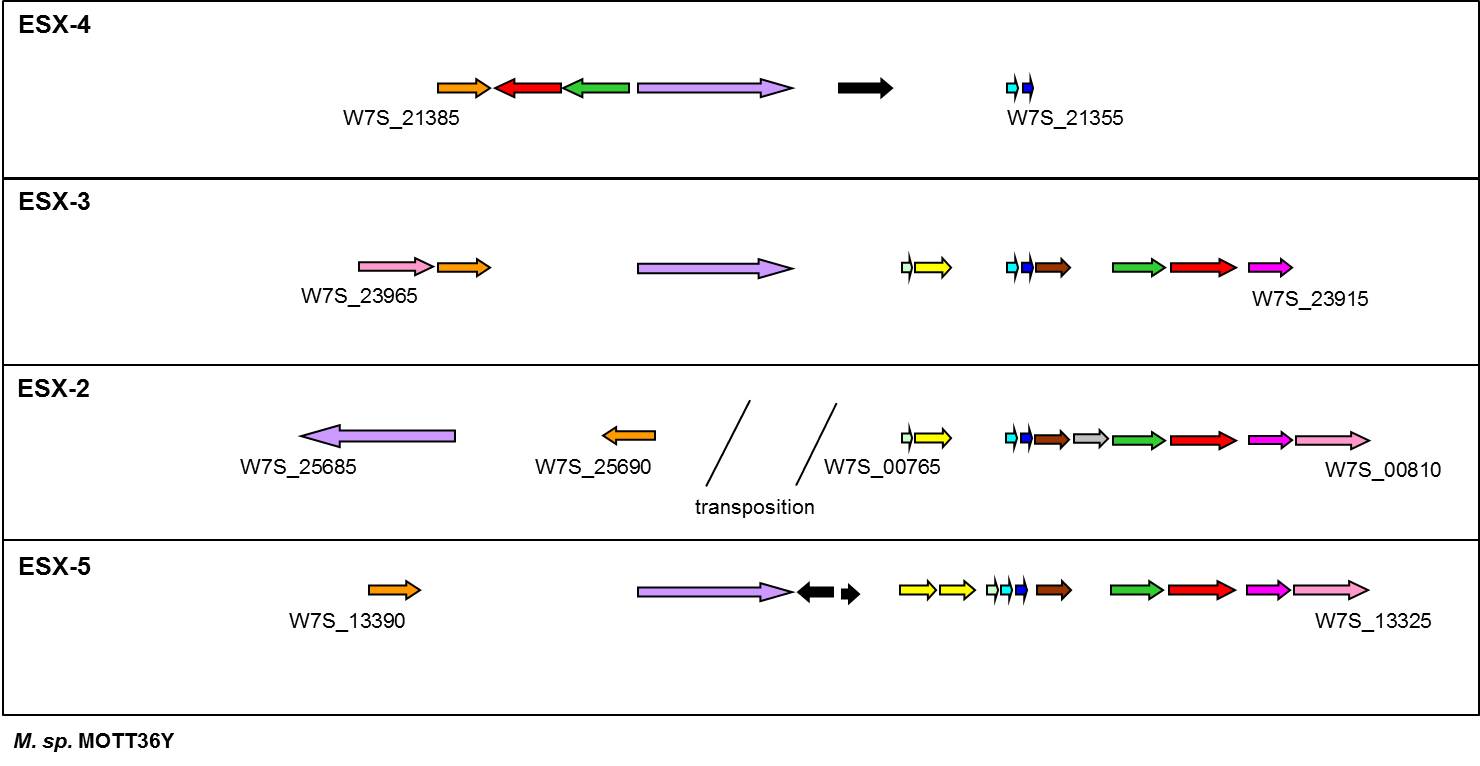
M. sp.* MOTT36Y


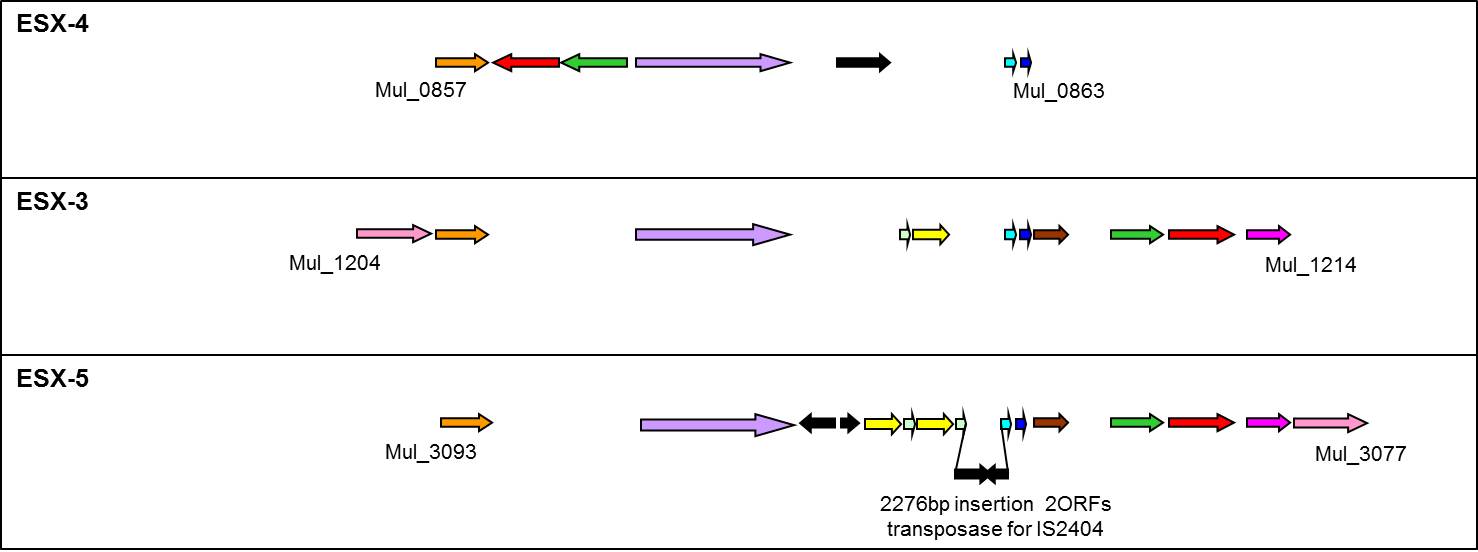
*M. ulcerans*

*
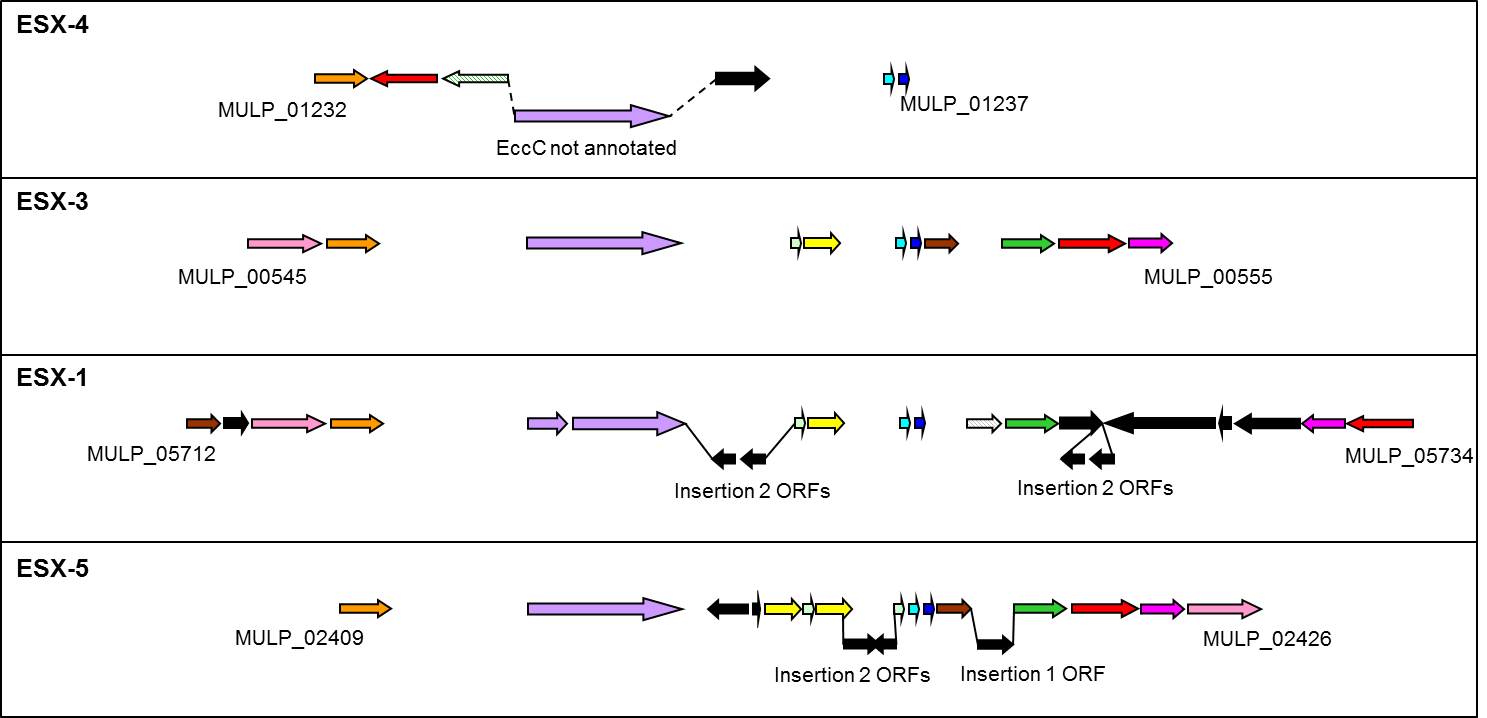
*

*M.ulcerans* subsp. *liflandii*

*
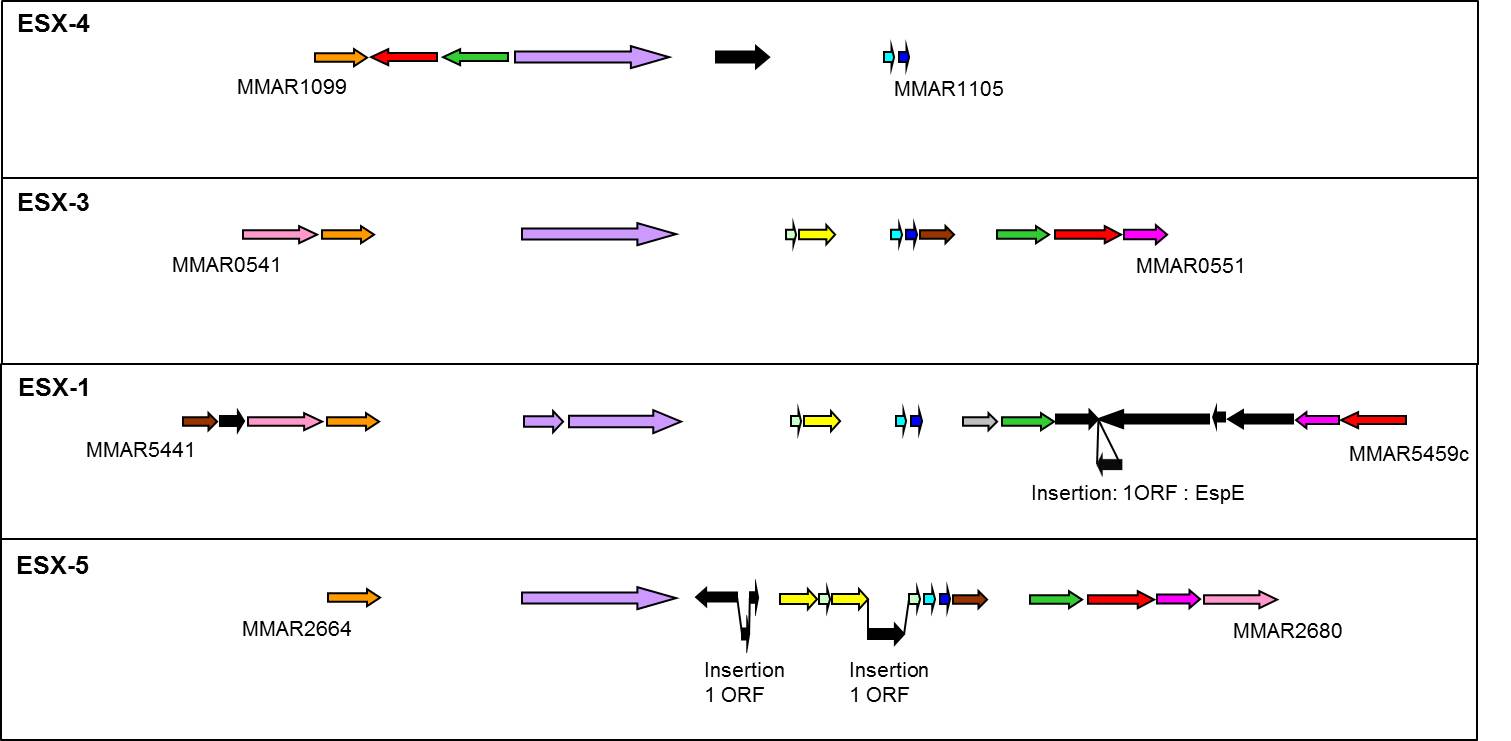
M. marinum*

*
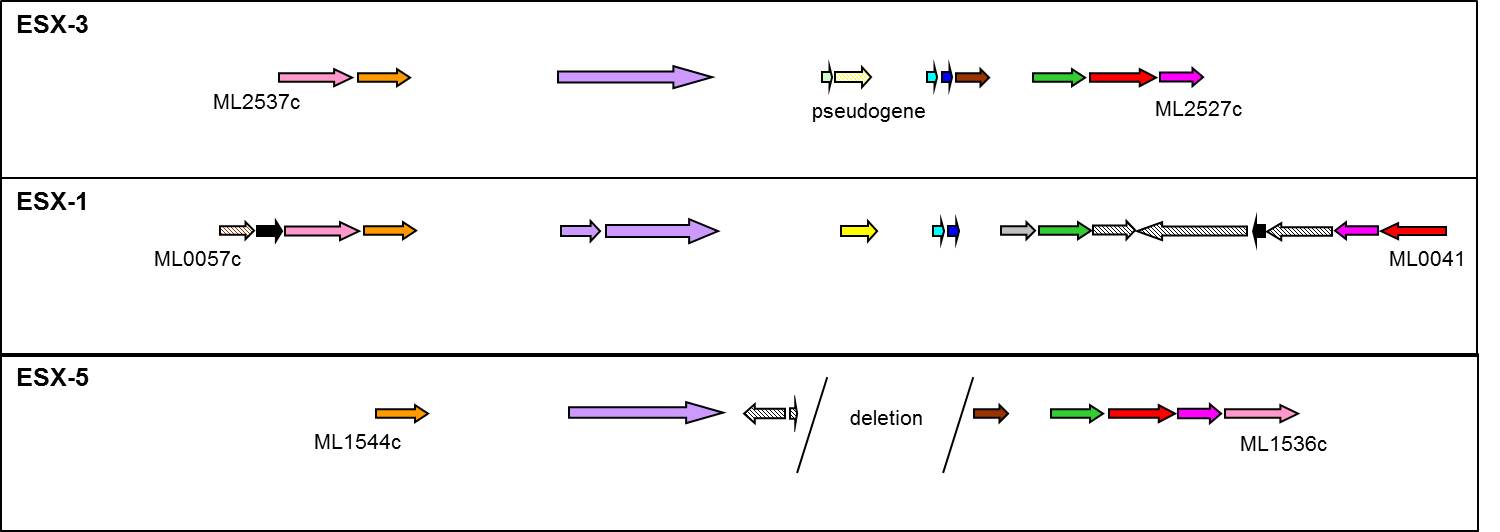
M. leprae*
