# Supplementary figures and images for: The plasmid-mediated evolution of the mycobacterial ESX (Type VII) secretion systems
Source: BMC Evol Biol. 2016 Mar 15;16:62. doi: 10.1186/s12862-016-0631-2 (PMC4791881; doi:10.1186/s12862-016-0631-2)

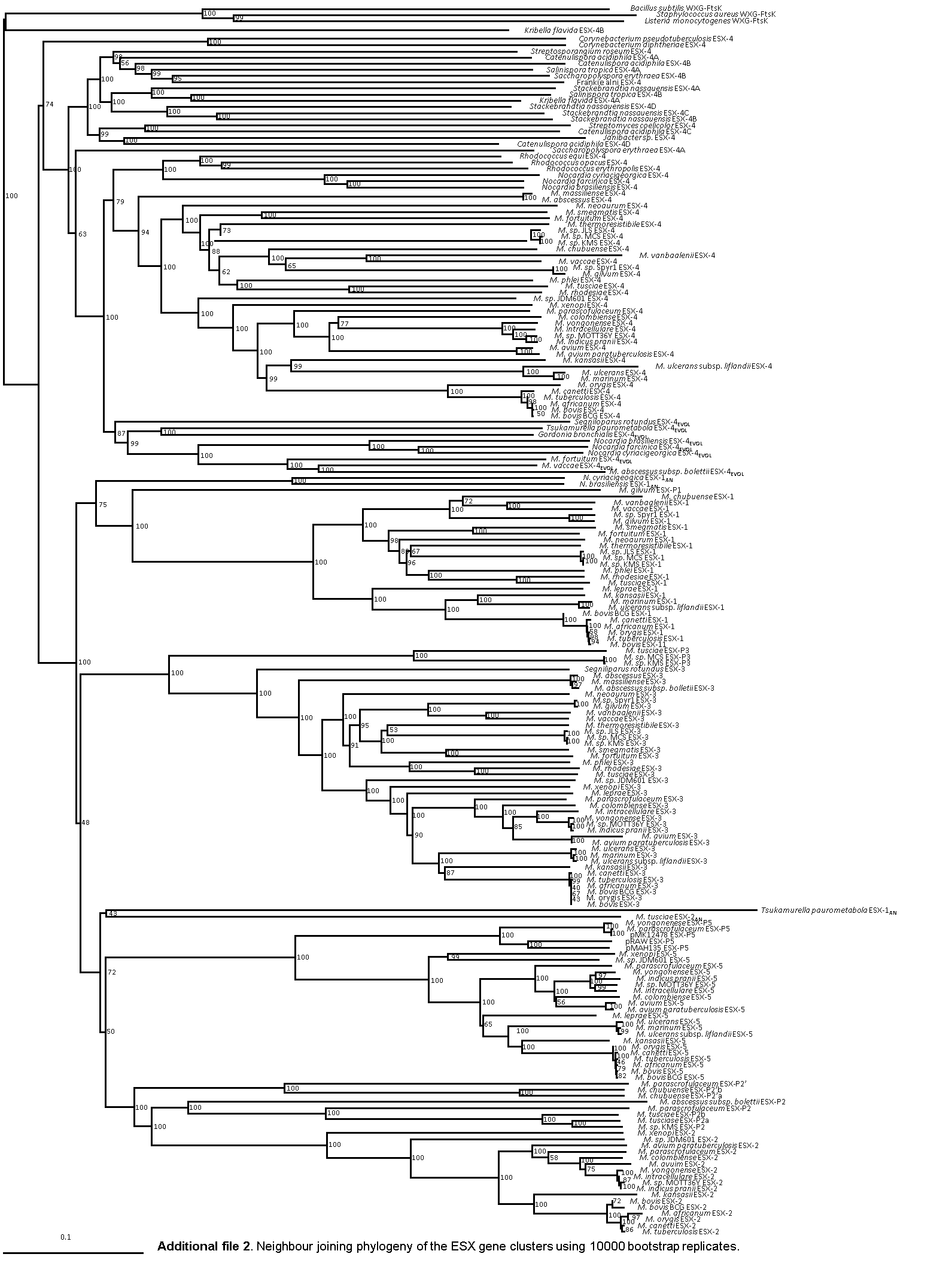

Supplement: Additional file 2: — Neighbour joining phylogeny of the ESX gene clusters using 10000 bootstrap replicates. (PNG 33 kb) [file 12862_2016_631_MOESM2_ESM.png]
