# Supplementary material for: The plasmid-mediated evolution of the mycobacterial ESX (Type VII) secretion systems
Source: BMC Evol Biol. 2016 Mar 15;16:62. doi: 10.1186/s12862-016-0631-2 (PMC4791881; doi:10.1186/s12862-016-0631-2)
Supplement: Additional file 3: — Mauve sequence alignments of ESX-P containing plasmids and contigs which are predicted to be plasmids. [file 12862_2016_631_MOESM3_ESM.pptx]

## Slide 1
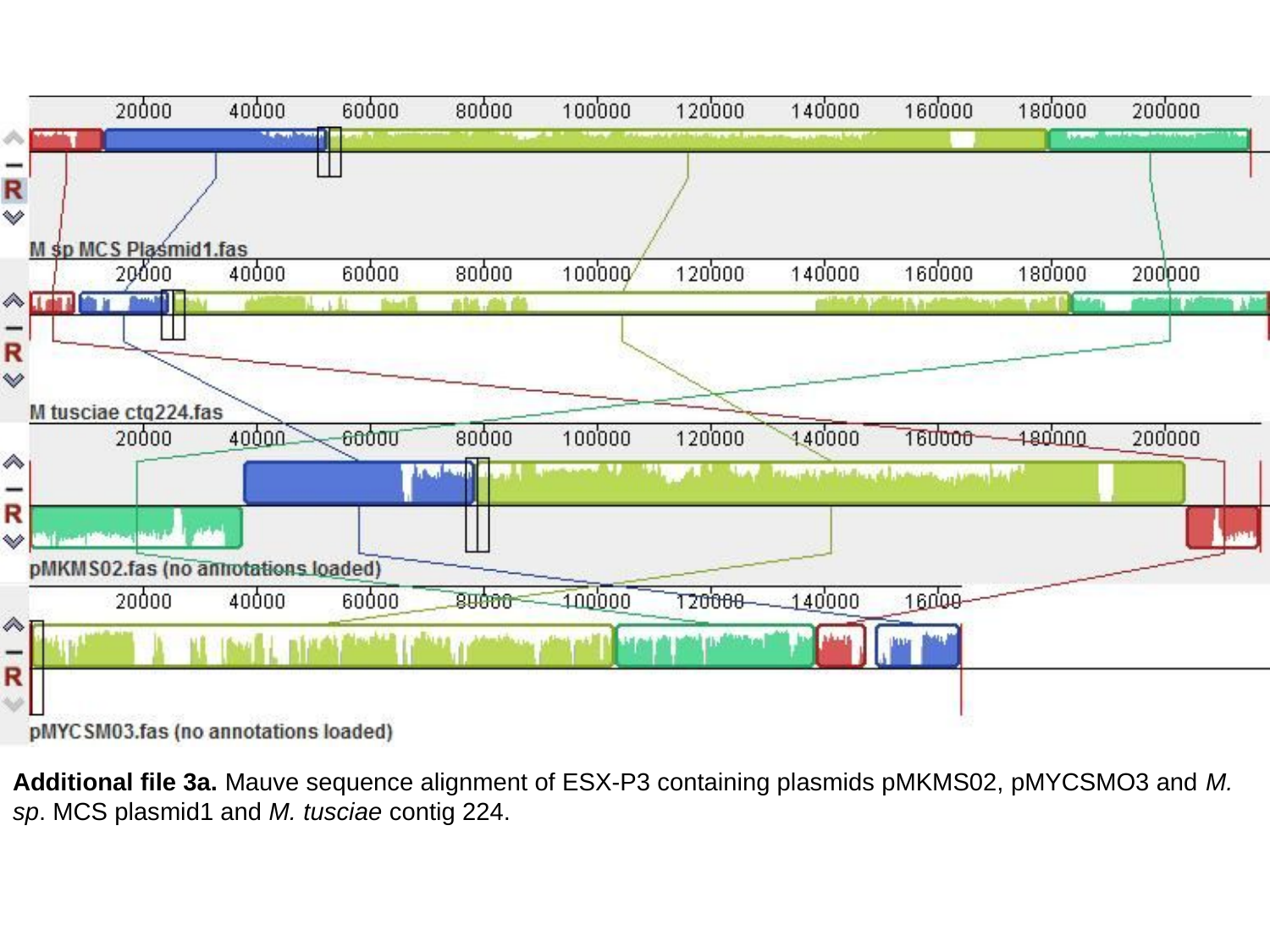

# Additional file 3a. Mauve sequence alignment of ESX-P3 containing plasmids pMKMS02, pMYCSMO3 and M. sp. MCS plasmid1 and M. tusciae contig 224.

## Slide 2
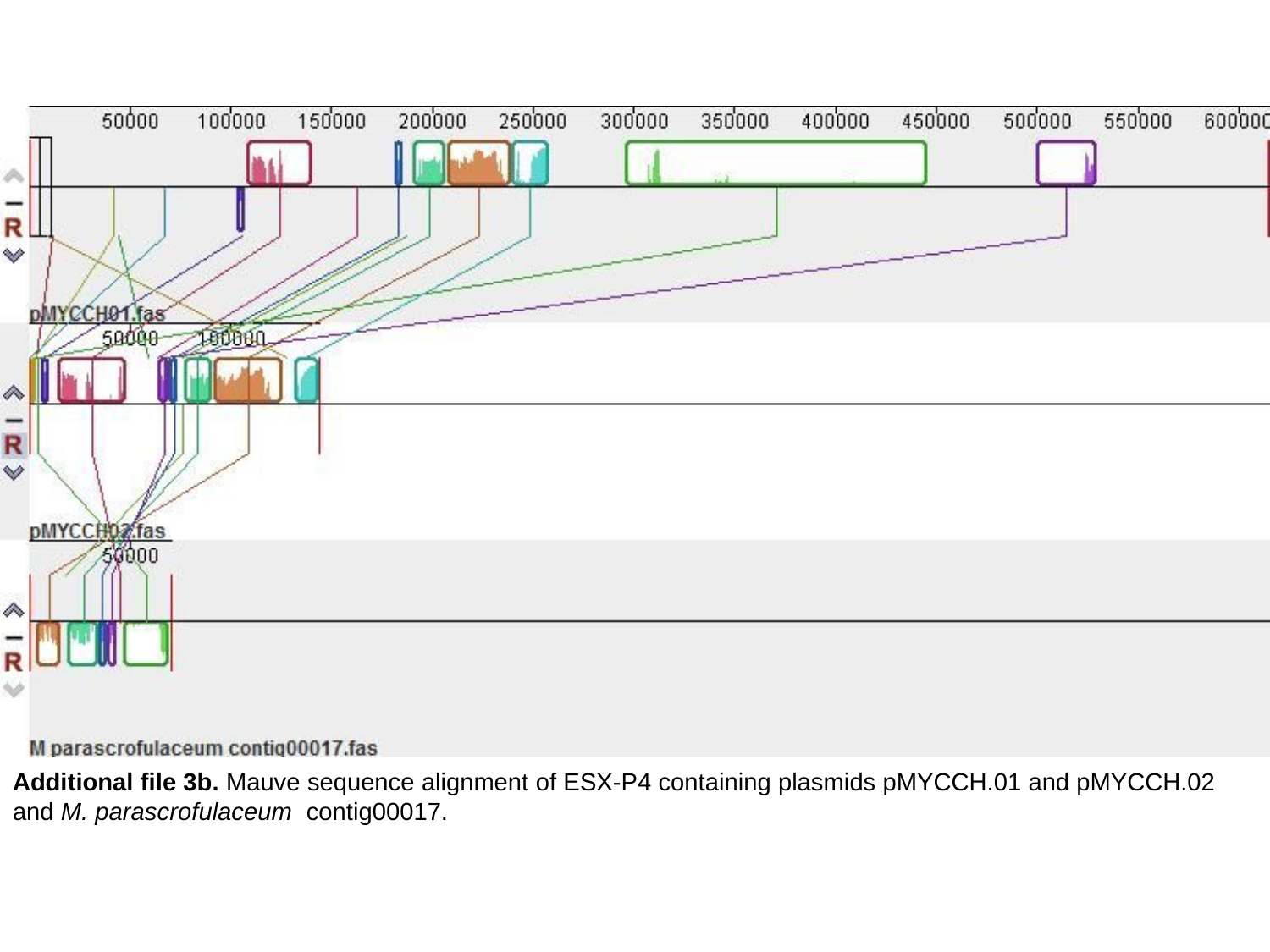

Additional file 3b. Mauve sequence alignment of ESX-P4 containing plasmids pMYCCH.01 and pMYCCH.02 and M. parascrofulaceum contig00017.

## Slide 3
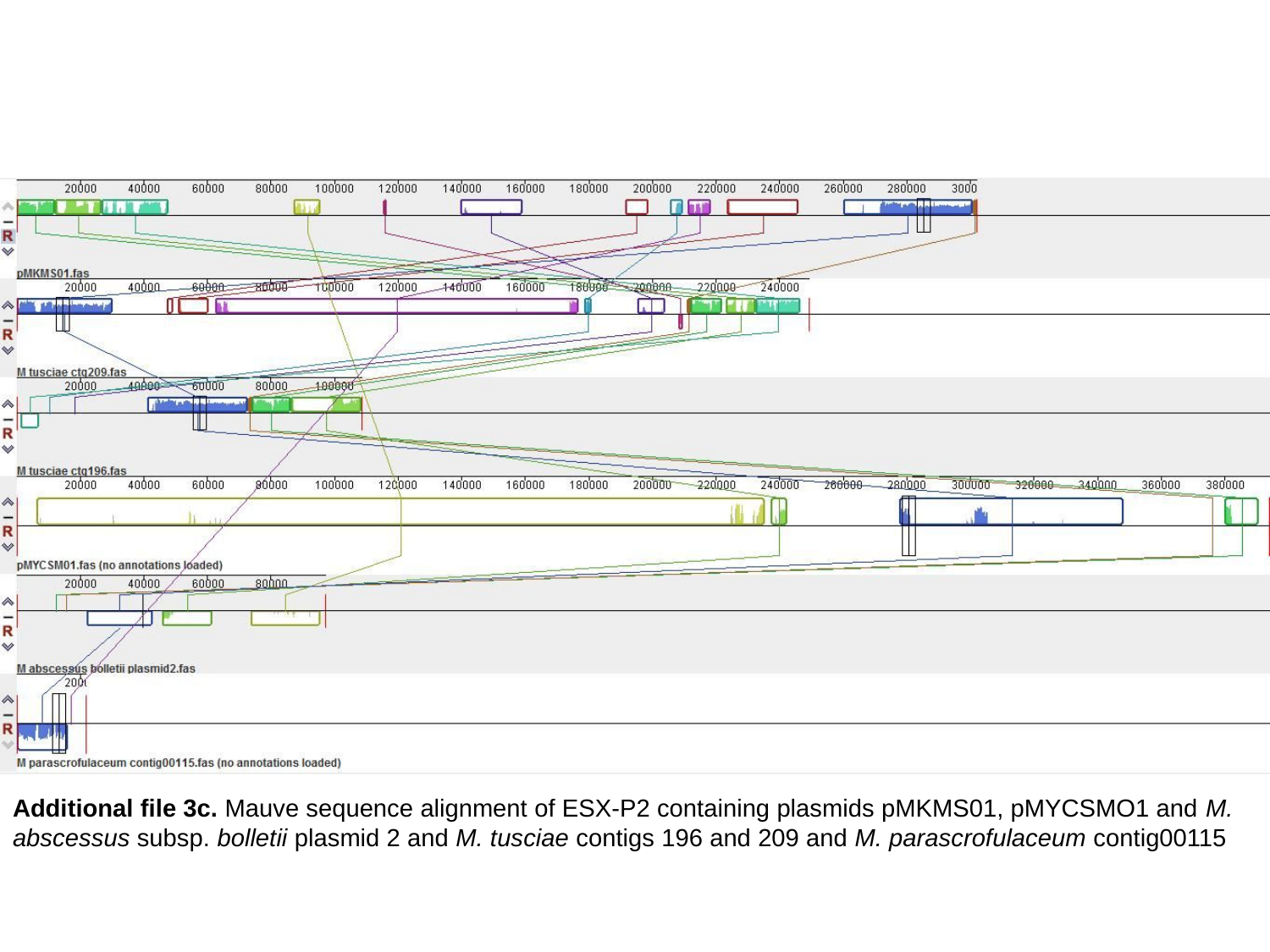

Additional file 3c. Mauve sequence alignment of ESX-P2 containing plasmids pMKMS01, pMYCSMO1 and M. abscessus subsp. bolletii plasmid 2 and M. tusciae contigs 196 and 209 and M. parascrofulaceum contig00115

## Slide 4
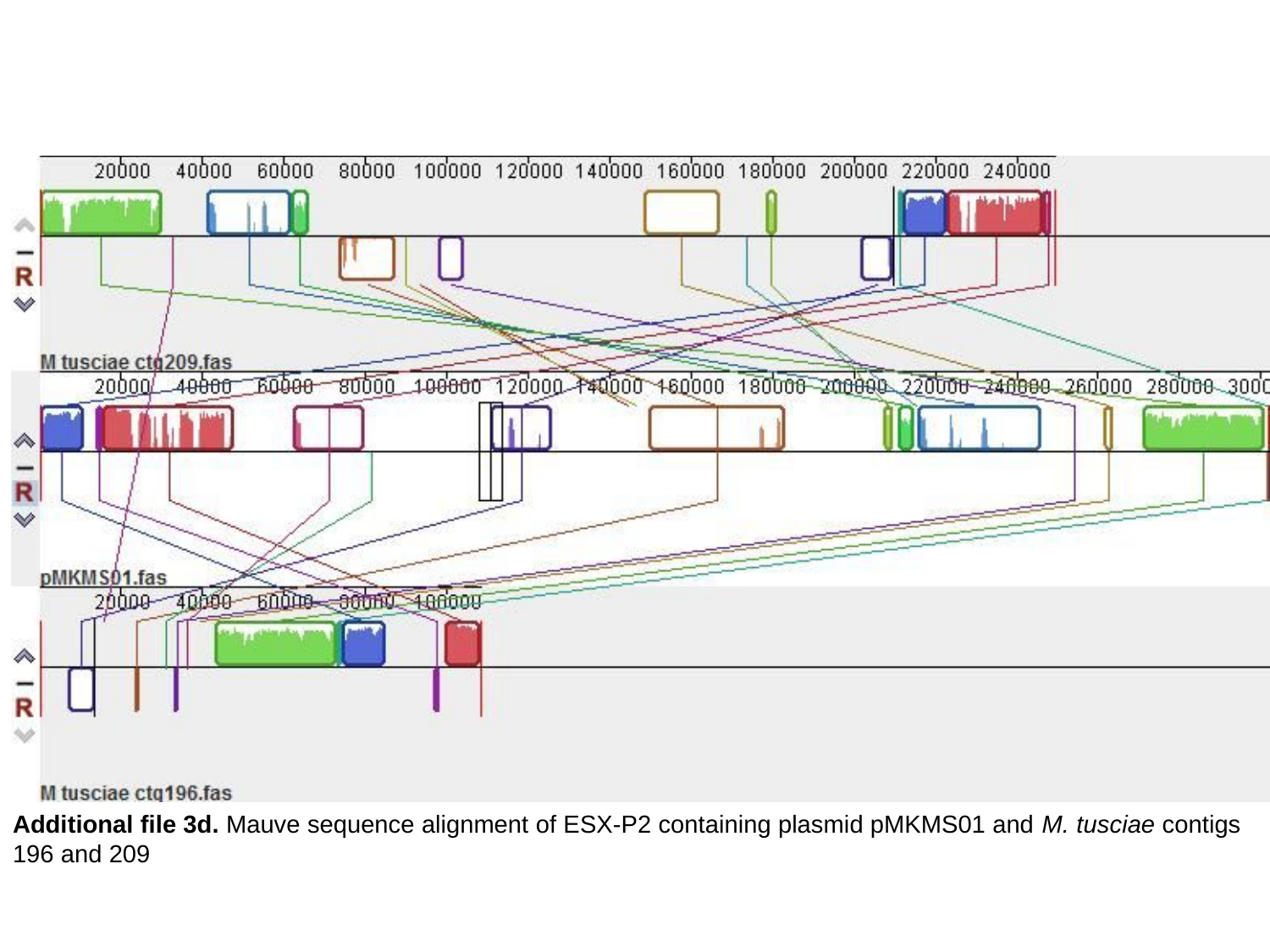

Additional file 3d. Mauve sequence alignment of ESX-P2 containing plasmid pMKMS01 and M. tusciae contigs 196 and 209

## Slide 5
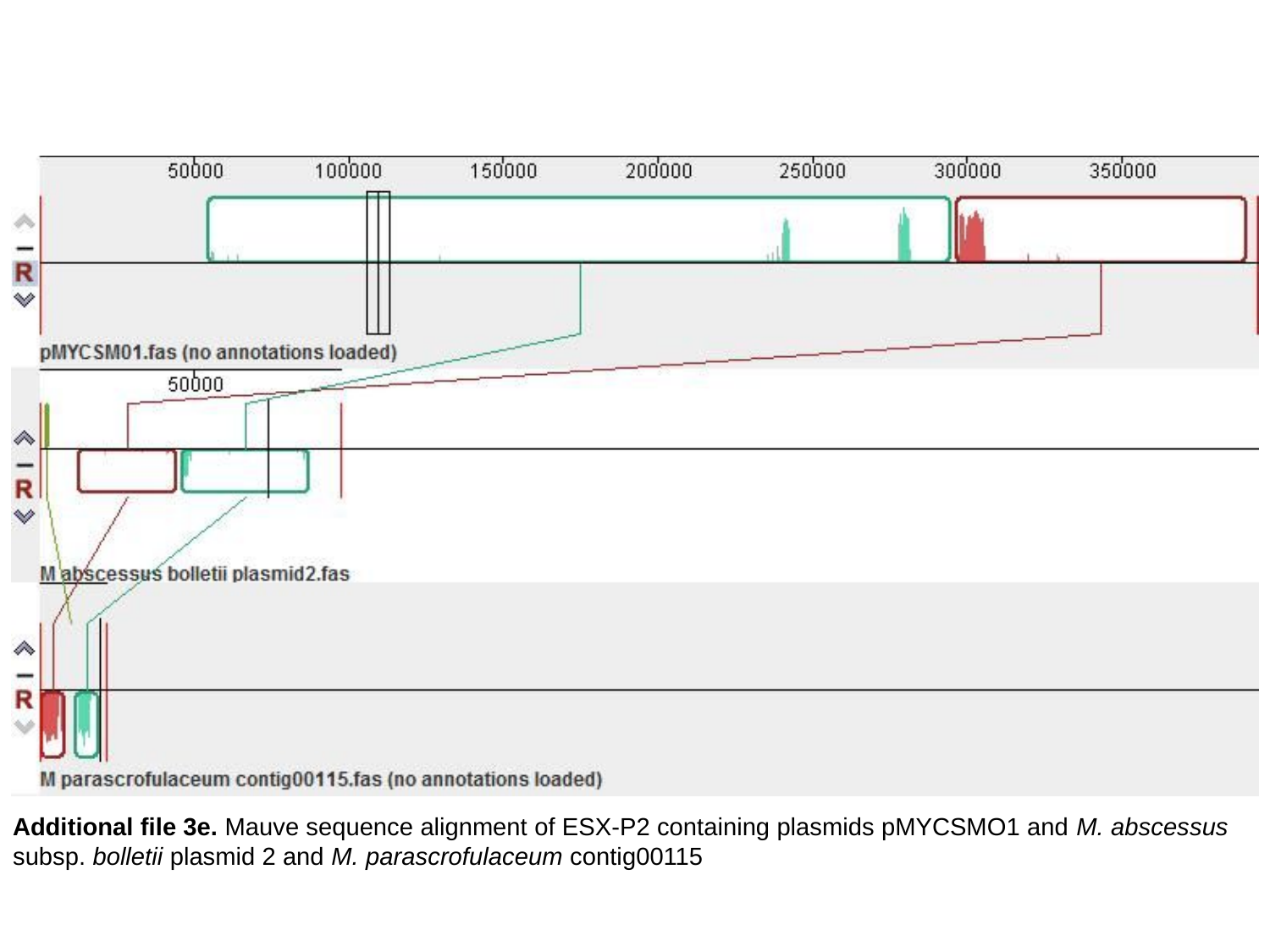

Additional file 3e. Mauve sequence alignment of ESX-P2 containing plasmids pMYCSMO1 and M. abscessus subsp. bolletii plasmid 2 and M. parascrofulaceum contig00115

## Slide 6
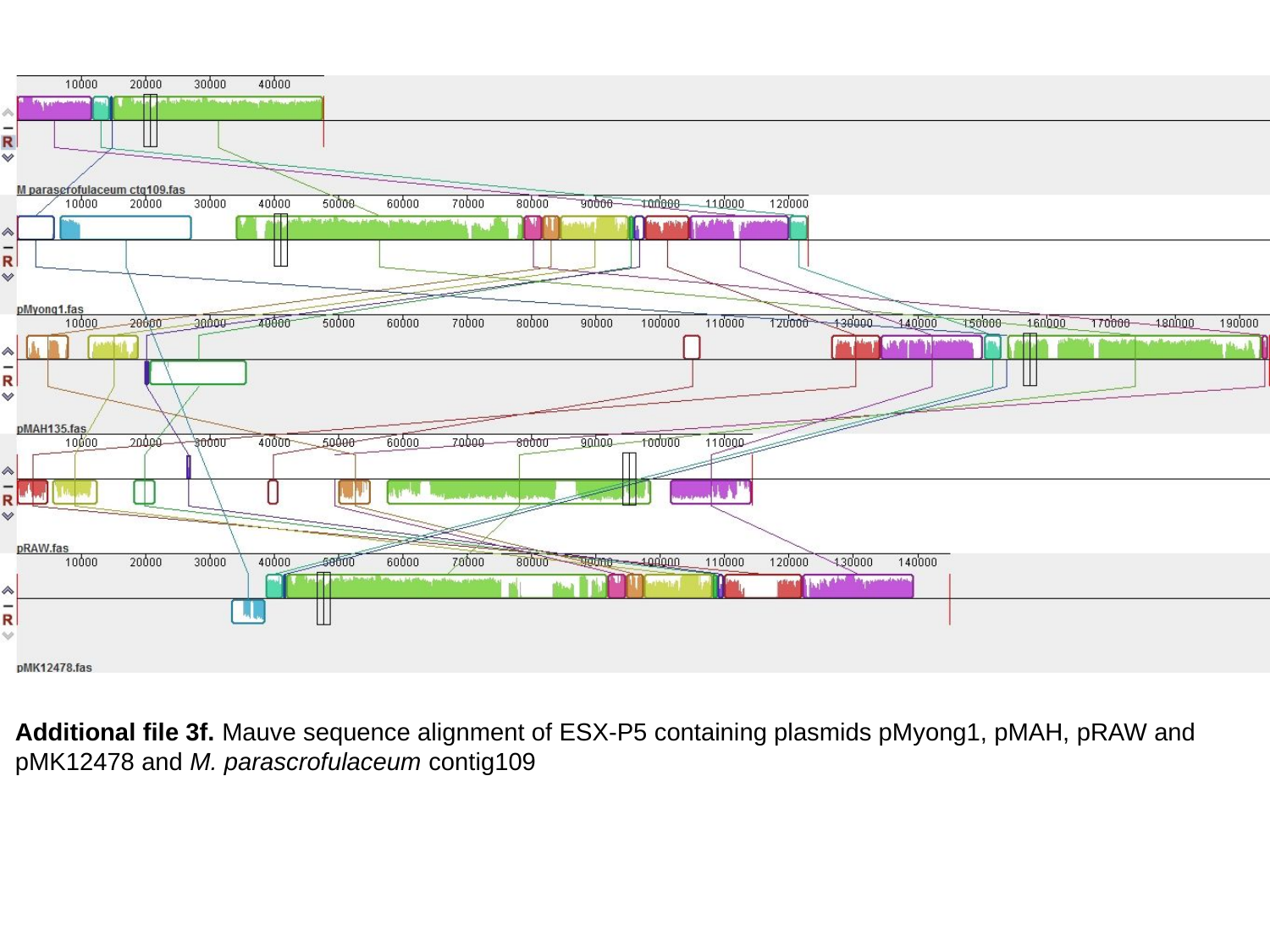

Additional file 3f. Mauve sequence alignment of ESX-P5 containing plasmids pMyong1, pMAH, pRAW and pMK12478 and M. parascrofulaceum contig109
